# Report of a Delphi exercise to inform the design of a research programme on screening for thoracic aortic disease

**DOI:** 10.1186/s13063-020-04562-1

**Published:** 2020-07-16

**Authors:** R. G. Abbasciano, J. Barwell, R. Sayers, M. Bown, D. Milewicz, G. Cooper, G. Mariscalco, N. Wheeldon, C. Fowler, G. Owens, G. J. Murphy, on behalf of the Aortic Dissection Awareness Day UK 2019 Working Group, Graham Cooper, Catherine Fowler, Mark Callaway, Rajesh Chelliah, Aparna Deshpande, Jeffrey Khoo, Gerry McCann, Praveen Rao, Matthew Bown, Dianna Milewicz, Huw Dorkins, Mark Field, Catherine Fletcher-Francis, Cliff Grover, Giovanni Mariscalco, Toru Suzuki, Amy Yasbeck, Julian Barwell, Karen English, Noelle Robertson, Anne Cotton, Sarah Gunn, Victoria McKay, Aung Oo, Deborah Osio, Nora Shannon, Saba Sharif, Nigel Wheeldon, Rob Sayers, Gareth Owens, Michael Sweeting, Cassandra Brookes, Tracy Kumar, Florence Lai, Gavin Murphy

**Affiliations:** 1 Department of Cardiovascular Sciences, University of Leicester, Clinical Sciences Wing, Glenfield General Hospital, Leicester, LE3 9QP UK; 2 Division of Medical Genetics, Department of Internal Medicine, The University of Texas Health Science Center at Houston, Houston, TX USA; 3 Northern General Hospital, Herries Road, Sheffield, UK; 4 https://aorticdissectionawareness.org/

**Keywords:** Cardiovascular surgery, Aortic dissection, Patient and public involvement, Public health

## Abstract

**Objectives:**

To inform the design of a clinical trial of a targeted screening programme for relatives of individuals affected by thoracic aortic disease, we performed a consensus exercise as to the acceptability of screening, the optimal sequence and choice of tests, long-term patient management, and choice of trial design.

**Methods:**

Working with the Aortic Dissection Awareness UK & Ireland patient association, we performed a Delphi exercise with clinical experts, patients, and carers, consisting of three rounds of consultation followed by a final multi-stakeholder face-to-face workshop.

**Results:**

Thirty-five experts and 84 members of the public took part in the surveys, with 164 patients and clinicians attending the final workshop. There was substantial agreement on the need for a targeted screening pathway that would employ a combined approach (imaging + genetic testing). The target population would include the first- and second-degree adult (> 15 years) relatives, with no upper age limit of affected patients. Disagreement persisted about the screening process, sequence, personnel, the imaging method to adopt, computed tomography (CT) scan vs magnetic resonance imaging (MRI), and the specifics of a potential trial, including willingness to undergo randomisation, and measures of effectiveness and acceptability.

**Conclusion:**

A Delphi process, initiated by patients, identified areas of uncertainty with respect to behaviour, process, and the design of a targeted screening programme for thoracic aortic disease that requires further research prior to any future trial.

## Introduction

Thoracic aortic disease is an uncommon condition that remains asymptomatic for many years until it presents as an acute aortic syndrome. These are often fatal, accounting for up to 5000 emergency hospital admissions and approximately 2000 deaths per year in the UK [1–3], more than road traffic accidents. The diagnosis of thoracic aortic disease during the latent stage in people with genetic syndromes such as Marfan syndrome or Ehlers-Danlos is facilitated by their distinct clinical characteristics. These are high-risk syndromes, where people develop aortic disease at an early age that if untreated often results in death. In these forms, intense surveillance, risk factor reduction, and early surgery are known to reduce mortality. Non-syndromic forms of thoracic aortic disease can also result in high-risk phenotypes. However, in the absence of pathognomonic clinical characteristics, these cases are often only detected incidentally or following emergent presentation. The long latency period, the ready availability of cross sectional imaging, and the emergence of high through-put genetic testing all support the introduction of targeted screening in people at risk of non-syndromic aortic disease. Imaging techniques can identify clinically silent aortic disease in up to 56% of asymptomatic relatives of patients with non-syndromic thoracic aortic disease [4]. Pathogenic variants are common even where there is no syndrome features or clear family history [4, 5]. It is therefore reasonable to assume that high-risk non-syndromic phenotypes will also benefit from intensive surveillance, strict blood pressure control, and early surgery. Routine screening is advocated by international treatment guidelines [6, 7] but is supported by low-level evidence. Moreover, the optimal design of an effective screening programme is not specified. To address this uncertainty, and as part of a patient-led initiative to reduce unwanted variation in the care of people at risk of death from aortic disease, we performed a Delphi exercise to identify the scope of a future research programme that would evaluate the acceptability, clinical effectiveness, and cost effectiveness of a targeted screening programme in at risk-populations.

## Methods

We performed a modified Delphi exercise [8, 9], to inform the study design of a clinical trial investigating screening requirements in thoracic aortic diseases (with a focus on non-syndromic forms). Published methodological criteria [1] for reporting Delphi studies were employed (Appendix 1 in the digital supplement). A planning committee pre-specified the number and structure of surveys, involved participants, consensus threshold, survey documentation, analysis plan, and contents of the questionnaires. The survey phases of the exercise were conducted in total anonymity; both the physical and digital versions of the survey did not allow storage of personal data. Nonetheless, respondents were reminded that their questions could be analysed in future rounds of the process. The planning committee informed the members of the expert panels about the aim of the Delphi process and their consent was required in an email before involvement. The online flow chart provided by the UK Health Research Authority (http://www.hra-decisiontools.org.uk/ethics/) determined our research project could be conducted without the need for ethics review board approval (Appendix 2 in online digital supplement).

### Participants

Participants were selected based on field of expertise, geographic area of practice, career stage, and interest in the topic, in order to maximise participants’ acceptance rates and heterogeneity of experience. The panel of clinical experts included cardiologists, cardiac surgeons, radiologists, clinical geneticists, genetic counsellors, clinical psychologists, statisticians, and other trial methodologists (see Appendix 2 in the online Digital supplement). Six carers of/and survivors of aortic dissection were invited by the national patients association Aortic Dissection Awareness UK & Ireland, to complete the expert panel to ensure patient and public involvement throughout the process. The expert panel had 35 members in total (8 for the imaging panel, 9 for the molecular genetics panel, 11 for the clinical genetics panel, 7 for the trial design panel). Although an ideal number of panel members is not established for a Delphi process, Diamond et al. report a similar numerical composition in most of the Delphi processes assessed in their systematic review [10].

To broaden the scope of the process, the questionnaire was also sent to the UK regional Inherited Cardiac Conditions (ICC) Services, to capture the views of this expert group. A modification of the questions for the expert panel was also disseminated digitally [11] to people and carers of those who had survived thoracic aortic dissection via the Aortic Dissection Awareness UK membership. The topics considered and the list of questions mirrored those present in the panellists’ questionnaire. The ICC Services Survey and the lay version of the questionnaire collected responses for the duration of the first and second surveys. These two surveys were purely advisory and were conducted in order to obtain a description of what is the current standard of care for thoracic aortic disease screening, and then presented to the panel members to assist with decision making prior to and during the final workshops (Appendix 3 in the online digital supplement).

### Formulating the research questions

An extended narrative that incorporates excerpts from the protocol we adopted to plan the Delphi is available in Appendix 1 in the online digital supplement. Areas of uncertainty to be addressed by the Delphi process are described in Table 1. The questionnaire was preceded by a pilot survey in 49 people prior to being opened to experts and the public to assess usability and ease of access. The results of the pilot survey are available in Appendix 4 in the online only digital supplement.

**Table 1:** Summary of areas of consensus and disagreement with respect to the design of a clinical trial evaluating the effectiveness of targeted screening for thoracic aortic disease

Area	Question	Summary answer
Imaging	Should relatives of patients affected by non-syndromic aortic disease undergo an imaging test?	**Yes (95%)**
	Which imaging test should be used in cases in which no clear genetic condition can be identified?	**MRI (79%)** **Echocardiogram (21%)** **CT scan (21%)**
	Which imaging test should be employed in cases in which a genetic condition can be identified?	**MRI (82%)** **Echocardiogram (36%)** **CT scan (45%)**
	What should be the method of choice for follow-up in relatives with an uncertain genetic variant?	**MRI (84%)** **Echocardiogram (21%)** **CT scan (16%)**
	Starting from what age should relatives be screened with an imaging test?	16 years (19%) 18 years (19%) 10 years before (19%)
	What should be the optimal follow-up rate?	1 year (70–100%) Consensus not reached for: -Family history (SDR)
Genetic testing	Should incidental findings be a reason to adopt a more focused test?	**Yes (95%)**
	Who should be involved in genetic screening?	**FDR (100%)** **SDR (45%)** **TDR (10%)**
	When would a patient (with a previous negative or inconclusive genetic test result) require re-testing?	**Newly diagnosed FDR (95%)** **New evidence of pathogenic variants (100%)**
	Is it appropriate to store a sample from a patient affected by aortic dissection in any case during an urgent operation, for the purpose of genetic testing?	**Yes (95%)**
	Is it appropriate to discuss genetic testing with the family after an urgent surgery for aortic dissection?	**Yes (95%)**
	Is it appropriate to discuss genetic testing with the family after a patient dies from aortic dissection?	**Yes (100%)**
Genetic counselling	Who should be the professional figure involved in informing patients about genetic risk (and therefore referring them to a clinical geneticist)?	**Cardiac surgeon (69%)**
	Should a multidisciplinary team be involved in the management of these families? What professional figures should be involved from the outset?	**Clinical geneticist (100%)** **Cardiac surgeon (95%)** **Cardiologist (90%)** **Radiologist (84%)** **Psychologist (69%)**
	How many years before the youngest person dissects for that gene should we start surveillance?	5 years (33%) 10 years (47%)
	Regarding the age peak in the risk of dissection, is it best to consider the mean value or the lowest one to plan screening?	**Youngest age at dissection (89%)**
	Should there be an upper age limit for offering genetic testing to the patient with a thoracic aortic disease?	**No (79%)** **Yes (21%)**
	Which upper age limit should be considered?	Mean (SD) 72.9 (10.88)
	Which psychological tests should be used to monitor the impact of the screening programme? (Depression)	HADS (30%) WHO WMH-CIDI (30%)
	Which psychological tests should be used to monitor the impact of the screening programme? (Anxiety)	HAM-A (44.4%)
Trial design	What would be the optimal trial design to use to assess the value of a screening programme for TADs?	Cluster (50%) Stepped wedge (22%) Individual randomisation (28%)
	How many centres should be involved?	**More than 7 (60%)**
	How long do you think it would take to change what is currently done for screening?	More than 2 years (57%) 2 years (21%)
	What tool should be used to measure quality of life?	EQ-5D (47%) SF-36 (13%)
	What are the most appropriate measures of effectiveness?	**New diagnosed disease (70%)** **Long-term mortality (73%)**
	Which clinical events should be evaluated in this research?	**Perioperative mortality (94%)** **AMI and stroke (83%)** **Length of stay (67%)** **AKI (61%)**
	When should relatives involved in the test be monitored for signs of depression and anxiety?	12 months (52%) 12 months (52%)

*MRI* magnetic resonance imaging, *CT* computed tomography, *FDR* first-degree relatives, *SDR* second-degree relatives, *TDR* third-degree relatives, *HADS* Hospital Anxiety and Depression Scale, *WHO WMH-CIDI* World Health Organization World Mental Health Composite International Diagnostic Interview, *HAM-A* Hamilton Anxiety Rating Scale, *EQ-5D* Euro QoL 5 dimensions, *SF-36* Short Form 36, *AMI* acute myocardial infarction, *AKI* acute kidney injury

### Surveys

#### Survey 1

A paper-based and an online survey [11] were launched during the Society for Cardiothoracic Surgery in the UK and Ireland Annual Scientific Conference (10–12 March 2019) and on AD Awareness UK and Ireland websites and Social Network pages and then circulated over a 2-month period (from March 2019 to May 2019). The survey captured interested parties’ questions around four main areas of aortic disease research that are especially relevant for screening: imaging, genetic testing, clinical genetics/genetic counselling, and screening evaluation/trial design. After a review of the responses, the list of questions was subsequently filtered and analysed by the planning committee to form the layout for the second round.

#### Survey 2

Survey 2 was conducted from May 2019 to July 2019. For the second survey, data from the open-ended questions in the first survey was presented with tag clouds and radar charts, along with a summary of the responses from all the respondents. During round 1, participants were asked to use a digital survey to state their opinion, by rating statements, answering questions, or interpreting results. The resulting submissions were collected and summarised (Appendix 5 in the online only digital supplement), and then presented to the experts. During round 2, from July 2019 to September 2019, panel members were asked to use a revised questionnaire to provide their responses, eventually reconsidering their views, after reading the report. Disagreeing with the current consensus required to add a motivation to the answer provided. Exploring disagreement prevented excessive dissenter drop out and generation of an artificial consensus [8].

**Fig. 1:**
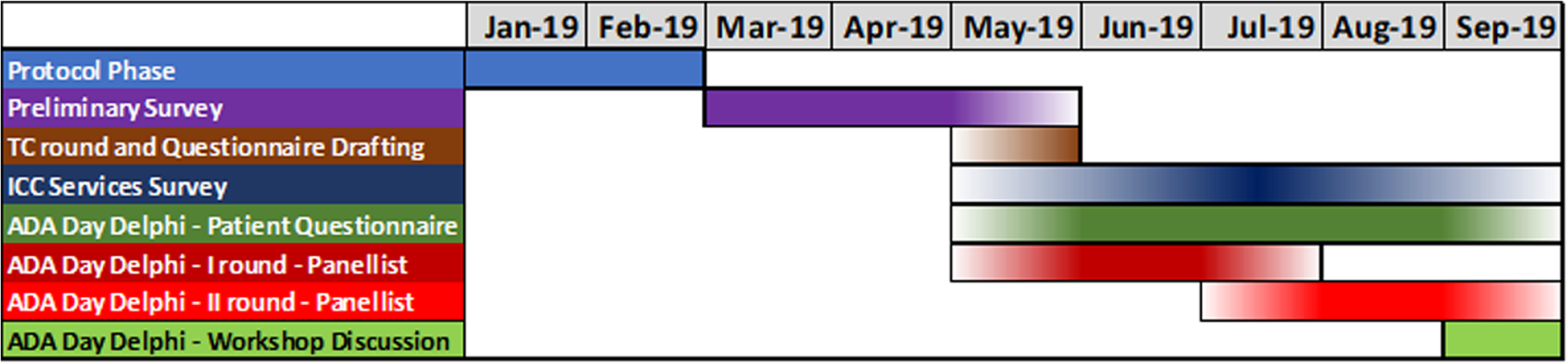
Aortic Dissection Awareness Day UK 2019 Delphi—timeline. TC, teleconferences; ADA, Aortic Dissection Awareness

A cut-off value of 60% was used to define consensus at the end of the second round. The timeline of the initiative is presented in Fig. 1.

### Final workshops

Remaining areas of disagreement were addressed during round 3, conducted as 4 workshops (1 for each theme developed in the questionnaire: imaging, genetic testing, clinical genetics, trial design) as part of the Aortic Dissection Awareness Day annual meeting on 19 September 2019. The meeting was attended by approximately 110 aortic dissection survivors and carers. Each workshop was moderated by 1 clinician and 1 member of the public with a public: clinician ratio of 3:1. Members of the public were allocated according to their individual preferences. Results from the Delphi surveys were presented by a moderator with the help of specifically designed posters (available in Appendix 6 in the online only digital supplement). Attendants were offered the possibility to comment on the result and to offer their perspective and personal experiences on the design of a proposed research study for each theme. Consensus, if reached, was not necessarily modified by further discussion; however, the face-to-face meeting gave the participants involved an opportunity to ask questions and raise concerns that are taken into consideration in this dissemination of the output from the conference and from the Delphi. At the end of the conference, results from the whole process (including a summary of the workshops’ discussions) were presented to all attendees.

## Results

Twenty-eight expert panellists took part in the first round and 21 responded to the second survey. Six ICC Services responded. A total of 84 people responded to the lay questionnaire. The intermediate results from round 1 are available in Appendix 5. Results from the whole initiative, presented in a graphical form that could facilitate discussion on the conference day, are available in Appendix 6. Thirty-five expert panellists and around 110 aortic dissection survivors and carers attended the final workshops.

Consensus was reached for 17 out of the 27 uncertainties presented in the questionnaire. Areas of consensus and areas of residual disagreement for the whole Delphi exercise are summarised in Table 1.

### Imaging

**Fig. 2:**
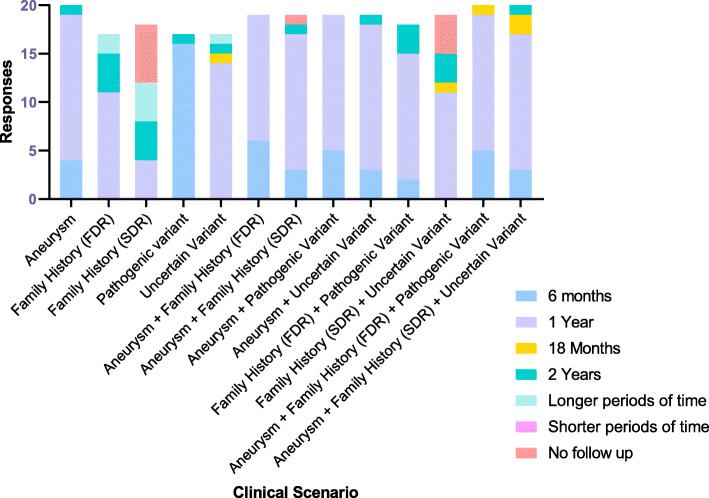
Different follow-up rates for various clinical scenarios ranked by the panel members during the second round of the survey. FDR, first-degree relative; SDR, second-degree relative

Consensus (72%) was obtained about the necessity of combining a genetic and imaging approach in screening. Twenty-eight percent of the participants thought an imaging test was required only in case of a positive genetic test, with a preference for magnetic resonance imaging (MRI) as the method of investigation, both in presence or absence of an underlying genetic mutation (76% and 84%, respectively) for the expert survey. Sixty-five percent of respondents to the lay survey selected computed tomography (CT) as the preferred modality. More diverse opinions were obtained regarding follow-up. For patients with an uncertain genetic variant, 68% and 52% of the members thought MRI and echocardiography respectively were the best methods of choice for follow-up while preference for CT was 20%. There was disagreement with respect to the timing of follow-up rates of those with positive tests based on differences in family history, imaging, and genetic criteria, although the common period was 1 year (Fig. 2).

In the second survey, only 5% of the participants thought an imaging test was required exclusively in case of a positive genetic test; MRI was still the preferred method of investigation, in presence or absence of an underlying genetic mutation (82% and 79%, respectively) and in presence of an uncertain genetic variant (84%) with echocardiogram decreasing to 21% and CT to 16% of the choices. The most frequently adopted follow-up rate was once again 1 year. No consensus could be obtained related to the optimal follow-up in the scenario of a history of thoracic aortic disease (TAD) in a second-degree relative (SDR) as the only risk factor (1-year and 2-year follow-ups were considered acceptable by 24%, longer periods by 29%, and no follow-up by 42%).

The conclusions of the final workshop were that all first-degree relatives (FDR) of people with thoracic aortic disease should undergo screening with MRI. There was residual disagreement with respect to the need for imaging in SDR and the age at which imaging tests should be commenced in relation to birth or observed dissection age in the affected relative.

### Genetic testing

In the first survey, consensus was obtained regarding the necessity to adopt more focused genetic tests due to the possibility of incidental findings, as in the potential diagnosis of conditions (like predisposition to neoplasms or neurodegenerative conditions) unrelated to the thoracic aorta (87%) and the potential participants in the screening programme (100% of experts identified FDR as needing genetic screening). Starting screening from SDRs and third-degree relatives (TDR) (when no FDR could be identified) was considered appropriate by 54% and 6% of respondents respectively. New diagnosis in FDR (95%) and new scientific evidence of pathogenic variants (100%) were considered the most relevant reason to repeat a genetic test in a patient that was previously negative for it. Ninety-one percent of respondents considered it appropriate to store a blood sample from a patient undergoing urgent surgery for the purpose of genetic testing, and all the panel members agreed it was appropriate to discuss genetic testing with the families of all patients with acute aortic syndrome, regardless of the outcome of the surgery.

**Fig. 3:**
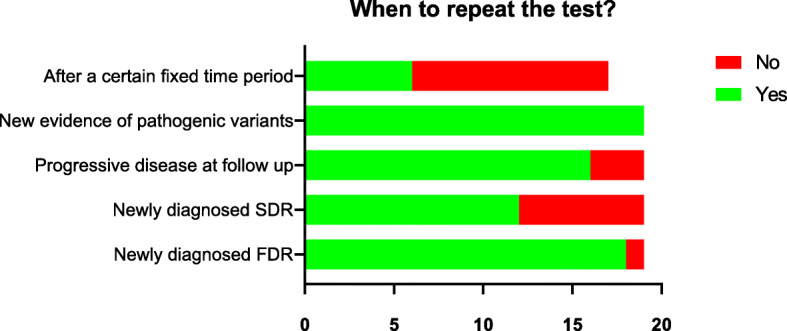
Reasons to repeat a genetic test in a previously negative patient as ranked by the panel members during the second round of the survey. FDR, first-degree relative; SDR, second-degree relative

In the second survey, incidental findings were considered a reason to adopt a more focused test by 95% of the respondents in the second round. The need for genetic screening in SDRs or TDRs, if testing in a FDR was negative, decreased to 45% and 10% respectively. Similarly to the first round, new diagnosis in FDR (95%) or new scientific evidence of pathogenic variants (100%) was considered as reasons to repeat a genetic test in a subject with a previously negative test, followed by imaging evidence of disease progression at follow-up (89%) and new diagnosis in a SDR (63%) (Fig. 3). Consensus was confirmed on the appropriateness of storing a sample for the purpose of genetic testing during surgery (95%), discussing genetic testing after an urgent surgery (95%), even in the case of the patient’s demise (100%).

In the final workshop, there was consensus in support of cascade screening adopting a pathway already in place for other cardiovascular genetic conditions such as hypertrophic obstructive cardiomyopathy. The consensus about the consent process was reinforced by patients’ feedback recommending a timely discussion with patients and families, even if this could be a cause of distress.

### Genetic counselling

During the first round, there was disagreement as to the roles of different professional groups in a potential screening programme. Cardiac surgeons (39%) and cardiologists (22%) ranked as the first professional figures that should be responsible for referring patients to a clinical geneticist. Thirty-three percent of patients included the general practitioner (GP) as the first point of referral. The ideal composition of the decision-making multi-disciplinary team (MDT) was clinical genetics (92%), cardiac surgeon (88%), cardiologist (88%), radiologist (75%), and clinical psychologist (58%). An overall consensus was reached on the age criteria for inclusion in the screening programme. Seventy percent of respondents opted for the youngest age at dissection as the best criteria, compared to the mean age; 5 years (35%) and 10 years (35%) before the youngest person dissects for that gene were considered the optimal period to start surveillance. Sixty-three percent of respondents thought an upper age limit should not be considered when selecting patients to be considered for genetic testing.

No defined consensus was obtained for psychological monitoring during screening. The Hospital Anxiety and Depression Scale (HADS) (30%) and the World Health Organization World Mental Health Composite International Diagnostic Interview (WHO WMH-CIDI) (30%) were selected as the most relevant tools for psychological monitoring during screening.

During the second survey, cardiac surgeons’ choice as the first professional figures to liaise families with the clinical geneticist rose to 68%, while cardiologists were selected by 16% of the respondents. There was good consensus as to the ideal MDT composition: clinical geneticist (100%), cardiac surgeon (95%), cardiologist (90%), radiologist (84%), and psychologist (69%). Youngest age at dissection for each genotype was again considered the best parameter to plan screening (90%). There was no agreement on the age or stage of disease (family history dissection age or genotype) as when to start surveillance. Consensus about avoiding upper age limit increased to 79%.

The final workshop could not resolve residual disagreement on the lower age limits for screening in the absence of familial dissection and whether this should be determined by genotype and the type of tests for monitoring the impact of screening on psychological health (depression and anxiety). The target population identified by both clinicians and members of the public was comparable, with a substantial agreement in terms of offering a test combining an imaging and genetic approach. The possibility of being diagnosed with an uncertain variant did not seem to be an element of concerns for patients and carers during the discussion, as long as the initial test was accompanied by adequate support in terms of counselling and psychological monitoring.

### Trial design

In the first survey, suggestions for the optimal trial design for a study investigating a potential screening program were split almost equally between cluster randomisation (33.3%), individual randomisation (33.3%), and stepped wedge (29.2%). Individual randomisation was the preferred design (61.4%) in the patients’ survey. As for the other specifics of a potential trial, 72.8% of respondents thought such a trial would need to involve 7 or more aortic centres, and 50% of responses indicated a change in their regions’ practice would require more than 2 years to undertake such a trial. *Euro QoL 5 dimensions* (EQ-5D) was selected as the optimal tool to measure quality of life (40%), followed by Short Form (36) Health Survey (SF-36) (20%). New diagnosis and reduction in long-term mortality were rated as the essential measures of effectiveness, followed by cost-effectiveness and higher number of patients treated; among patients, new diagnosis ranked first and cost-effectiveness last.

In the second survey, cluster randomisation (50%) became the most common choice in terms of trial design. Preferences in terms of measures of effectiveness remained unaltered.

In the final workshop, discussion focused largely on the willingness of participants to being randomised to a control group in any randomised controlled trial (RCT). This had not been part of the initial survey, but there was general consensus among patients that they did not consider randomisation to a no screening arm of a RCT to be ethical. Other remaining uncertainties included how to address ignorance as to the genetic basis of thoracic aortic disease by both health professionals and members of the public as a means of improving uptake, what the optimal treatment pathways should be for people who screen positive or negative for the disease with respect to genotype and phenotype, and the best tools for quality of life and psychological monitoring as part of a trial. There was consensus that these issues should be addressed by further research. A final area of consensus that arose during the workshop was that tissue samples should be obtained from all patients undergoing surgery for thoracic aortic disease to assist with further research.

## Discussion

### Main findings

**Fig. 4:**
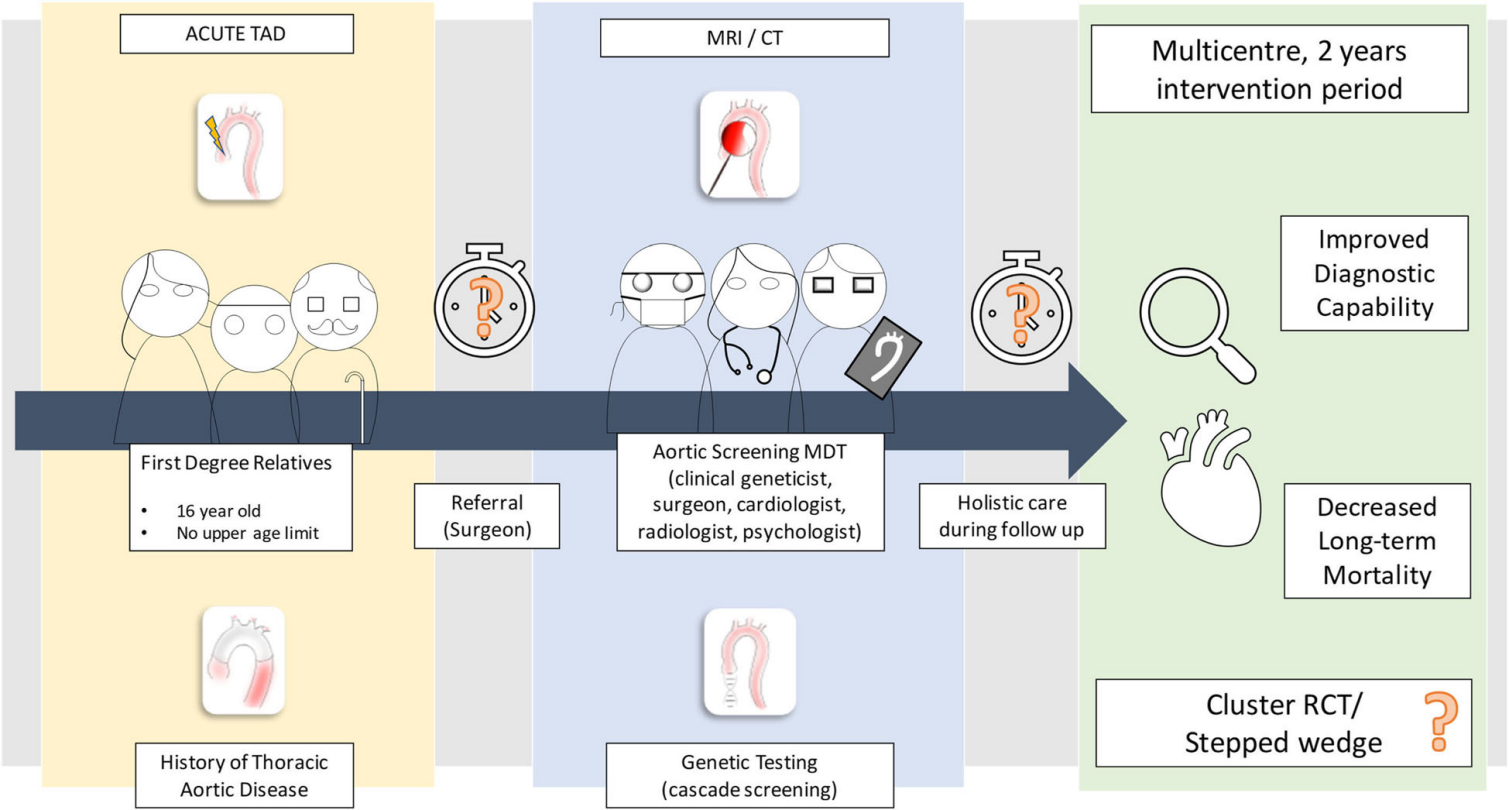
Aortic Dissection Awareness Delphi Research Proposal. A research programme based on the findings of the Delphi would evaluate a screening programme targeting first-degree relatives of patients affected by thoracic aortic diseases and offering them (after referral by a surgeon) a combined (screening + genetic) intervention, coordinated by a multidisciplinary group (composed by clinical geneticist, surgeon, cardiologist, radiologist, and psychologist). Uncertainties persist around timing considerations (age at which imaging should be offered, follow-up in different groups) methodological and design considerations for such research programme. TAD, thoracic aortic disease; MRI, magnetic resonance imaging, CT, computed tomography, MDT, multidisciplinary team, RCT, randomised controlled trial

A patient and public involvement Delphi exercise conducted in partnership with the Aortic Dissection Awareness UK patient organisation and the UK NHS Inherited Cardiac Conditions network identified key areas of consensus with respect to the design of a research programme that aims to provide high quality evidence to support the introduction of routine targeted screening for thoracic aortic disease (Fig. 4). There was broad consensus that a screening programme should include MRI and genetic screening of the FDR of people with thoracic aortic disease. There was consensus that the surgical team should be required to initiate the referral to the screening programme with discussions with relatives at an early stage even in the event of death of the proband. People undergoing screening should be managed by multidisciplinary teams of clinicians and psychologists with regular follow-up at least annually and further focused testing in the event of new clinical events in relatives or the presence of incidental findings that may herald more aggressive phenotypes. There was agreement that the primary evaluation of the effectiveness of the screening programme should focus on the identification of new cases of disease (genotype or phenotype) with assessment of major adverse cardiovascular events, quality of life, and healthcare resource use as secondary outcomes. It is clear that people will expect a screening programme capable of following the entire ‘patient pathway’ from the initial approach to the eventual therapeutic intervention or to potentially life-long surveillance.

Residual disagreements include the value of screening in SDR and TDR where tests in FDR are negative, the precise genetic panel to be tested, the optimal treatment pathways for people who test positive or test negative, the acceptability of randomisation to no screening in any future trial, and the optimal tools for measuring the effects of screening on psychological wellbeing. These topics will be addressed through a further series of focus groups that will bring together researchers with patients and clinicians.

Public and experts’ opinion were split concerning the preferred imaging technique. It is the authors’ opinion that patients’ preference for the CT scan may be partially due to the excellent ‘Think Aorta’ campaign run by Aortic Dissection Awareness UK that focused on the need for a CT scan in the Access and Emergency Department as the gold standard to diagnose or exclude an aortic dissection. Nonetheless, one must not ignore the acceptability aspects of the MRI exam (claustrophobia and anxiety), and this issue has been observed in the cohort of a pilot study currently undergoing at our centre (unpublished, NCT03861741).

There was strong consensus among clinicians and patients that relatives of those with aortic disease should undergo routine imaging in addition to genetic testing, so as to identify the relatives at risk even in case of a negative genetic test, a circumstance that may happen, as shown in a recent systematic review, in up to 70% of familial forms of non-syndromic thoracic aortic diseases [4]. It is expected that this percentage will drop as the number of causative genes identified in thoracic aortic diseases increases. As for the likelihood that the genetic or imaging tests may have incidental findings, such as for example elevated cancer risk, this was not considered a major concern, as long as an adequate counselling process was ensured. This also underlined the importance of the delivery of a holistic care package during the follow-up period.

The trial design questions in our process were the ones characterised by the highest amount of disagreement. The crucial issue revolved around the randomisation process. While methodologist and experts recognised the importance of randomisation in adequately powering the potential trial, discussion during the workshop showed unequivocally how the public’s opinion had several concerns about potentially being allocated to a control group that did not receive screening. A preference for a stepped-wedge design was observed in ex-patients, their relatives, and carers, despite the well-known limitations of this type of trial design [12] and the effect any trial results may have on health policy or service commissioning. It is therefore clear that these aspects of the research programme will require detailed behavioural analysis and process evaluation prior to evaluation of effectiveness in any future trial.

### Strengths and limitations

The Delphi process had several strengths. First, the programme was driven by patients. Aortic Dissection Awareness UK is composed of over 250 aortic dissection survivors and their relatives, who were able to bring their collective experience to the process in partnership with members of the ICC, the clinical infrastructure principally responsible for managing aortopathy in the UK, and a panel of clinical experts in genetic testing, imaging, genetic counselling, and trial design. This increases the likelihood that the concerns and needs of the people that will be ultimately affected by screening are considered in the research programme. Second, following a consultation, we developed a questionnaire that was initially assessed and then refined following a pilot phase that yielded high-quality data. Third, to our knowledge, the final workshop which involved 164 participants is the largest patient and public involvement event in cardiovascular research in the UK. On the day, the workshops were held after a preliminary session of informative talks on the patient experience as well as the likely pathogenesis of the disease. This enhanced the quality of the discussion in the workshops that were led jointly by aortic dissection survivors and clinical experts. Fourth, the process collected separate responses from experts, members of the ICC, and the public. This provided important insights into the differing perspectives that each group brings to the process and provides a pathway to resolving these differences as part of the research programme. Among the limitations of the Delphi process was that it failed to deliver consensus on many issues. The Delphi format is itself inflexible and limits the interpretation of peoples’ responses. The face-to-face workshop, although beneficial for the discussion between patients and clinicians, lacks the methodological advantages of the phase conducted in anonymity. A relatively low cut-off value for consensus [10, 13, 14], already implemented in similar experiences [15, 16], was specifically chosen to account for the mixed composition of the expert panel and to ensure the definition of the necessary characteristics of the research programme was as comprehensive as possible. There are also logistical limits to the number of topics that can be explored. The process was not a research project however, and the results that have been obtained are likely to enhance future efforts by researchers to address residual disagreement where possible or uncertainty through clinical trials. Overall, the process served to highlights the lack of evidence and need for research in the topic explored.

## Conclusions

A Delphi process brought researchers and the public together in a project that addressed an unmet clinical need and could potentially be the starting point to change existing practice [17]. It produced a consensus as well as a list of gaps in knowledge about surveillance pathways in thoracic aortic disease (with a focus on non-syndromic forms and higher risk phenotypes), identified by patients and experts together, in one of the largest patient and public involvement (PPI) initiatives in cardiac surgery research. Their contribution will serve (together with the pilot study currently undergoing at our centre, NCT03861741) to guide the design of a clinical research programme investigating the benefits of targeted screening for thoracic aortic diseases.

## Supplementary information

**Additional file 1.**

**Additional file 2.**

**Additional file 3.**

**Additional file 4.**

**Additional file 5.**

**Additional file 6.**

## Data Availability

All reports generated from the questionnaire and the materials used to run the Delphi are provided as Additional files.
