# Supplementary material for: Report of a Delphi exercise to inform the design of a research programme on screening for thoracic aortic disease
Source: Trials. 2020 Jul 16;21:656. doi: 10.1186/s13063-020-04562-1 (PMC7367380; doi:10.1186/s13063-020-04562-1)
Supplement: Supplementary file 1 — Additional file 1. [file 13063_2020_4562_MOESM1_ESM.pdf]

Methodological characteristics of the Delphi (as per Diamond et al 2014)

**Study objective**	
Does the Delphi study aim to address consensus?	*The Delphi aims to address consensus on screening requirements for thoracic aortic disease but is directly finalised to the design of research project.*
**Participants**	
How will participants be selected or excluded?	*Participants are selected because of their background expertise. Participants' availability to attend a face-toface discussion with patients is a requirement.*
**Consensus definition**	
How will the consensus be defined?	*The consensus is defined on a percentage basis (60%). However, due to the nature of the process itself, some of the questions require a face-to-face discussion.*
If applicable, what threshold value will be required for the Delphi to be stopped based on the achievement of consensus?	*Even in case of consensus during the first two rounds of our modified Delphi exercise, the question would still be presented and discussed during the final face-to-face meeting (workshop session).*
What criteria will be used to determine when to stop the Delphi in the absence of consensus?	*Not applicable.* *[see above]*
**Delphi process**	

Were items dropped?	*No item was dropped.*

Methodological characteristics of the Delphi (as per Diamond et al 2014)

What criteria will be used to determine which items to drop?	*Every item would undergo discussion during the Workshop session.*
What criteria will be used to determine to stop the Delphi process or will the Delphi be run for a specific number of rounds only?	*The Delphi runs for a specific number of rounds (2), plus the additional meeting between patients and clinicians.*

Delphi Process – Extended narrative and rationale of the phases

Preliminary survey

The preliminary survey [fig. 1] was a preparatory exploration of the public opinions and questions regarding the topic of interest, and panellists' role was limited to evaluating the potential documents that were used for the survey.

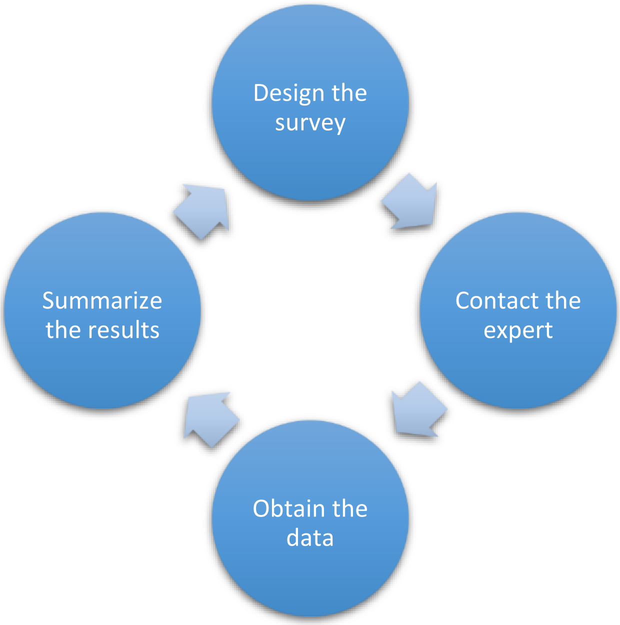

Figure 1 – *Preliminary survey methodology*

A leaflet version and an online survey were presented during the SCTS National Conference (10-12 March 2019) and on AD Awareness UK and Ireland websites and Social Network pages and then circulated in a two months period (from March 2019 to May 2019).

The survey captured interested parties' questions around four main areas of aortic disease research that are especially relevant for screening:

- Imaging
- Genetics
- Counselling
- Screening

Forty-nine respondents took part in the survey, for a total amount of 180 contributions, equally distributed among the four topics.

The list of questions was subsequently filtered and analysed by the Planning Committee to form the layout for the second round. At the end of the process, data were presented to panellists with tag clouds and radar charts, along with the complete unfiltered list of questions from the survey respondents.

Round I

Delphi Process – Extended narrative and rationale of the phases

Round I was run in a two months period (from May 2019 to July 2019); the experts received a series of questions derived from the first survey in form of a digital questionnaire. Based on the nature of the questions itself, the Planning Committee reserved the possibility to use one among the three options exemplified in figure 2. The topics explored included the acceptability of imaging examinations in healthy relatives, the psychological burden of a screening programme, the involvement of minors, the optimal timing to offer counselling to patients and the professional figures that should be involved in the management of this condition.

The objective of Round I was to obtain a set of statements produced by the different panels showing a series of semi-quantitative figures representing their consensus.

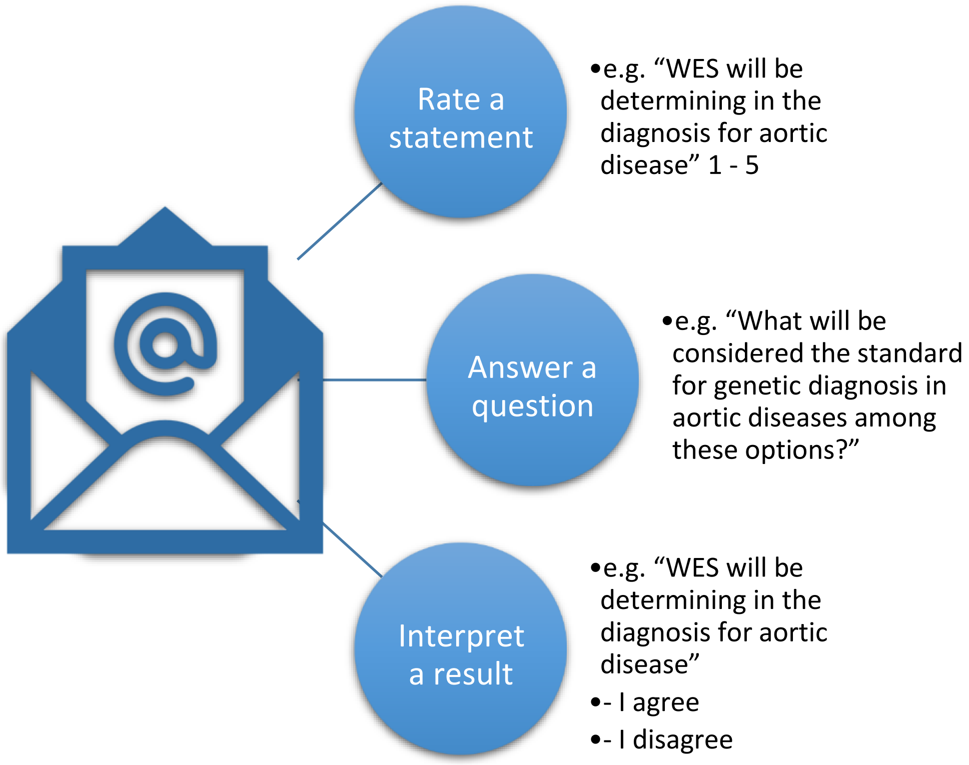

Figure 2 – *Examples of question structure.*

Two parallel initiatives were run contemporarily to Round I, to inform and improve the results of this modified Delphi experience.

1. **ICC Services Survey**

    Based on suggestions offered during the preliminary teleconferences, the Planning committee sent a questionnaire to the regional Inherited Cardiac Conditions (ICC) Services across the UK. The aim was to obtain a description of what is the current standard of care for thoracic aortic disease screening in the Country, identify potential

lack of equipoise, and correctly assess the volume of activity this task represents in the different regions.

2. **Parallel Lay Delphi Questionnaire**
   In order to collect patients' perspectives and experience, a parallel questionnaire was launched at the same time of the main one reserved to the panellists. This lay version of the questionnaire targeted mainly patients and carers and was advertised and distributed digitally in a similar way to the preliminary survey, through the Aortic Dissection Awareness UK & Ireland channels. Topics treated and questions list mirrored the ones present in panellists' questionnaire.

Results from Round I are available in Appendix 5. 28 panellists took part in the first round and 46 responses were obtained for the lay version of the questionnaire.

Round II

Round 2 (fig. 3) was conducted from July 2019 to September 2019. Panellists received a full report of the intermediate results from the lay version of the questionnaire, and from the first round of the version they had replied to in the previous months.

Participants were then asked to use a revised digital version of the survey to state their opinion after reading the report. Disagreeing with the current consensus required to add a motivation to the answer provided. Exploring disagreement prevented excessive dissenters drop out and generation of an artificial consensus.

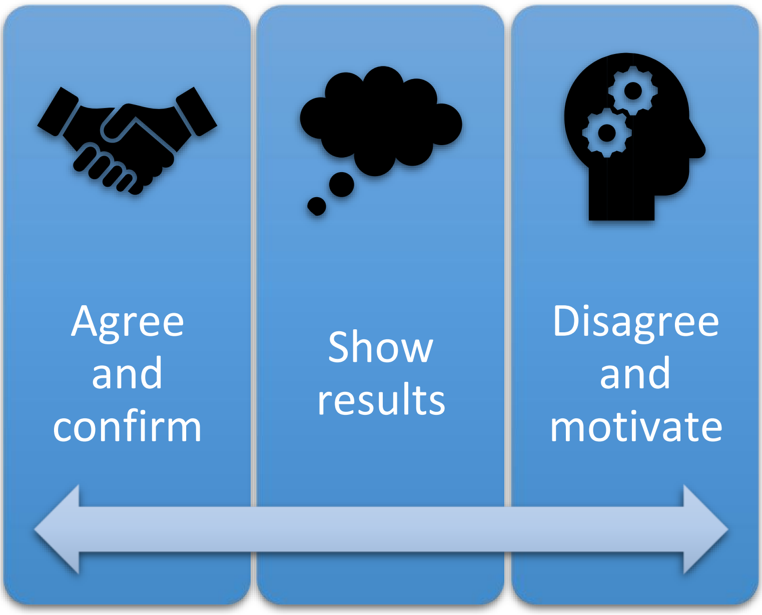

Figure 3 – *Approach for the second Round of the Delphi.*

Delphi Process –
The ICC Services Survey and the lay version of the questionnaire were still collecting responses for the whole duration of Round II.

At the end of Round II, 21 responses were provided to the panellists' survey, and 84 to the lay questionnaire. 6 ICC Services replied to the letter sent.

Round III

During the Aortic Dissection Awareness Day UK 2019 (attended by approximately 120 patients and 40 professionals; the public was able to interact with the panellists directly during a dedicated Workshop session; every discussion group was led by a clinician and a lay member of the national patient association.

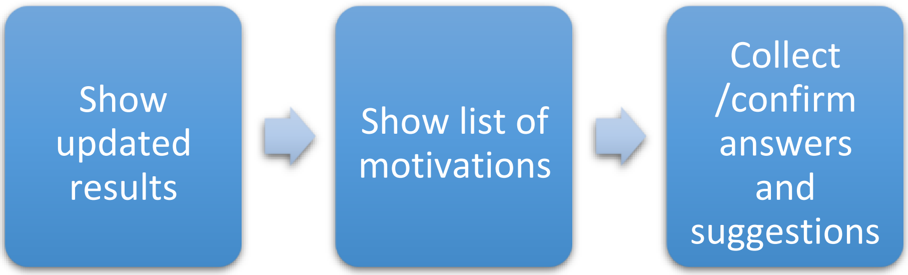

Figure 4 – *Rationale for the third Round of the Delphi.*

Patients and experts opinion was guided both by the semi-quantitative results of the previous rounds that were shown at Conference venue and by the talks given by the invited speakers, focusing on themes relevant to a screening programme for aortic diseases.

The session was followed by the presentation of the results that this collaboration had reached at the end of the day.

The workshop session granted an additional input from patients to the described Delphi methodology, in addition to the one deriving from the set of questions identified during the first round.

Process results

At the end of the conference, results from the process were presented to the attendants. Consensus, if reached, was not necessarily modified by further discussion, however the face-to-face meeting gave the participants involved an opportunity to ask questions and raise concerns that were taken into consideration when disseminating the output from the conference and from the Delphi.

Each individual workshop session results is summarised below.

Imaging Workshop

Main points from the discussion:

1. Diagnosis of a syndromic form should not prevent further screening
2. Screening should be linked with genetics but also to other package of aftercare
3. Discuss resource repercussion of one-off or serial screening
4. Psychological explanations should be implemented
5. Choice of operation
6. No marked difference can be recognised in costs, however access and availability related issues may be relevant
7. Produce a timelines of results
8. Signs of Marfan's Syndrome should be carefully searched and investigated
9. Focused screening only
10. Implementation of A.I. possibilities is an exciting option but still unexplored in clinical setting.
11. Links to genetic testing and age will allow for individualisation, based on sequencing results, family history and patient choice.
12. Screening needs to be followed by surveillance when adequate, and planned as a wider package of support from the beginning
13. Cost/disability

Molecular Genetics Workshop

Molecular Genetics workshop discussion focused on current standard of care. An interesting remark highlighted during the discussion was the opportunity to adopt a similar pathway to the one already in place for sudden cardiac death screening.

Clinical Genetics Workshop

Potential Stakeholders: Cardiology ICC, Patients, Relatives, Clinical Genetics

1. Consider any affected person with a TAAD as potentially having an inherited disease and refer to an ICC. Also consider consenting for storing intimate tissue at surgery.
2. Store DNA with next of kin consent in acute setting. Genetic testing of at risk relatives without disease and where no gene change has been identified in the index case is not yet advised as result interpretation in this context is not yet good enough due to uncertainty about detecting missense variants
3. Use a proforma to discuss a referral to a family history clinic on a discharge proforma if not mentioned before
4. Ensure ICC access universally in the UK and treat TAAD very similarly to other sudden death susceptibility conditions

5. Ensure that somebody with experience talking about relatives and family history is available as the first post diagnosis outpatient appointment to discuss family cascading of imaging and the test directory
6. Offer first degree relative MRI as first line. If disease in a second relative (in other words the index patient and one more) is identified, consider genetic testing regardless of age
7. If recommended on test directory consider offering a gene panel test at this point
8. If the phenotype matches a clearly pathogenic phenotype then it is safe to discharge those with a negative predictive test
9. Offer second degree unaffected relative screening if an affected family member has a positive family history even if there is no clear mutation in the family even if the intervening relative is unaffected-research identified
10. Teams should consider wider psychological implications of disease on patient and partner- research identified
11. Holistic care teams should be established to tackle other medical sequelae - research identified
12. Please consider intimate studies and other medical and environmental risk factors in deciding management and these should be included in a national database to assist agnostic data analysis

<u>Trial Design Workshop</u>

The most relevant element of discussion during the Trial Design workshop was the acceptability by clinicians and family of potential randomisation of participants to a control group that would not receive familiar screening. Suggestions were raised to adopt a trial design (like stepped-wedge cluster randomised controlled trial that would mitigate this issue, by adopting a bundle of shared screening policies gradually in different Centres and then compare the results with the ones obtained during the periods that preceded the introduction of such measures. This trial would also allow all centres to conduct a homogeneous screening programme by the end of the trial.
