# Supplementary material for: Report of a Delphi exercise to inform the design of a research programme on screening for thoracic aortic disease
Source: Trials. 2020 Jul 16;21:656. doi: 10.1186/s13063-020-04562-1 (PMC7367380; doi:10.1186/s13063-020-04562-1)
Supplement: Supplementary file 2 — Additional file 2. [file 13063_2020_4562_MOESM2_ESM.pdf]

Appendix 1

Do I need NHS REC approval?

AD Awareness Day 2019 Delphi

Your answers to the following questions indicate that **you do not need NHS REC approval for sites in England.** However, **you may need other approvals.**

You have answered ***'YES'*** to: Is your study research?

You answered ***'NO'*** to all of these questions:

<u>Question Set 1</u>

Is your study a clinical trial of an investigational medicinal product?<br><br>Is your study one or more of the following: A non-CE marked medical device, or a device which has been modified or is being used outside of its CE mark intended purpose, and the study is conducted by or with the support of the manufacturer or another commercial company (including university spin-out company) to provide data for CE marking purposes?<br><br>Does your study involve exposure to any ionising radiation?<br><br>Does your study involve the processing of disclosable protected information on the Register of the Human Fertilisation and Embryology Authority by researchers, without consent?	**NO**

<u>Question Set 2</u>

Will your study involve potential research participants identified in the context of, or in connection with, their past or present use of services (adult and children's healthcare within the NHS and adult social care), including participants recruited through these services as healthy controls?<br><br>Will your research involve collection of tissue or information from any users of these services (adult and children's healthcare within the NHS and adult social care)? This may include users who have died within the last 100 years.<br><br>Will your research involve the use of previously collected tissue or information from which the research team could identify individual past or present users of these services (adult and children's healthcare within the NHS and adult social care), either directly from that tissue or information, or from its combination with other tissue or information likely to come into their possession?	**NO**

Will your research involve potential research participants identified because of their status as relatives or carers of past or present users of these services (adult and children's healthcare within the NHS and adult social care)?

Question Set 3

Will your research involve the storage of relevant material from the living or deceased on premises in the UK, but not Scotland, without an appropriate licence from the Human Tissue Authority (HTA)? This includes storage of imported material.

Will your research involve storage or use of relevant material from the living, collected on or after 1st September 2006, and the research is not within the terms of consent from the donors, and the research does not come under another NHS REC approval? **NO**

Will your research involve the analysis of DNA from bodily material, collected on or after 1st September 2006, and this analysis is not within the terms of consent for research from the donor? And/or: Will your research involve the analysis of DNA from materials that do not contain cells (for example: serum or processed bodily fluids such as plasma and semen) and this analysis is not within the terms of consent for research from the donor?

Question Set 4

Will your research involve at any stage intrusive procedures with adults who lack capacity to consent for themselves, including participants retained in study following the loss of capacity?

Is your research health-related and involving prisoners? **NO**
Does your research involve xenotransplantation?
Is your research a social care project funded by the Department of Health and Social Care (England)?

Aortic Dissection Awareness Day UK 2019 - Working Group			
**IMAGING**	**GENETIC TESTING**	**CLINICAL GENETICS**	**TRIAL DESIGN**
Chair: Graham Cooper	Chair: Matthew Bown	Chair: Julian Barwell	Chair: Rob Sayers
Co-Chair: Catherine Fowler	Co-Chair: Dianna Milewicz	Co-Chair: Karen English	Co-Chair: Gareth Owens
Mark Callaway	Huw Dorkins	Noelle Robertson	Michael Sweeting
Rajesh Chelliah	Mark Field	Anne Cotton	Cassandra Brookes
Aparna Deshpande	Catherine Fletcher-Francis	Sarah Gunn	Tracy Kumar
Jeffrey Khoo	Cliff Grover	Victoria McKay	Florence Lai
Gerry McCann	Giovanni Mariscalco	Aung Oo	Gavin Murphy
Praveen Rao	Toru Suzuki	Deborah Osio	
	Amy Yasbeck	Nora Shannon	
		Saba Sharif	
		Nigel Wheeldon	
