# Supplementary material for: Report of a Delphi exercise to inform the design of a research programme on screening for thoracic aortic disease
Source: Trials. 2020 Jul 16;21:656. doi: 10.1186/s13063-020-04562-1 (PMC7367380; doi:10.1186/s13063-020-04562-1)
Supplement: Supplementary file 3 — Additional file 3. [file 13063_2020_4562_MOESM3_ESM.pdf]

Surveillance for Thoracic Aortic Diseases

Aortic Dissection Awareness Day 2019

Dear Colleague,

Aortic Dissection Awareness UK, in partnership with a panel of clinical experts in aortic disease, is conducting a Delphi process (with full patient involvement) to explore current practice and possible future perspectives on surveillance for thoracic aortic diseases (with a focus on non-syndromic forms).

Our professional network has multidisciplinary expertise (imaging, genetics, genomic medicine, and trial design).
The Delphi process seeks to establish the requirements of a desirable screening programme, and to identify the best way to implement and to evaluate the effectiveness of their adoption. An important step in this process is determining what the standard of care is and who delivers it at the moment, hence our request for your kind collaboration with the following questions.

1. **1. Which ICC Service do you work for?**

   ____

2. **2. How many patients affected by aortopathy does your Centre sees approximately each year?**

   ____

3. **3. What percentage of your workload does this number constitute?**

   ____

4. **4. What would you say is the current pathway for patients affected by thoracic aortic disease (non-syndromic) in your region? Please, feel free to specify any variation or any additional factors, and to give us any comment or suggestions you may have. To assist you we have drafted some specimen pathways below.**

   ____
   ____
   ____
   ____
   ____

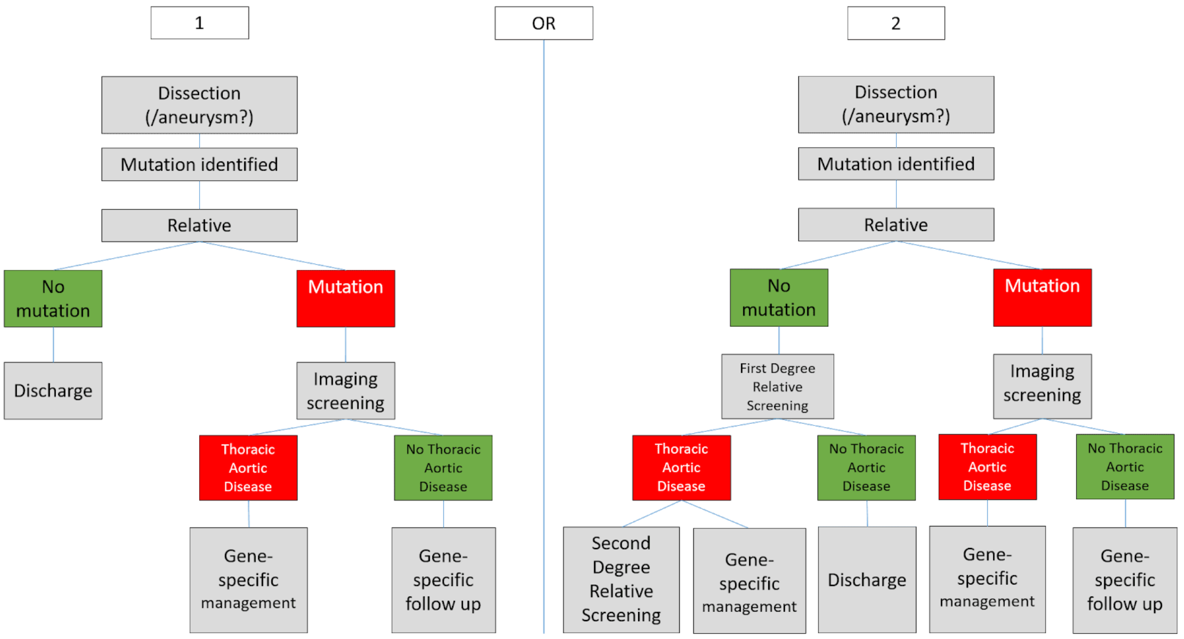

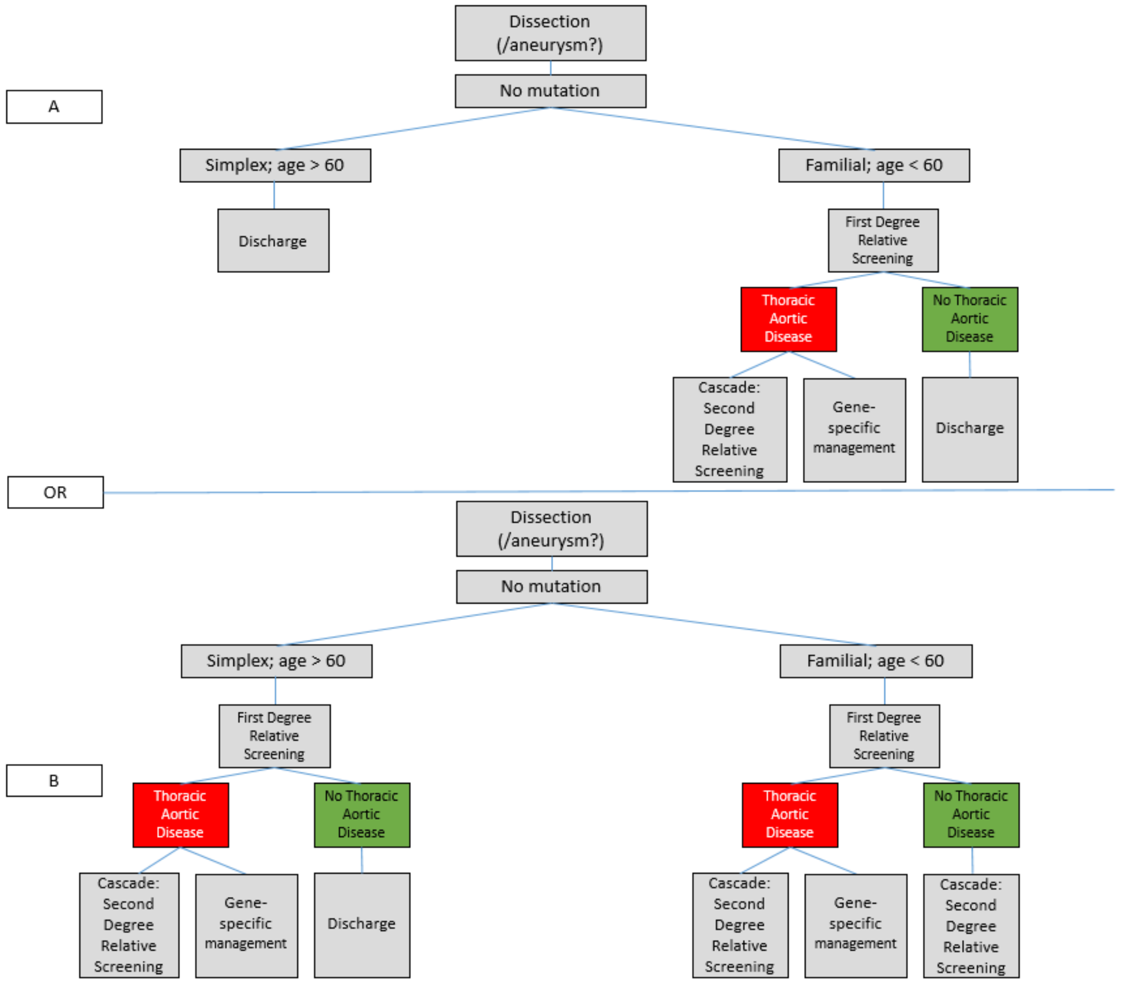

5. **5. In your opinion, should genetic and imaging approach be combined, or should one follow the other? How do patients' characteristics (genotype, phenotype, age, personal preferences) influence your choices in terms of timing of the different tests?**

6. **6. Based on your experience, what are the main differences in clinical genetics pathway (and in patient support needs) between this condition and the others managed in the ICCs (e.g. SADS).**

7. **7. Should a blood sample from a patient with aortic dissection be collected routinely before an urgent operation, for the purpose of genetic testing?**

We are extremely grateful for any answer you may provide and we will be glad to update you with the results of this process if you are interested; moreover, we will make sure you receive the outcome of the discussion we will have on the Conference day.
