# Supplementary material for: Report of a Delphi exercise to inform the design of a research programme on screening for thoracic aortic disease
Source: Trials. 2020 Jul 16;21:656. doi: 10.1186/s13063-020-04562-1 (PMC7367380; doi:10.1186/s13063-020-04562-1)
Supplement: Supplementary file 4 — Additional file 4. [file 13063_2020_4562_MOESM4_ESM.pdf]

Aortic Dissection Awareness Day 2019

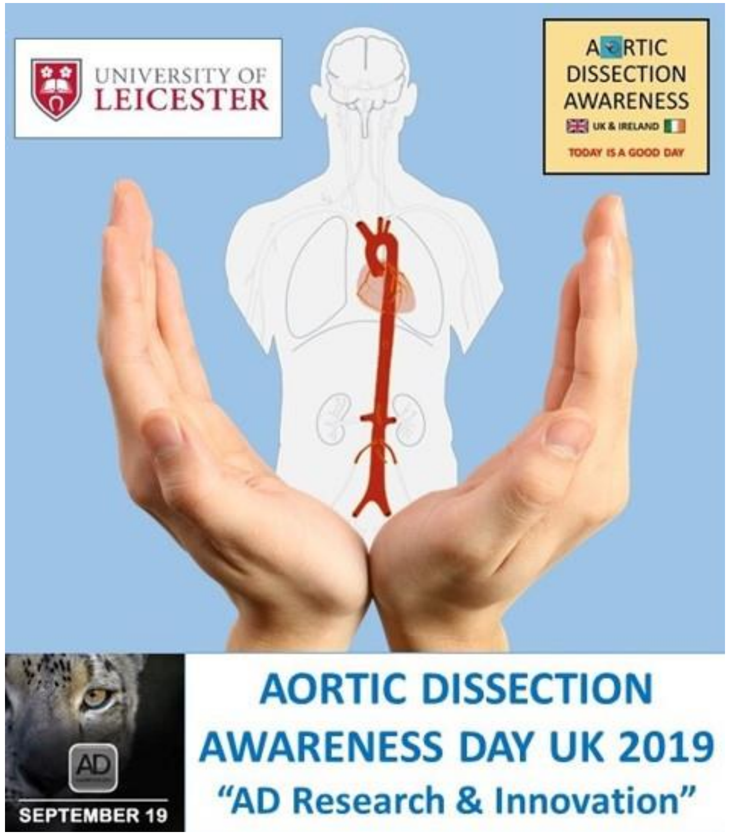

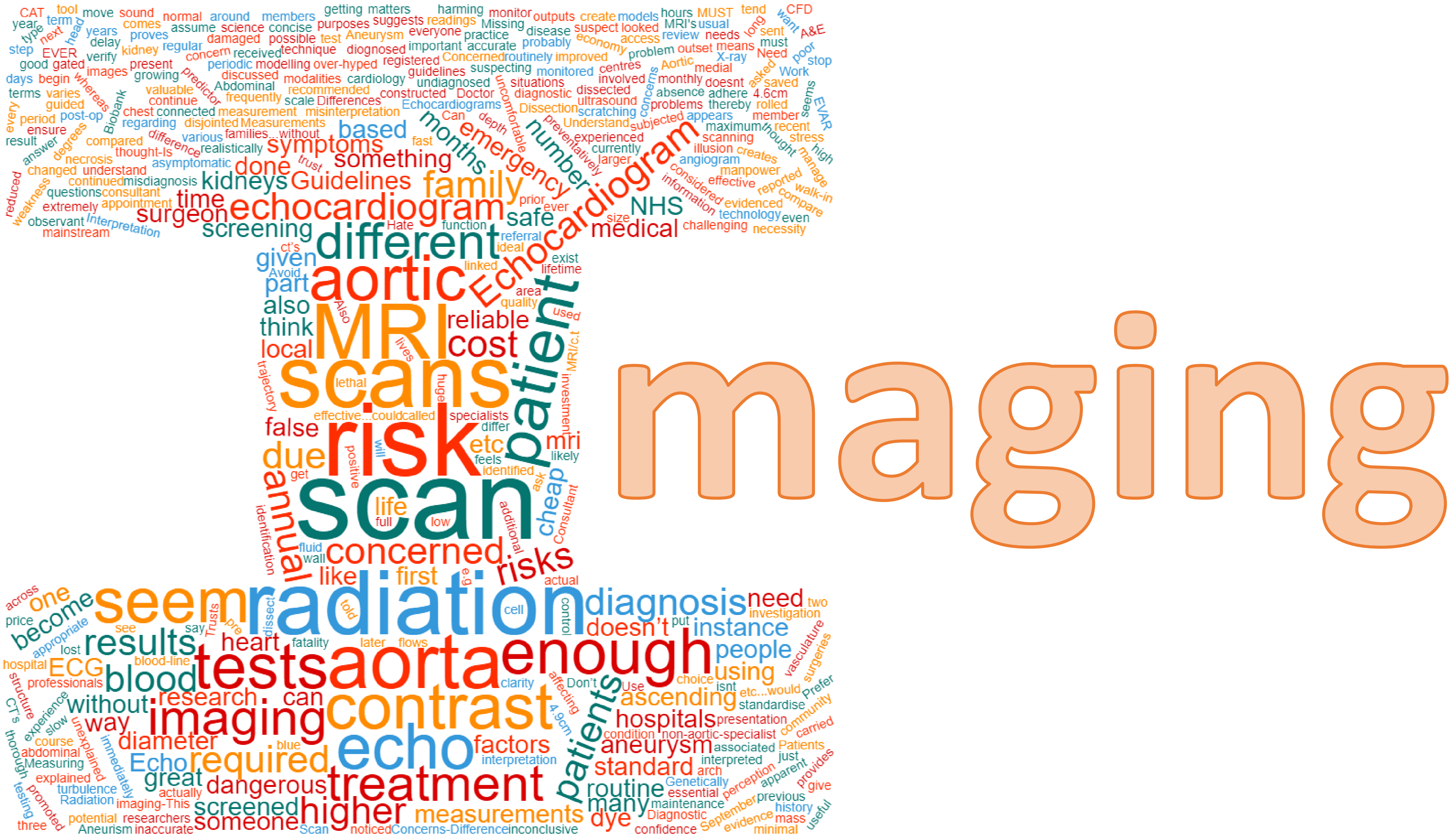
maging
scan
risk
MRI
scans
aortic
different
patient
radiation
aorta
tests
enough
contrast
echo
imaging
treatment
higher
seem
results
blood
diagnosis
echocardiogram
Echocardiogram
concerned
risks
required
patients
cost
reliable
annual
due
family
something
symptoms
Guidelines
kidneys
screening
surgeon
time
emergency
months
number
NHS
medical
safe
based
done
given
part
also
think
local
false
life
cheap
mri
etc
first
like
need
doesn't
instance
people
using
ascending
hospitals
aneurysm
standard
routine
many
dye
factors
measurements
dangerous
screened
someone
great
Echo
diameter
way
research
can
heart
without
ECG
become
one
same

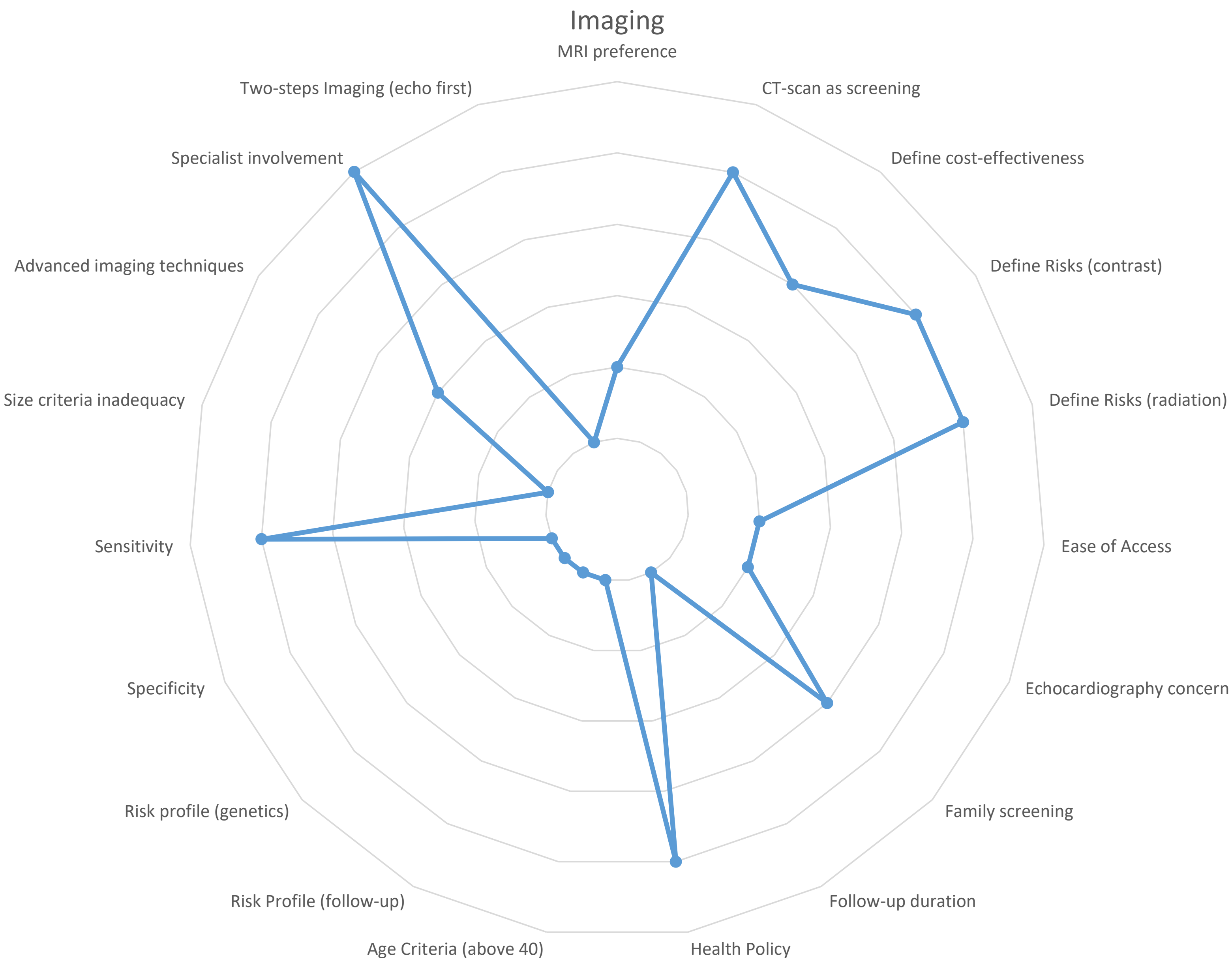

MRI preference	2
CT-scan as screening	5
Define cost-effectiveness	4
Define Risks (contrast)	5
Define Risks (radiation)	5
Ease of Access	2
Echocardiography concern	2
Family screening	4
Follow-up duration	1
Health Policy	5
Age Criteria (above 40)	1
Risk Profile (follow-up)	1
Risk profile (genetics)	1
Specificity	1
Sensitivity	5
Size criteria inadequacy	1
Advanced imaging techniques	3
Specialist involvement	6
Two-steps Imaging (echo first)	1

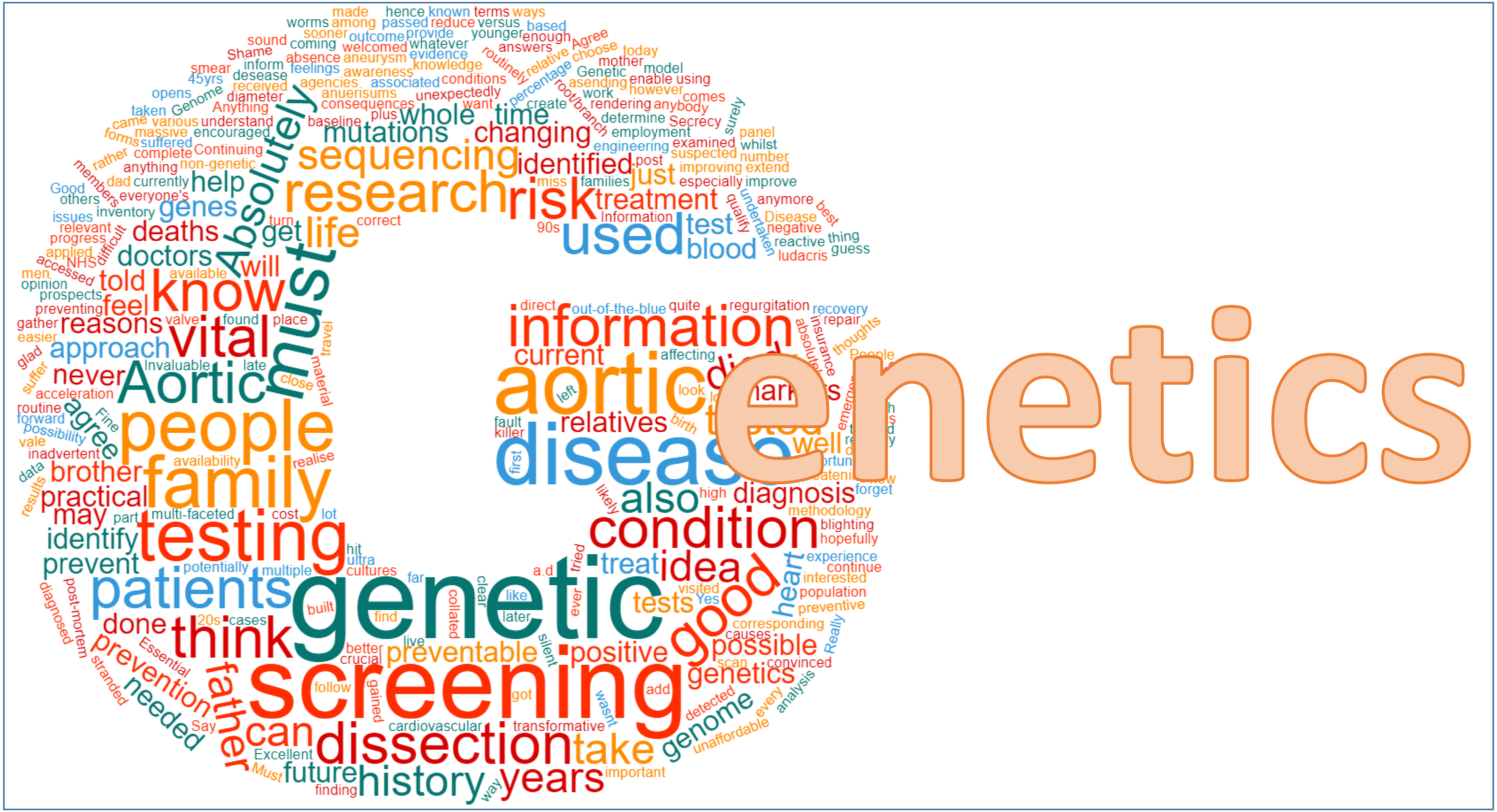
Genetics
genetic
screening
testing
people
family
disease
aortic
Aortic
information
research
risk
must
Absolutely
know
vital
sequencing
condition
dissection
think
patients
good
history
years
take
father
prevention
needed
can
future
preventable
positive
possible
genetics
genome
diagnosis
also
idea
treat
tests
heart
relatives
used
test
blood
treatment
identified
changing
mutations
whole
time
just
life
get
will
deaths
doctors
genes
help
told
feel
reasons
approach
never
agree
brother
practical
may
identify
prevent
done
current
well

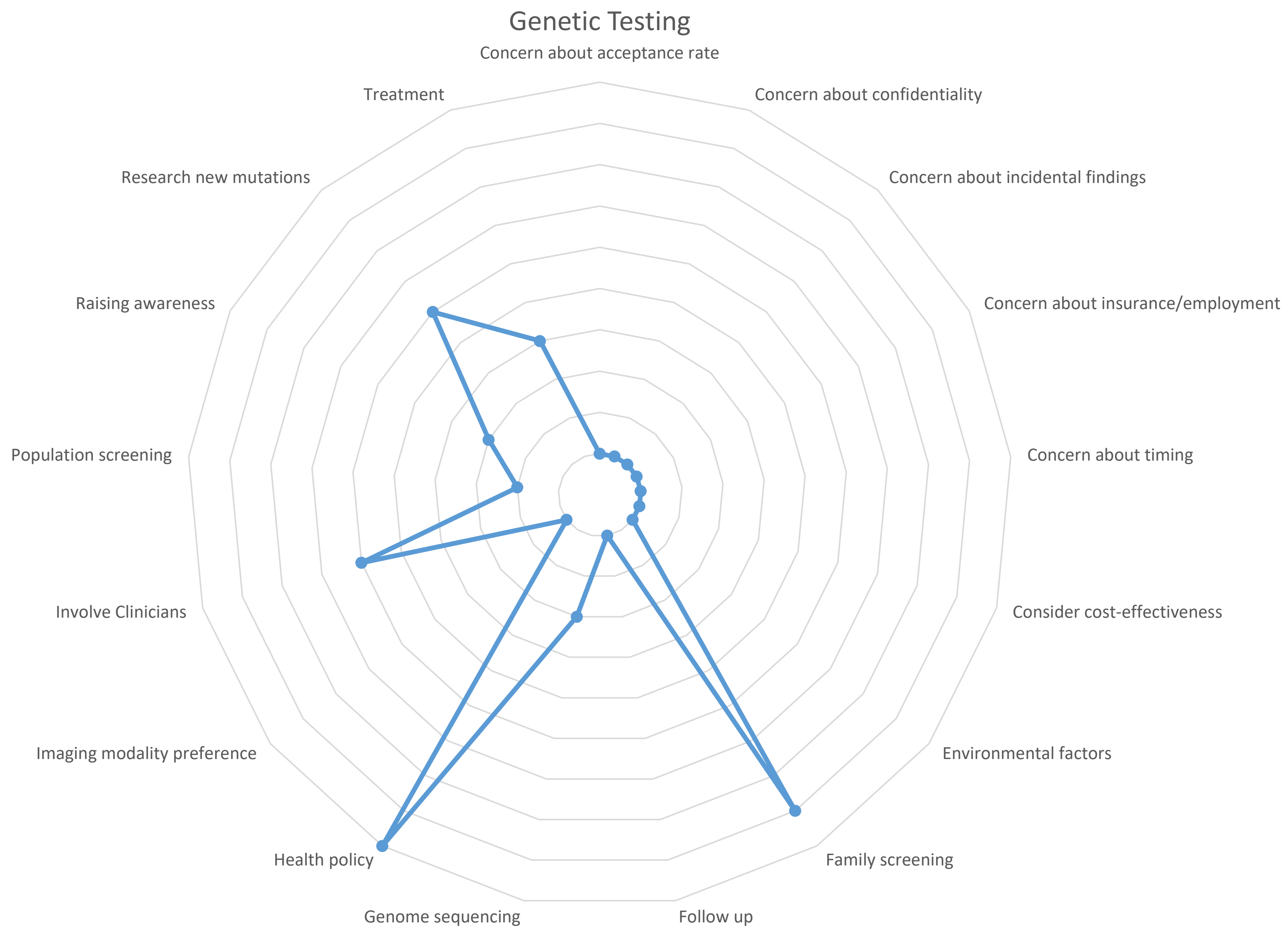

Concern about acceptance rate	1
Concern about confidentiality	1
Concern about incidental findings	1
Concern about insurance/employment	1
Concern about timing	1
Consider cost-effectiveness	1
Environmental factors	1
Family screening	9
Follow up	1
Genome sequencing	3
Health policy	10
Imaging modality preference	1
Involve Clinicians	6
Population screening	2
Raising awareness	3
Research new mutations	6
Treatment	4

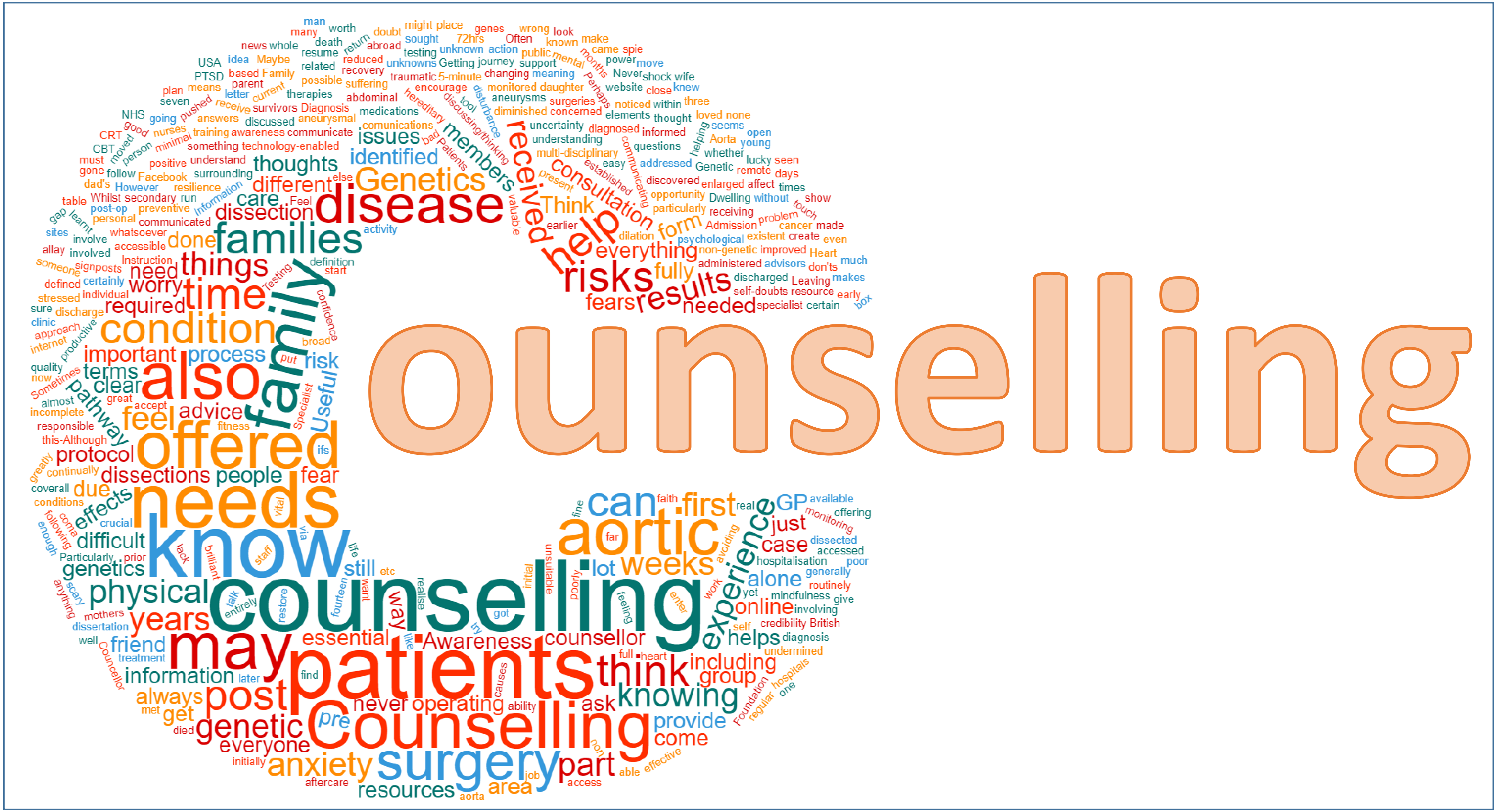
Counselling
counselling
Counselling
patients
know
needs
offered
family
disease
may
also
families
aortic
surgery
think
condition
time
risks
results
help
things
post
genetic
experience
knowing
anxiety
physical
years
weeks
Genetics
received
consultation
members
identified
thoughts
different
dissection
issues
care
worry
need
required
important
process
risk
terms
clear
feel
advice
protocol
dissections
people
fear
effects
difficult
genetics
friend
information
always
get
everyone
resources
area
part
come
provide
group
including
helps
counsellor
Awareness
essential
never
operating
ask
online
alone
experience
case
just
GP
first
can
lot
still
Useful
pathway
done
everything
fully
form
Think
needed
fears

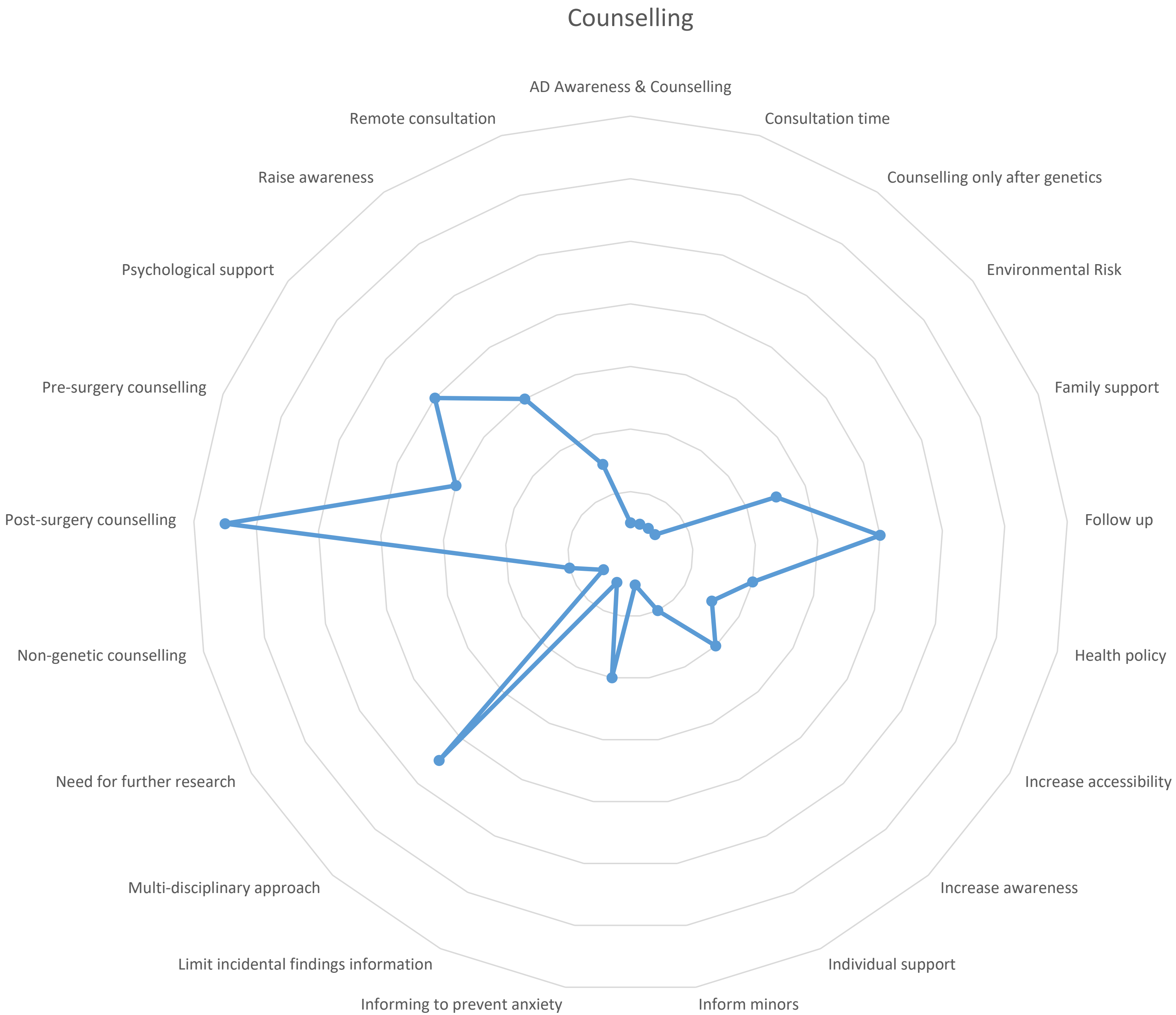

AD Awareness & Counselling	1
Consultation time	1
Counselling only after genetics	1
Environmental Risk	1
Family support	5
Follow up	8
Health policy	4
Increase accessibility	3
Increase awareness	4
Individual support	2
Inform minors	1
Informing to prevent anxiety	4
Limit incidental findings information	1
Multi-disciplinary approach	9
Need for further research	1
Non-genetic counselling	2
Post-surgery counselling	13
Pre-surgery counselling	6
Psychological support	8
Raise awareness	6
Remote consultation	3

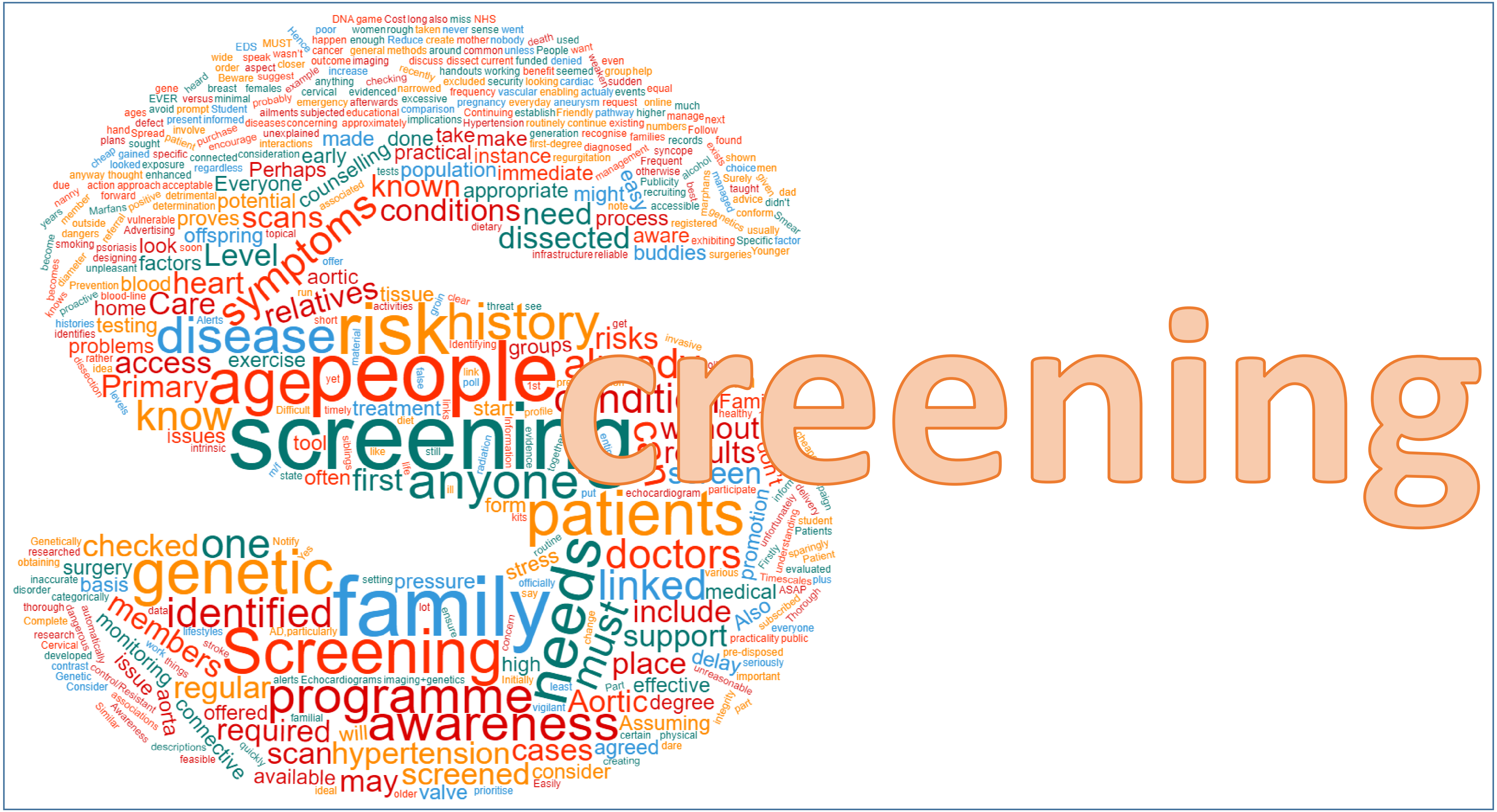

creening
screening
people
family
Screening
risk
disease
age
genetic
programme
awareness
patients
needs
history
symptoms
anyone
know
conditions
known
scans
dissected
need
relatives
heart
Care
Primary
access
identified
members
monitoring
hypertension
screened
cases
doctors
linked
include
support
place
must
checked
one
regular
required
scan
available
may
Aortic
effective
degree
Assuming
agreed
consider
valve
counselling
population
immediate
appropriate
potential
proves
offspring
Level
factors
blood
tissue
testing
problems
exercise
treatment
start
first
often
issues
tool
form
stress
pressure
medical
delay
high
Also
promotion
connective
aorta
issue
offered
will
surgery
basis
Perhaps
Everyone
early
made
done
take
make
practical
instance
process
aware
buddies
might
easy
groups
risks

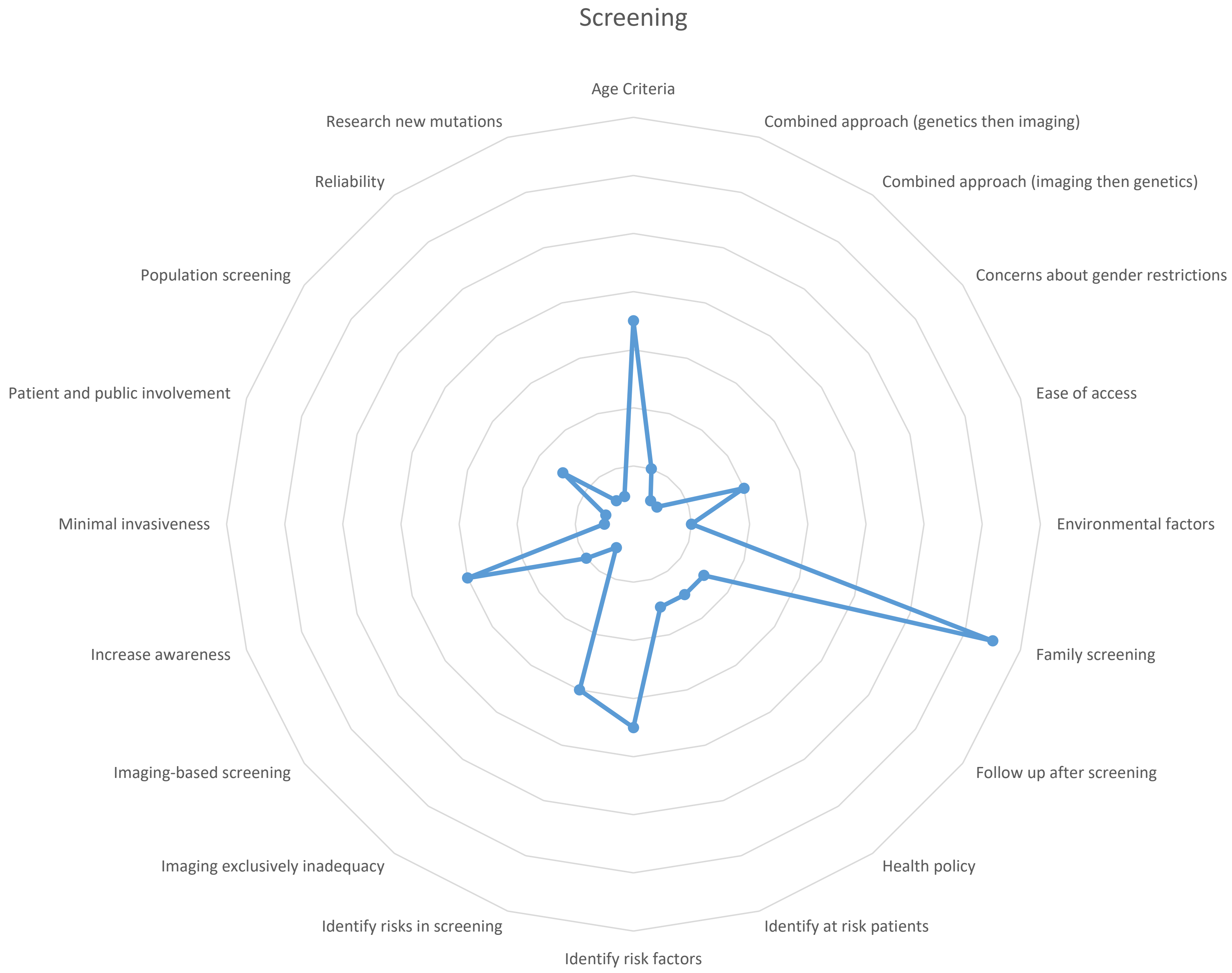

Age Criteria	7
Combined approach (genetics then imaging)	2
Combined approach (imaging then genetics)	1
Concerns about gender restrictions	1
Ease of access	4
Environmental factors	2
Family screening	13
Follow up after screening	3
Health policy	3
Identify at risk patients	3
Identify risk factors	7
Identify risks in screening	6
Imaging exclusively inadequacy	1
Imaging-based screening	2
Increase awareness	6
Minimal invasiveness	1
Patient and public involvement	1
Population screening	3
Reliability	1
Research new mutations	1
