# Supplementary material for: Report of a Delphi exercise to inform the design of a research programme on screening for thoracic aortic disease
Source: Trials. 2020 Jul 16;21:656. doi: 10.1186/s13063-020-04562-1 (PMC7367380; doi:10.1186/s13063-020-04562-1)

AORTIC DISSECTION AWARENESS DAY UK 2019 - DELPHI

Intermediate report

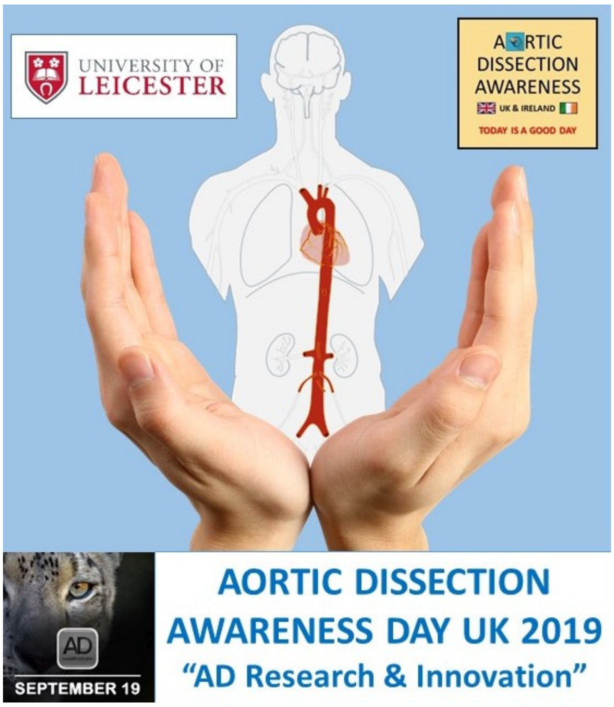

AUGUST 19, 2019

PANELLIST VERSION

Which working group are you involved in?

26 responses

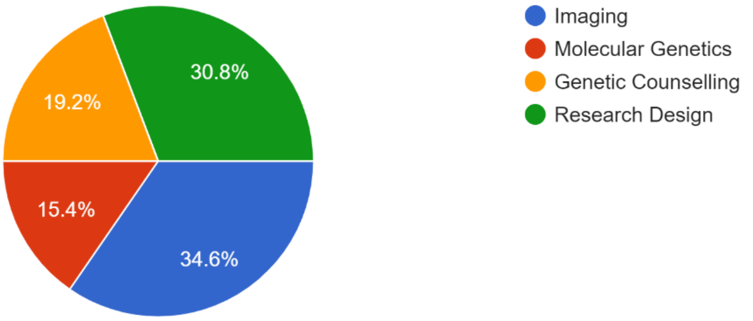

Do you think relatives of patients affected by non-syndromic aortic disease should undergo an imaging test?

25 responses

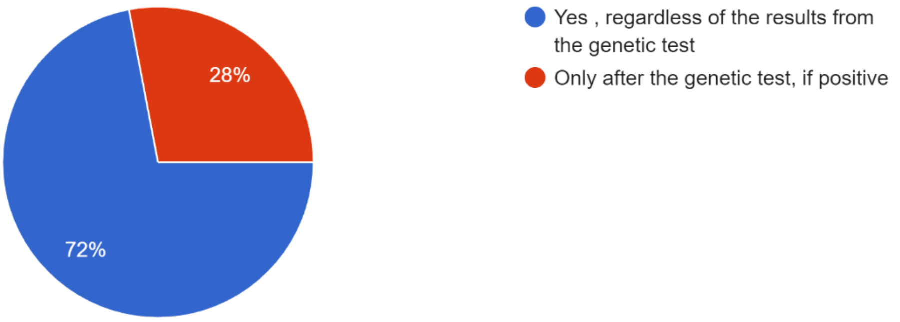

Which imaging test would you consider appropriate, in cases in which no clear genetic condition can be identified?

25 responses

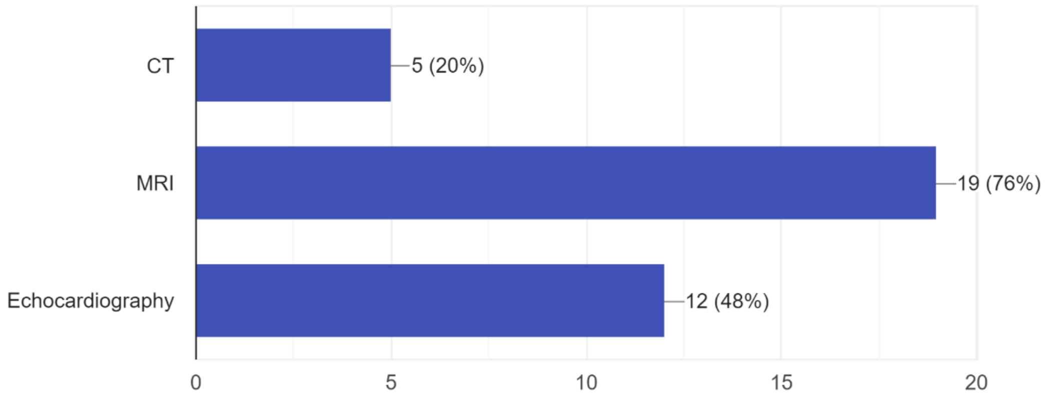

Which imaging test would you consider appropriate, in cases in which a genetic condition can be identified?

25 responses

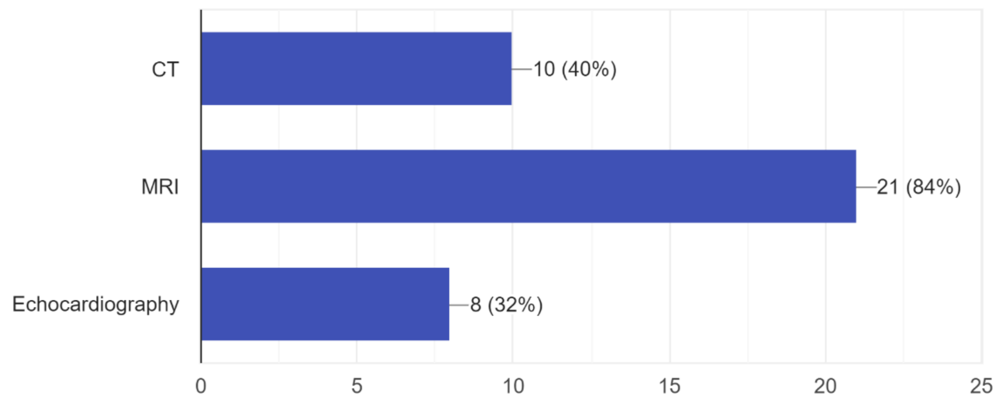

What should be the method of choice for Follow Up in Relatives with an uncertain genetic variant?

25 responses

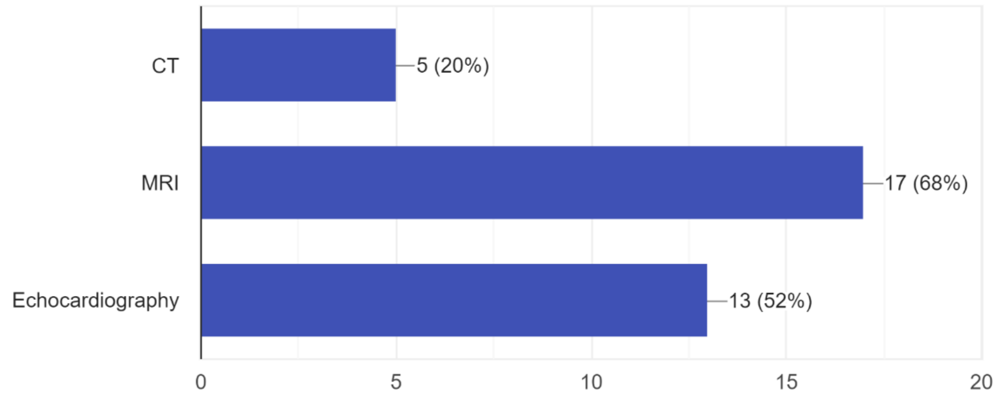

Starting from what age should relatives be screened with an imaging test?

18
10-20 years before the same age as the affected relative
Dependent on presenting age of proband - if as adult > say 30, then 16 appropriate. If proband younger, then screening children may be appropriate.
30
35
Any age
16 years old.
Depends on gene but usually 18
18
At index event
18
Depends on which gene is altered and what the alteration is

depends on family and age of affected but earlier rather than later. if adult onset at least from 20s. if noit sure earlier. if a particular gene this and pts clinicnal status will influence screening interval too.
18
18
16
18
18+
11 years
5 years under youngest age of diagnosis in family
18
Dependent on age of index case and aneurysm/dissection morphology

What should be the optimal follow-up rate in each of these cases?

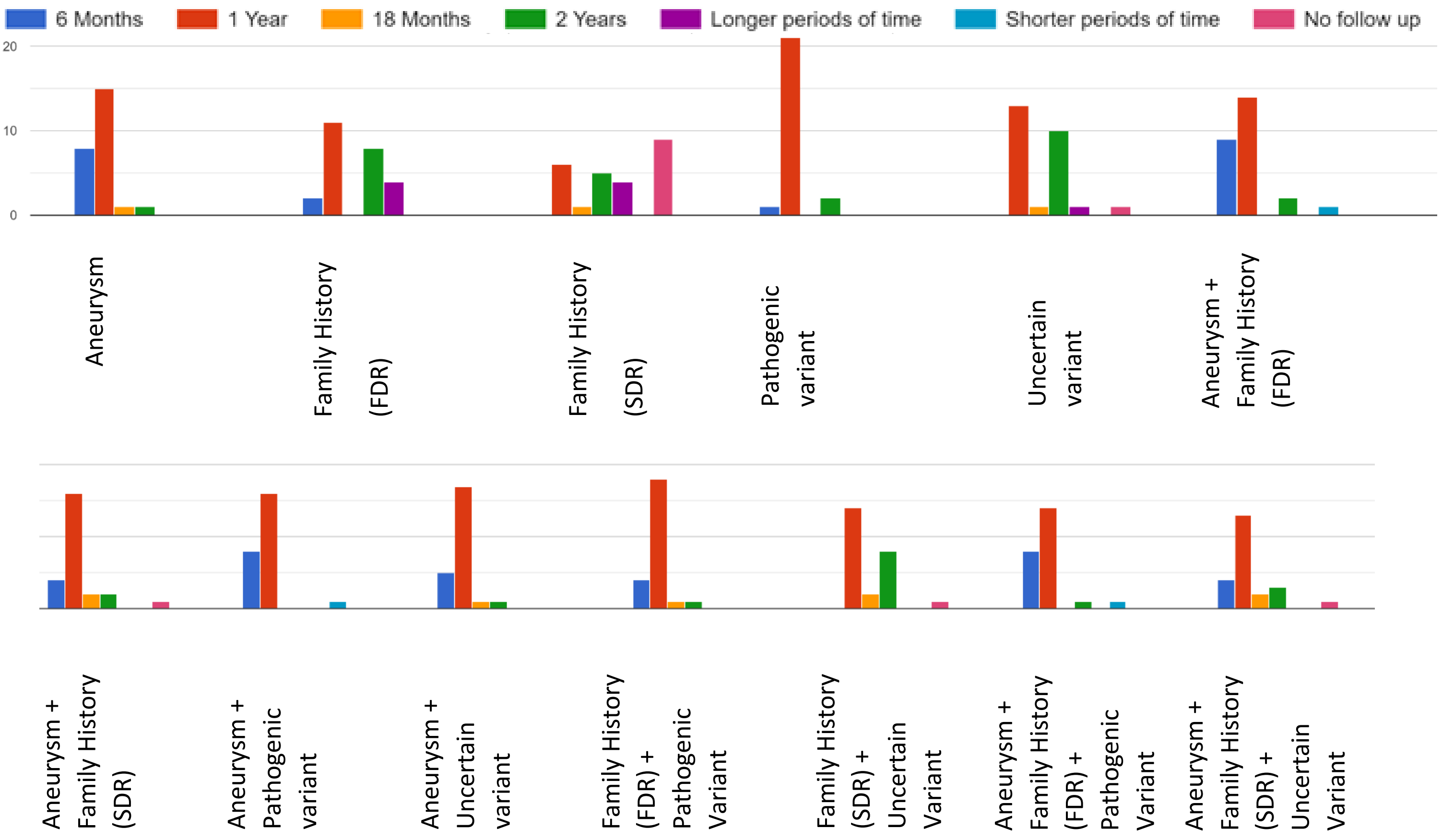

Should incidental findings be a reason to adopt a more focused test?

23 responses

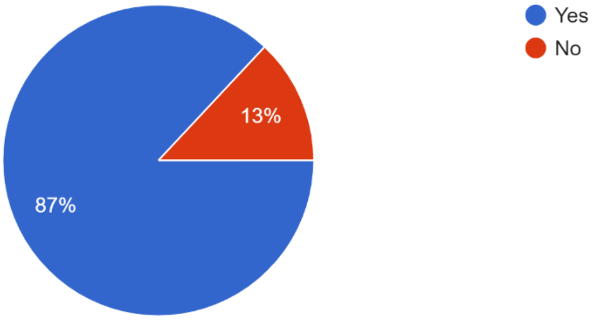

Who should be involved in Genetic Screening? (select all relevant)

24 responses

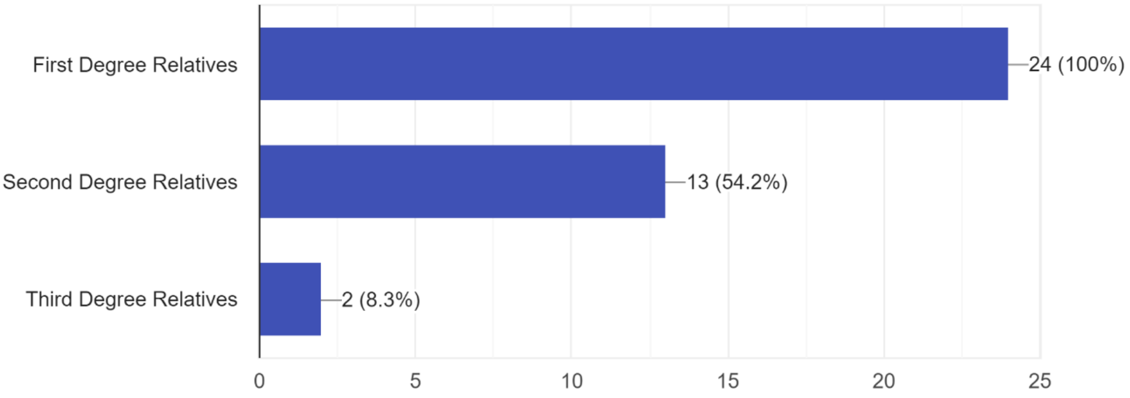

A patient (with a previous negative or inconclusive genetic test result) would require re-testing when/if...

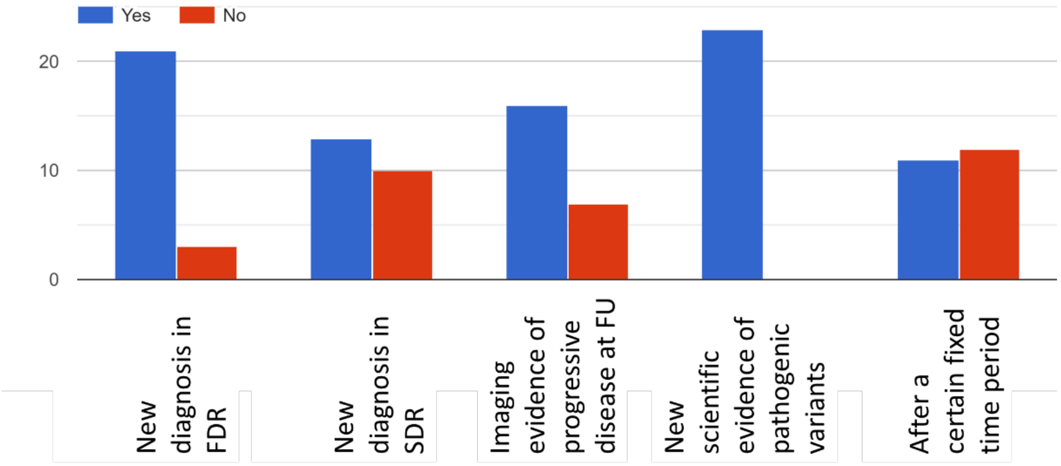

Is it appropriate to store a sample from a patient affected by aortic dissection in any case during an urgent...n, for the purpose of genetic testing?

24 responses

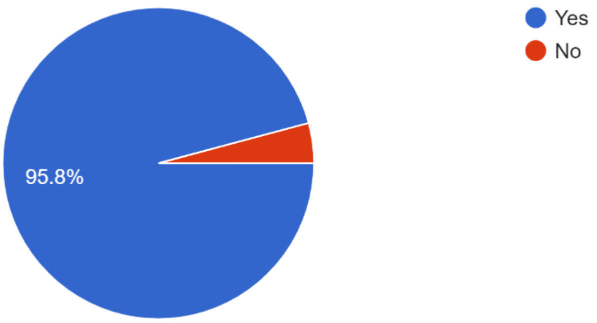

Is it appropriate to discuss genetic testing with the family after an urgent surgery for aortic dissection?

24 responses

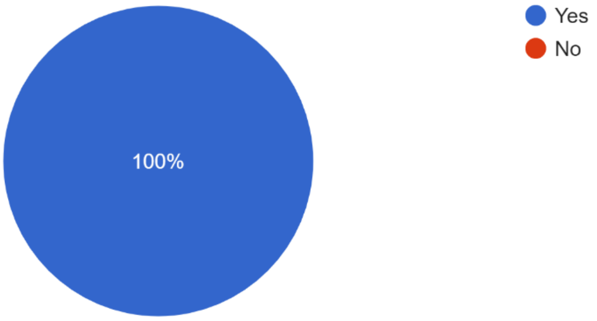

Is it appropriate to discuss genetic testing with the family after a patient dies from aortic dissection?

24 responses

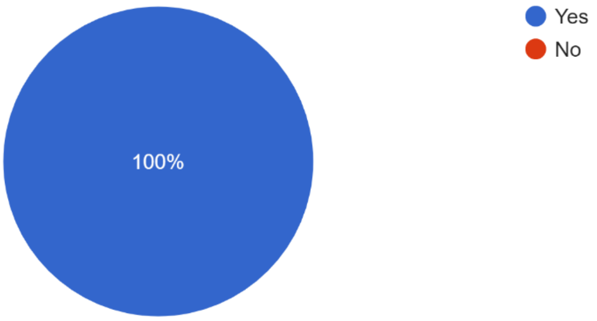

Who should be the professional figure involved in informing patients about genetic risk (and therefore referring them to a clinical geneticist)?

23 responses

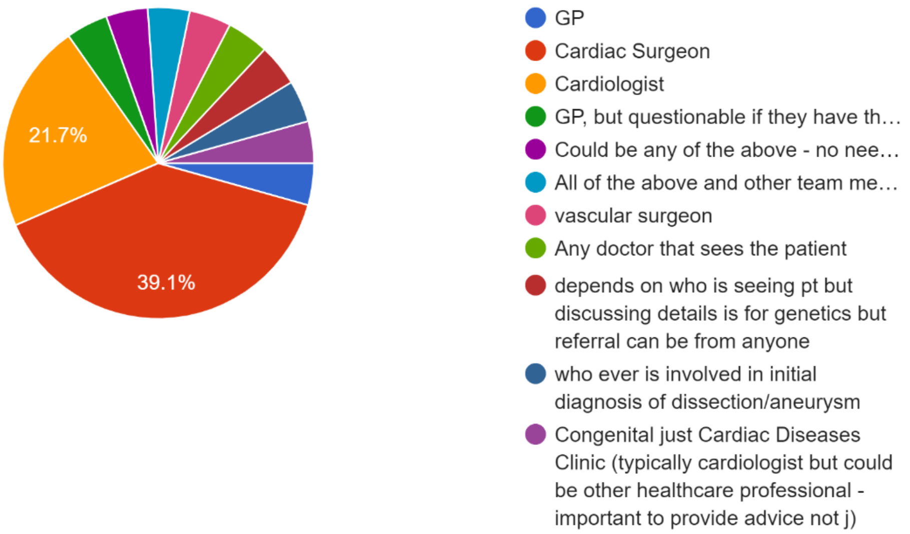

Should a Multidisciplinary Team be involved in the management of these families? What professional figures should be involved from the outset?

24 responses

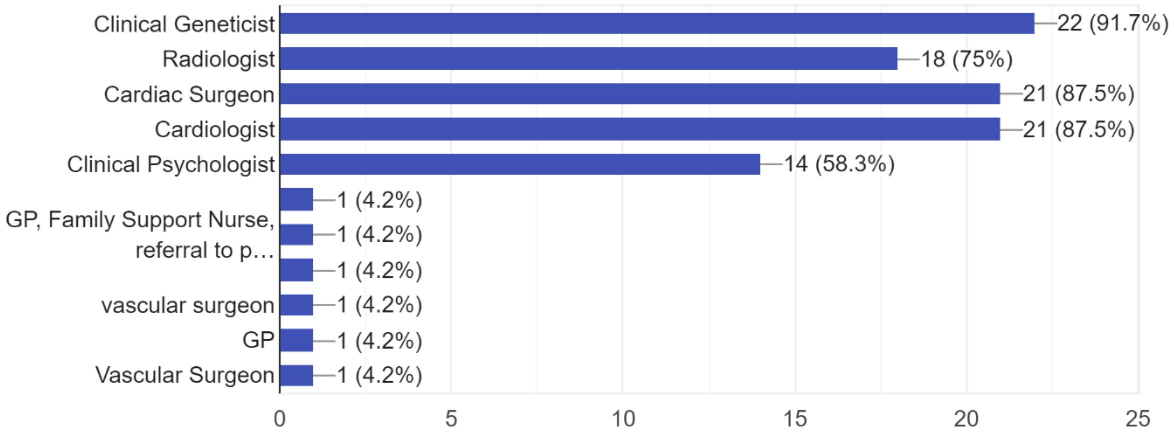

Current scientific evidence shows how each genetic mutation may be associated with a peak in the risk of dissection at a certain age. How many years before the youngest person dissects for that gene should we start surveillance?

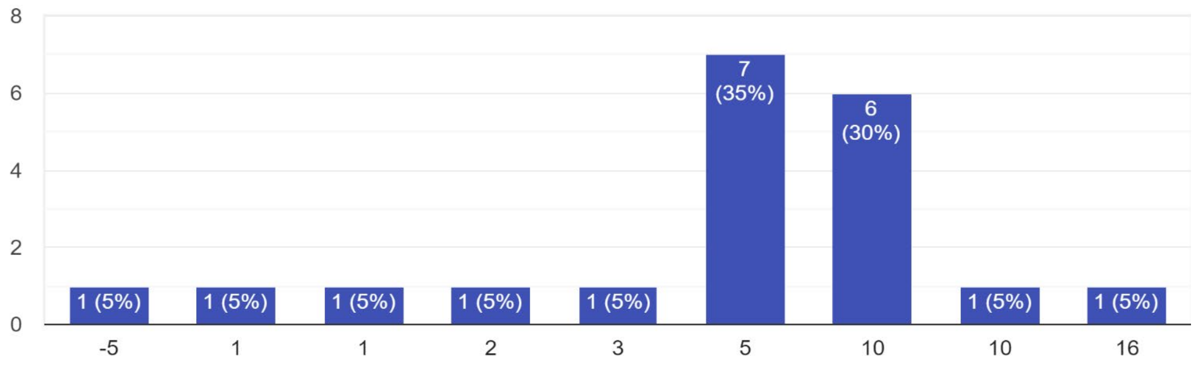

Current scientific evidence shows how each genetic mutation may be associated with a peak in the risk of dissection at a certain age. Following the previous question, is it best to consider

23 responses

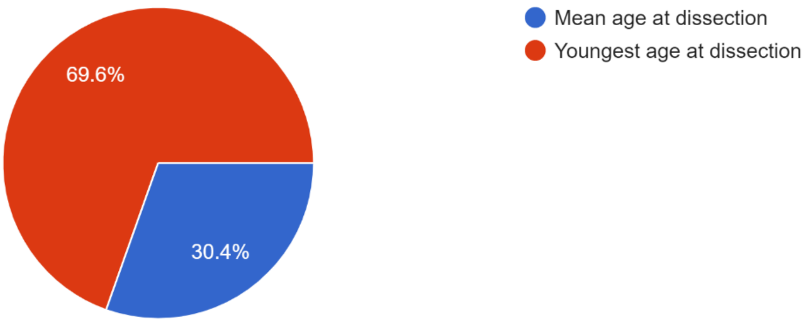

Should there be a upper age limit for offering genetic testing to the patient with a thoracic aortic disease?

24 responses

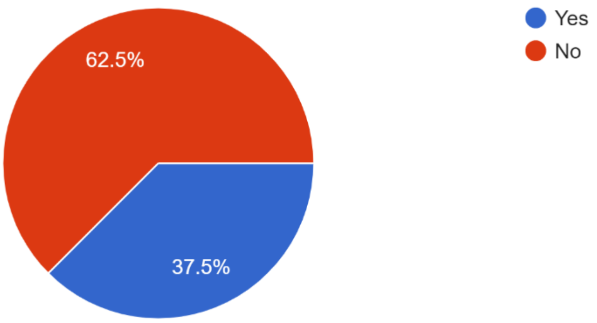

Which upper age limit should be considered?

9 responses

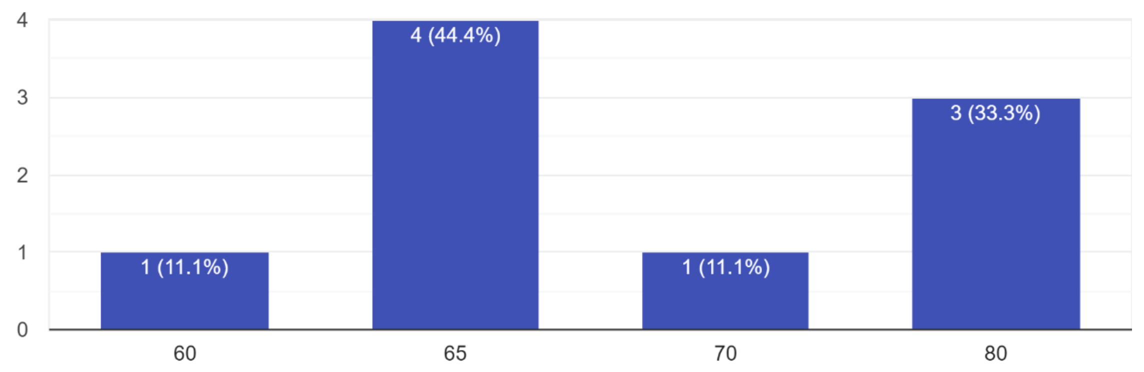

Select all relevant psychological tests that you think should be used to monitor the impact of the screening programme (depression).

10 responses

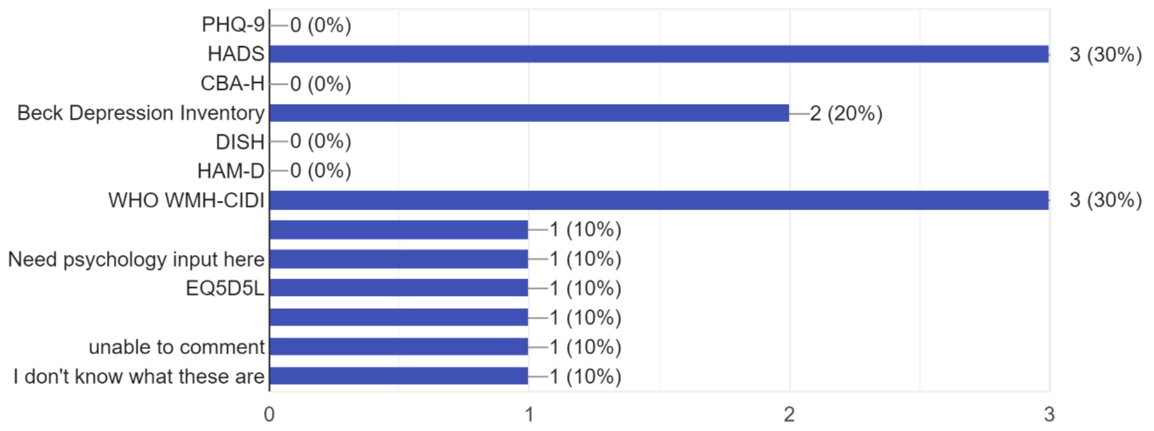

How should we look at our participants?

24 responses

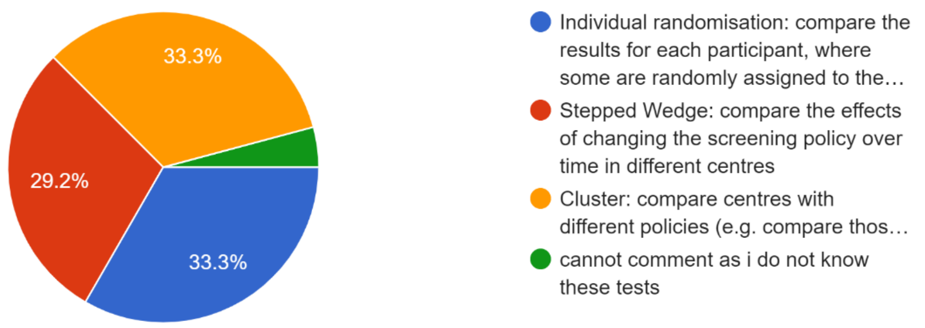

How many centres should be involved

22 responses

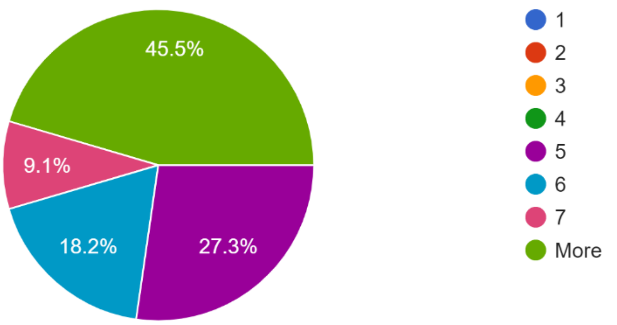

How long do you think it would take to change what is currently done for screening in your region?

22 responses

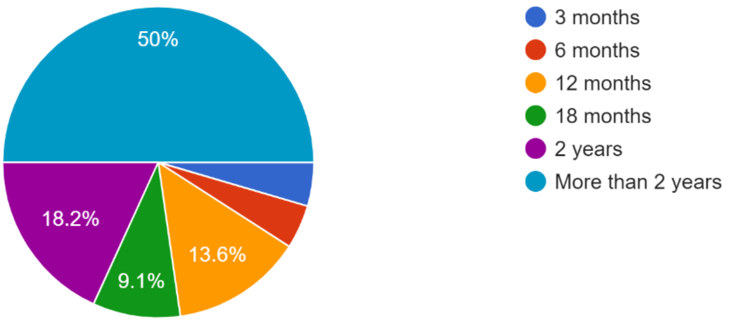

What tool should be used to measure Quality of Life?

15 responses

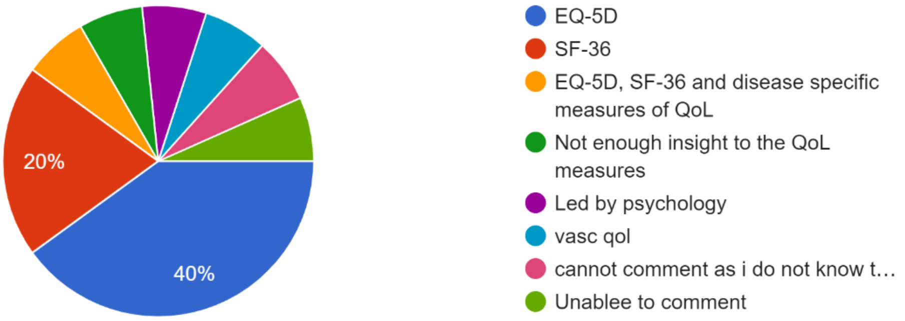

Rate the subsequent measures of effectiveness:

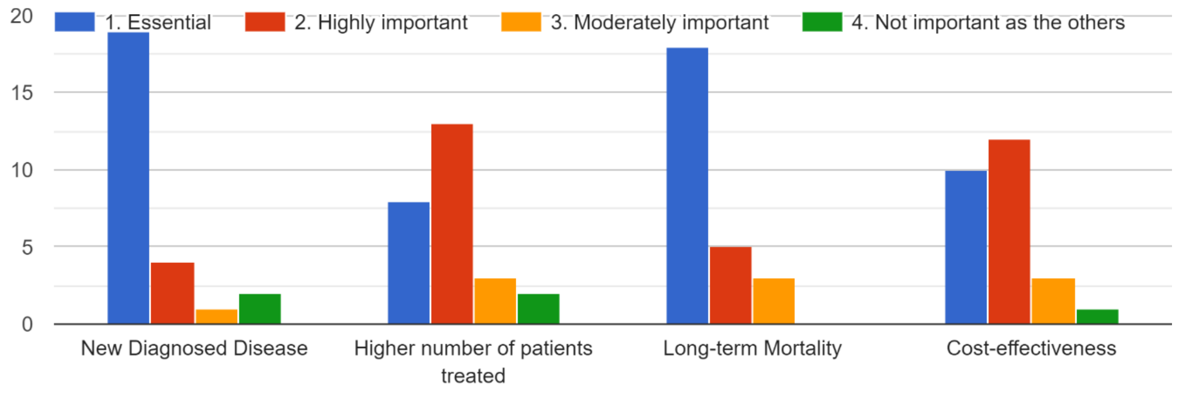

Which clinical events should be evaluated in this research? (select all relevant)

23 responses

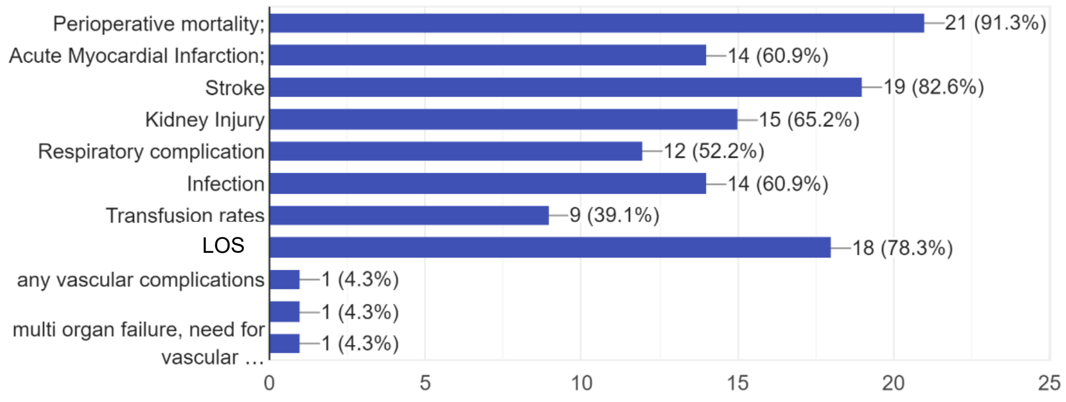

This research could expose families to a psychological stress. When should relatives involved in the test be monitored for signs of depression and anxiety?

22 responses

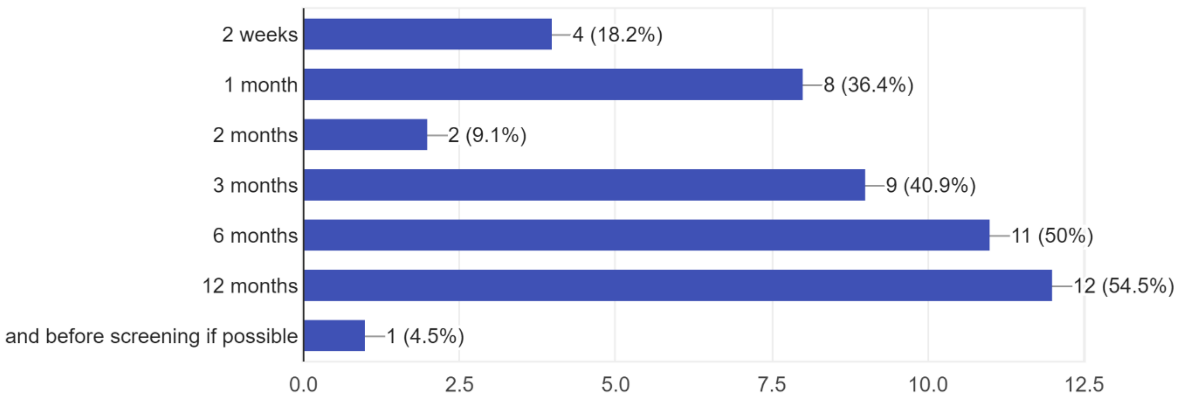

LAY VERSION

Which category describes you best?

46 responses

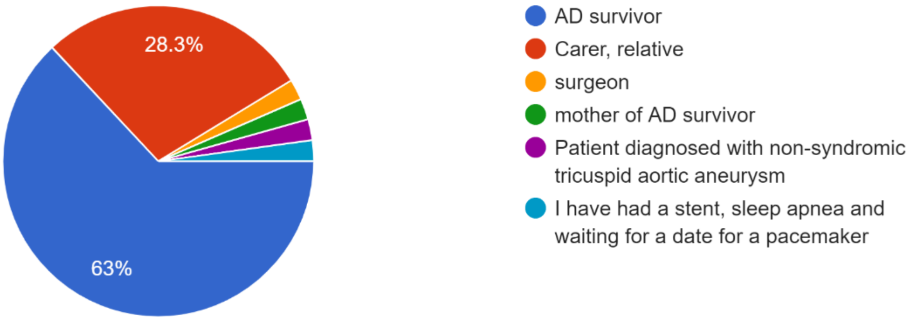

Do you think relatives of patients affected by non-syndromic aortic disease should undergo an imaging test? (gene...always identify a causative mutation)

45 responses

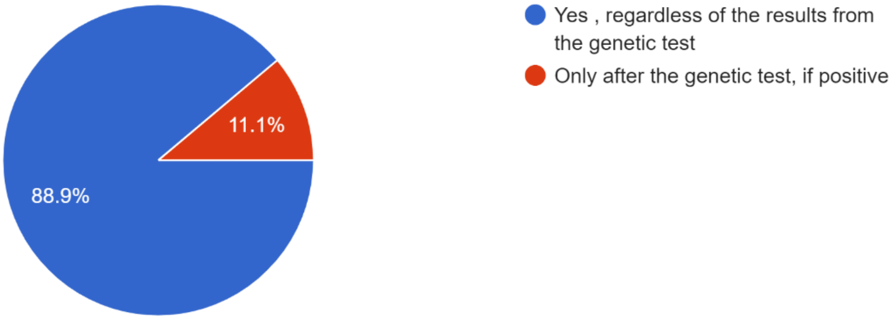

Which imaging test would you consider appropriate, in cases in which no clear genetic condition can be identified?

46 responses

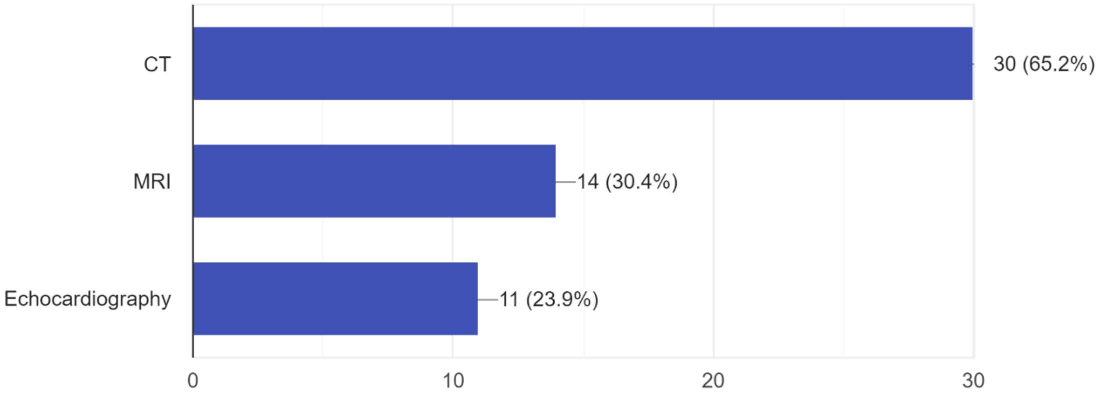

Which imaging test would you consider appropriate, in cases in which a genetic condition can be identified?

46 responses

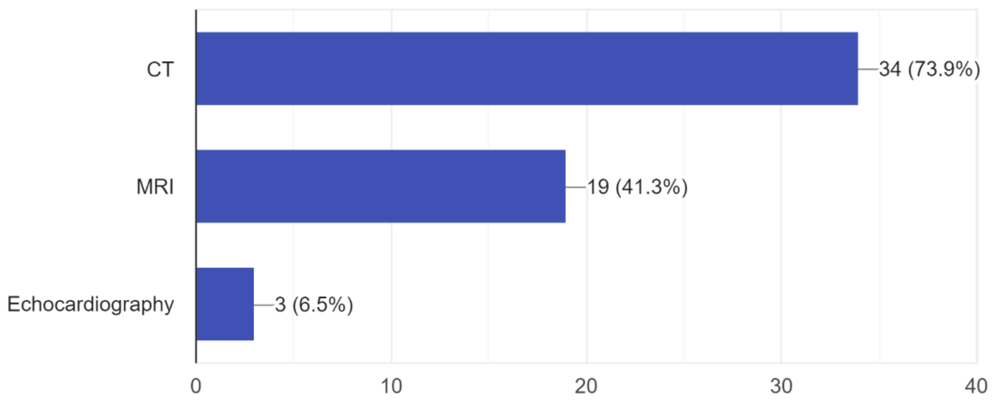

Starting from what age should relatives be screened with an imaging test?

16
14
18
18
30
20
18
13
as soon as their relative is well after surviving the AD
ASAP
16
Asap
As soon as they can understand the reason
18
18
Teens
Any
EIGHTEEN YEARS
Within 5 years of first relatives dissection
Early adult
20
ASAP
12 years of age
18
As soon as possible
As soon after the diagnosis as possible
12
35
7
I am unsure
16
Age at which proband was diagnosed, minus 20?

18 YEARS
16
45
21
Mid 30s
20
As early as possible
Any age
Maybe 18
40

Should doctors report additional findings from genetic tests?

46 responses

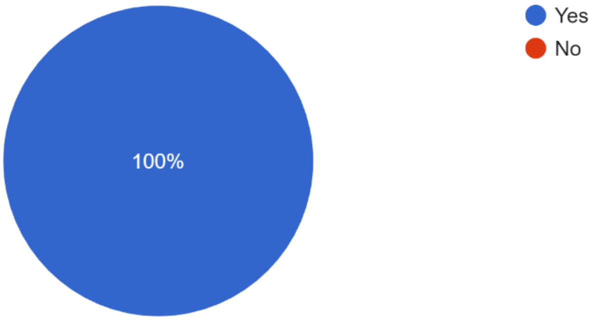

Should patients be consented about receiving a diagnosis of additional findings?

46 responses

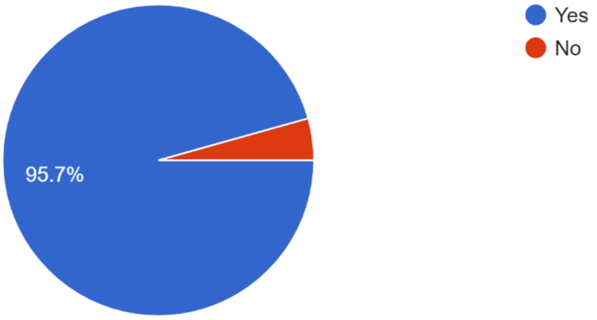

Who should be involved in Genetic Screening? (select all relevant)

46 responses

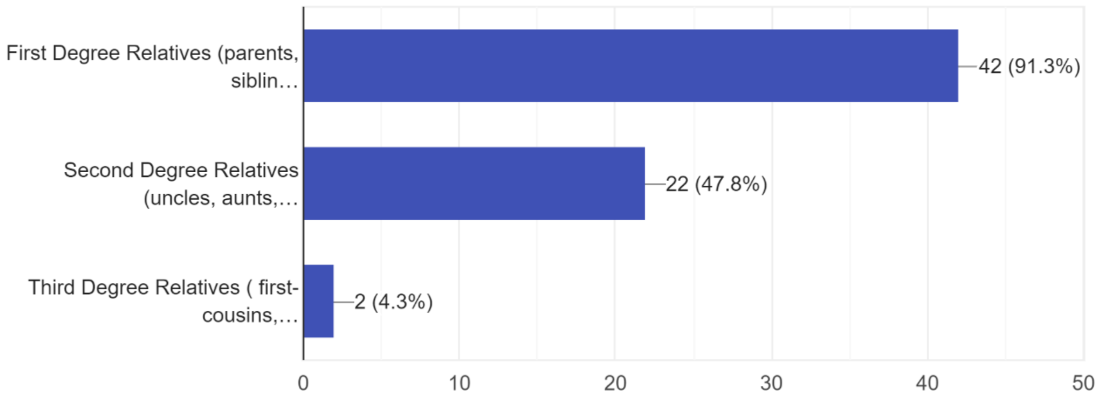

A patient/relative (with a previous negative or inconclusive genetic test result) would require re-testing when/if...

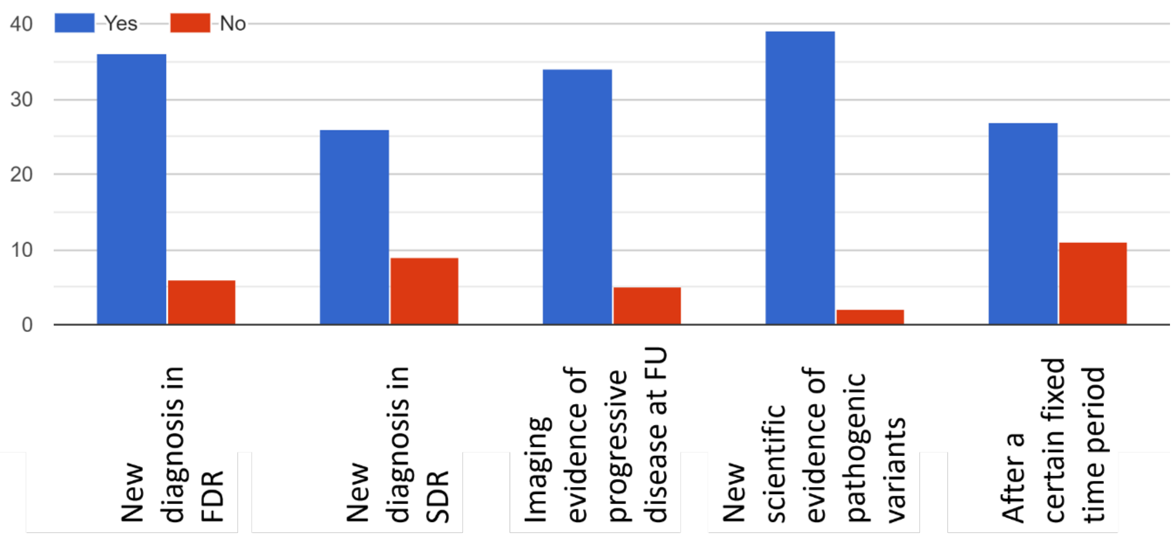

Is it appropriate to store a sample from a patient affected by aortic dissection in any case during an urgent operation, for the purpose of genetic testing?

46 responses

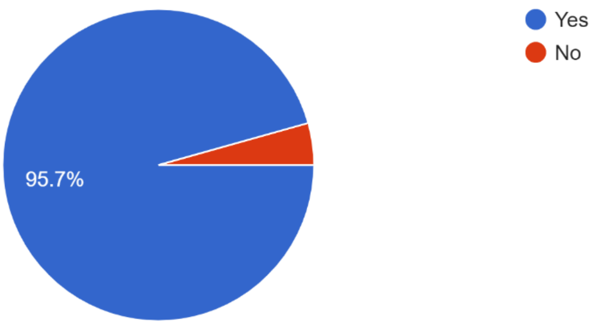

Is it appropriate to discuss genetic testing with the family after an urgent surgery for aortic dissection?

46 responses

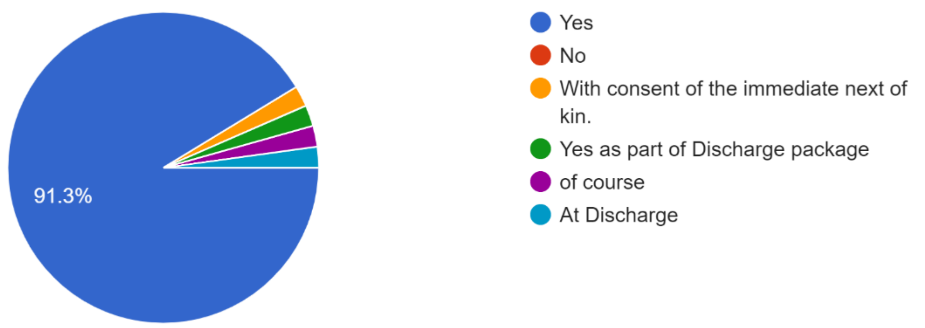

Is it appropriate to discuss genetic testing with the family after a patient dies from aortic dissection?

46 responses

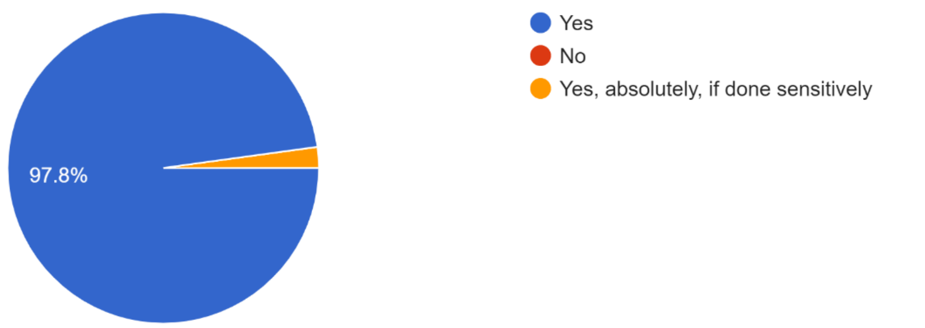

Who should be the professional figure involved in informing patients about genetic risk (and therefore referring them to a clinical geneticist)?

46 responses

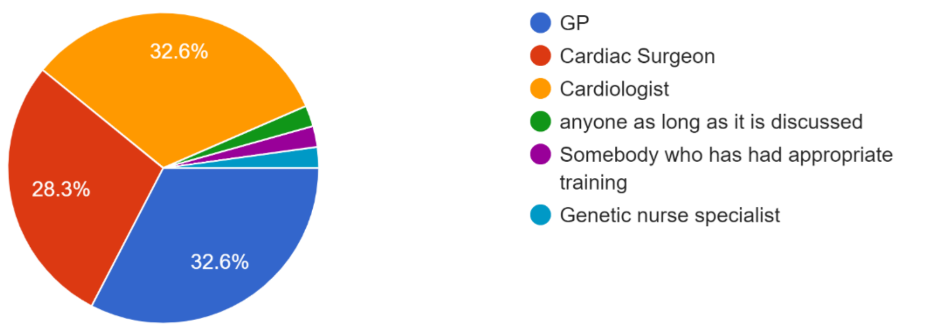

Should a Multidisciplinary Team be involved in the management of these families? What professional figures should be involved from the outset?

46 responses

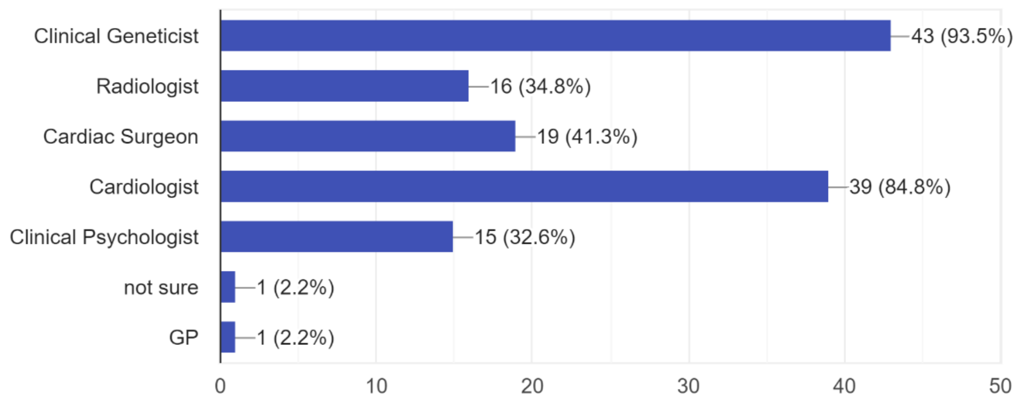

Current scientific evidence shows how each genetic mutation may be associated with a peak in the risk of dissection at a certain age. How many years before the youngest person dissects for that gene should we start surveillance?

40 responses

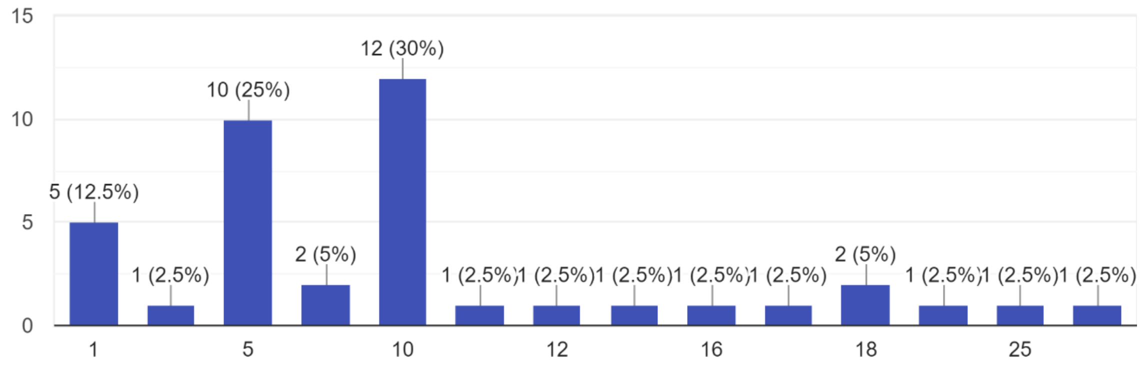

Current scientific evidence shows how each genetic mutation may be associated with a peak in the risk of di...revious question, is it best to consider

45 responses

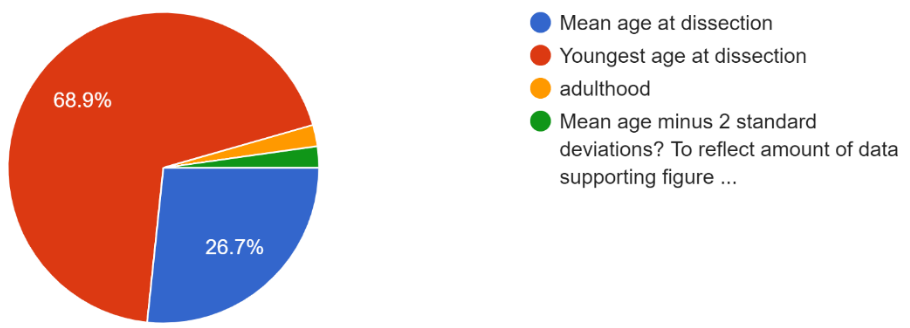

Should there be a upper age limit for offering genetic testing to the patient with a thoracic aortic disease?

44 responses

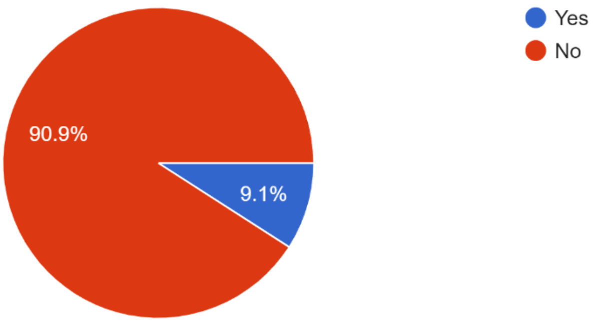

Which upper age limit should be considered?

12 responses

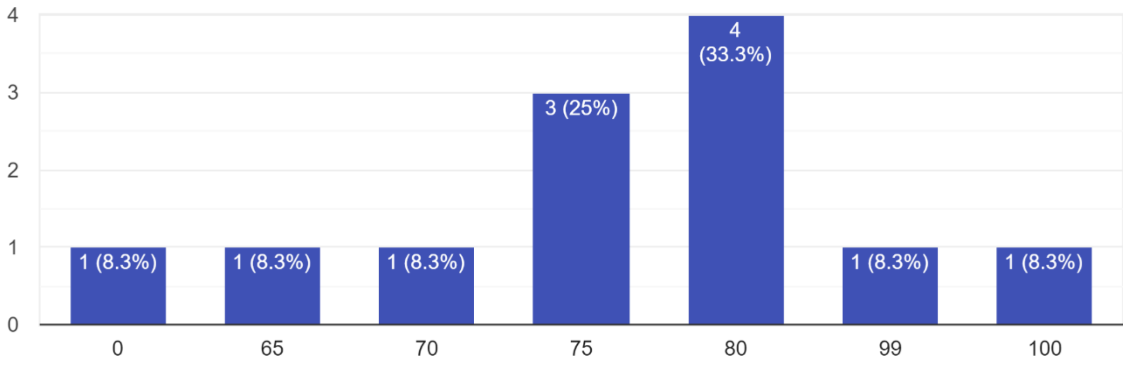

Do patients need additional psychological or social support in the post-operative period?

45 responses

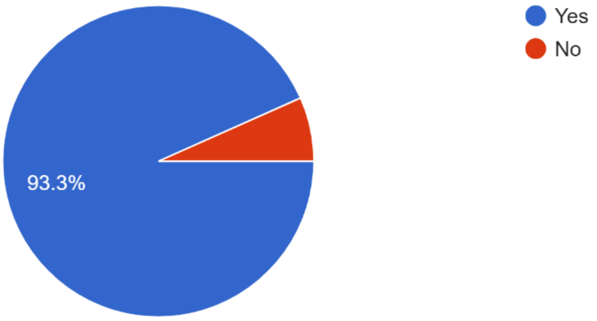

What kind of support do you think would be necessary?

41 responses

How should we look at our participants?

44 responses

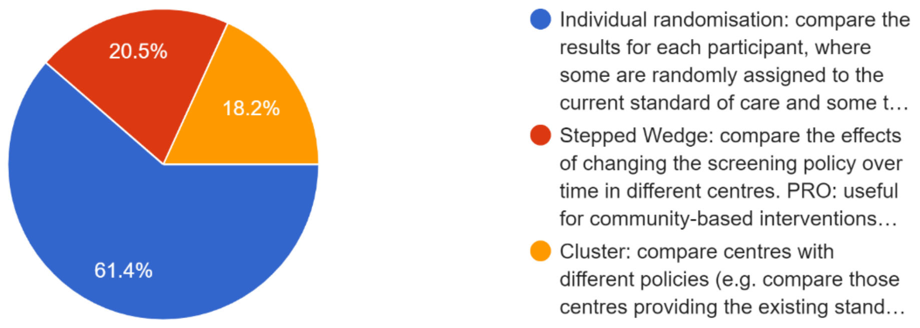

How long do you think it would take to change what is currently done for screening in your region?

45 responses

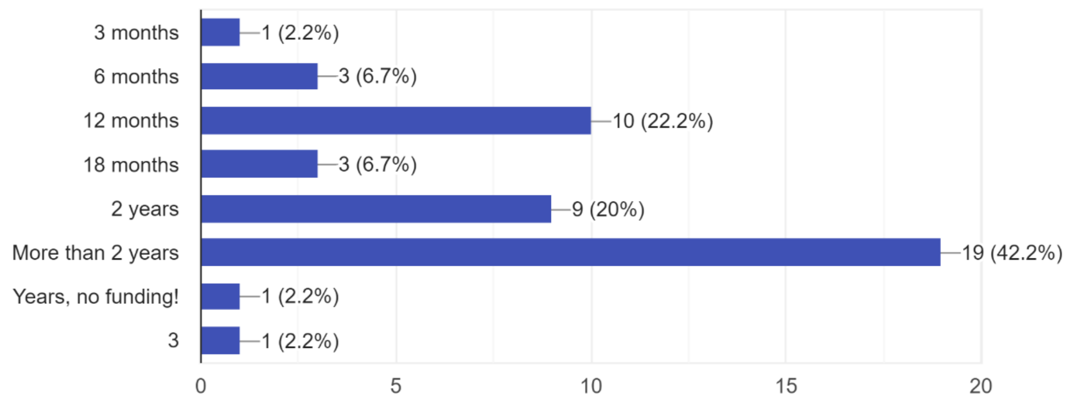

What would you consider the most valuable result of a new approach to screening?

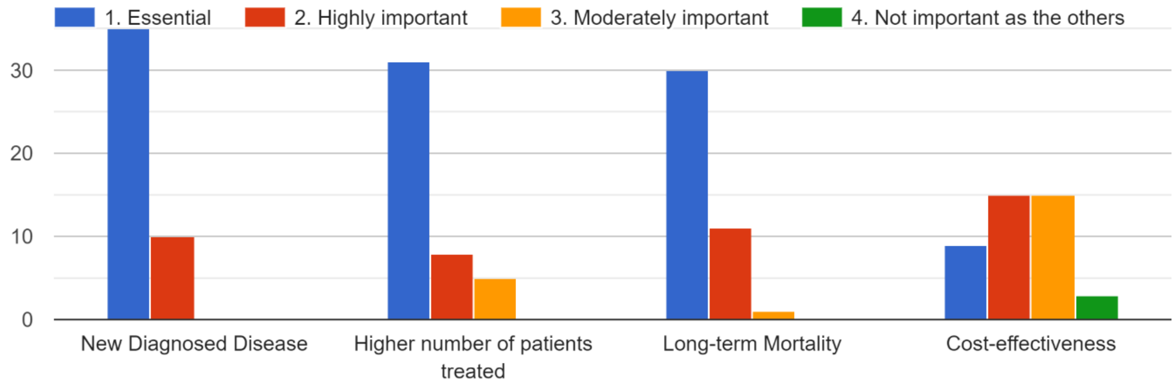

Which clinical events should be evaluated in this research? (select all relevant)

46 responses

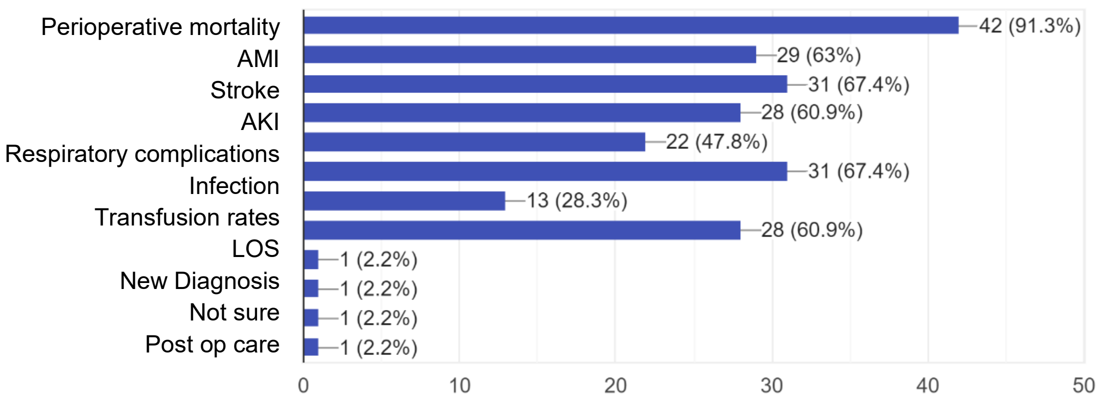

This research could expose families to a psychological stress. When should relatives involved in the test be monitored for signs of depression and anxiety?

46 responses

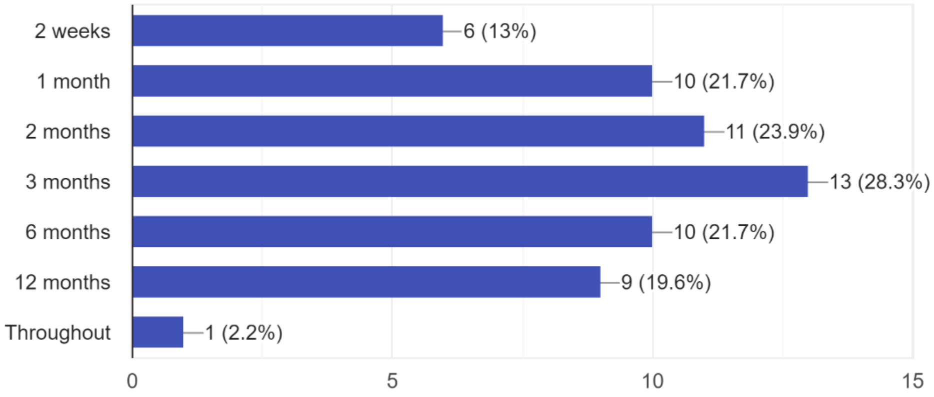

Supplement: Supplementary file 5 — Additional file 5. [file 13063_2020_4562_MOESM5_ESM.pdf]
