# Supplementary material for: Report of a Delphi exercise to inform the design of a research programme on screening for thoracic aortic disease
Source: Trials. 2020 Jul 16;21:656. doi: 10.1186/s13063-020-04562-1 (PMC7367380; doi:10.1186/s13063-020-04562-1)
Supplement: Supplementary file 6 — Additional file 6. [file 13063_2020_4562_MOESM6_ESM.pdf]

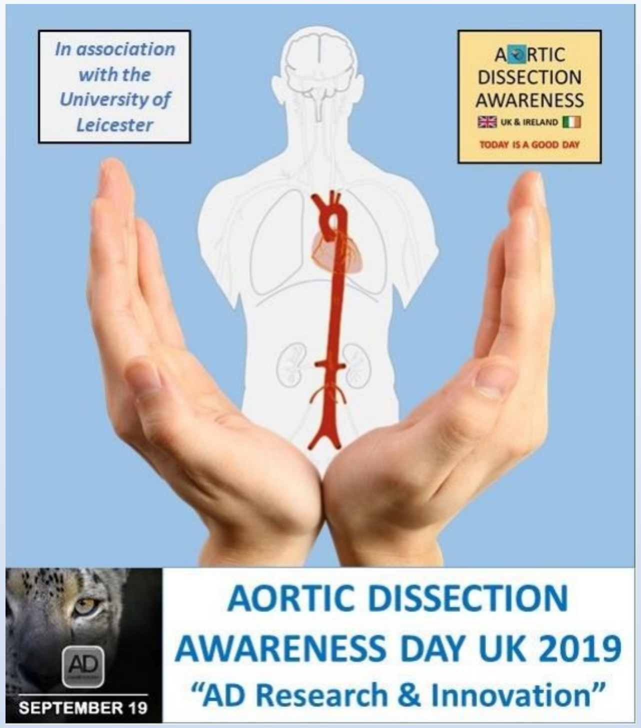

Do you think relatives of patients affected by non-syndromic aortic disease should undergo an imaging test?

20 responses

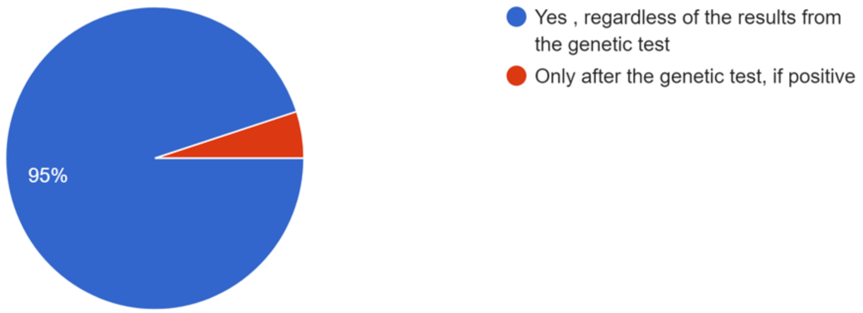

- Family members can present with aneurysm without positive genetic test. Incidence can be up to 20%
- Yes is if we are unsure. IF they are confirmed to not carry a -familial mutation which we are sure is the cause of disease they do not need screening
- There are still many unknown genes hence relying on only positive genetic tests will miss numbers of cases
- There is clear evidence of oligogenic inheritance or complex inheritance which may place relatives at risk even where they are genotype negative, or genotype unknown. Where there is a clearly and definitively monogenic cause of aortopathy in the proband, and this is absent in the relatives, this may be a different situation, and relatives may then be discharged without imaging, but this is fairly rare in my experience.

Which imaging test would you consider appropriate, in cases in which no clear genetic condition can be identified?

19 responses

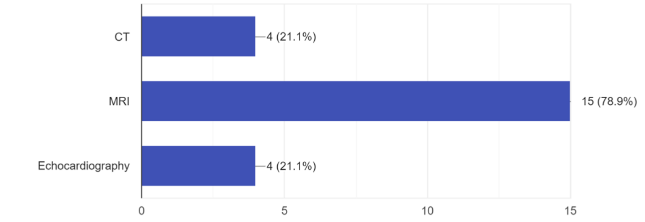

- For screening purpose MRI of whole aorta and aortic valve is a reasonable test.
- needs best procedure and hence MRI... wherever possible
- Use of CT depends on age, suitability for MRI. TTE is of very limited value except for "obvious" cases in certain aortic locations only, TOE may be better. Echos may give a false negative
- MRI good for aortic measurements but not reliable in aortic dissections / ulcers
- Echo

Starting from what age should relatives be screened with an imaging test?

16 responses

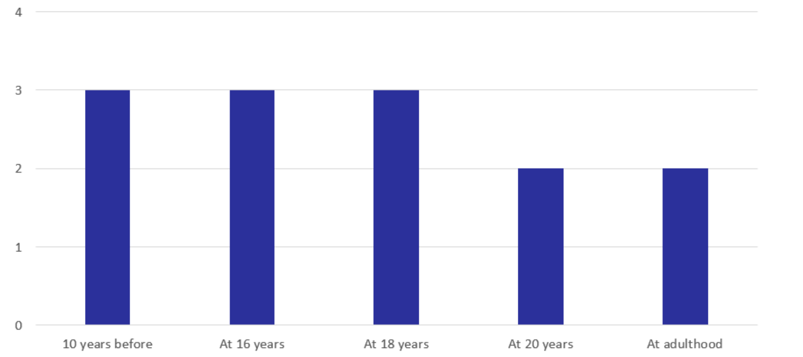

Which imaging test would you consider appropriate, in cases in which a genetic condition can be identified?

11 responses

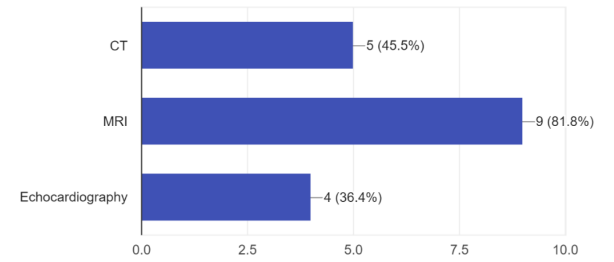

What should be the method of choice for Follow Up in Relatives with an uncertain genetic variant?

19 responses

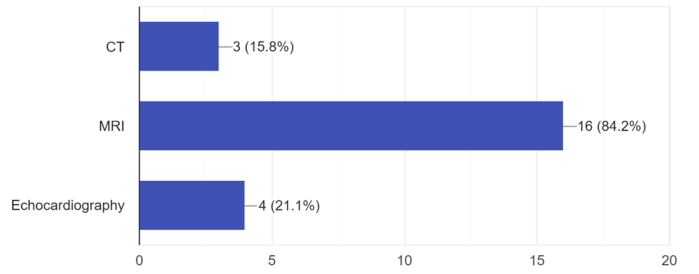

- As above
- Use of CT depends on age, suitability for MRI. TTE is of very limited value except for "obvious" cases in certain aoritc locations only, TOE may be better
- CT

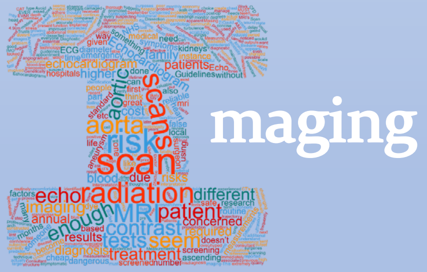

Aortic Dissection Awareness Day UK *Delphi*

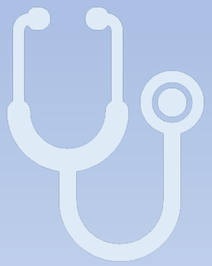

What should be the optimal follow-up rate in each of these cases?

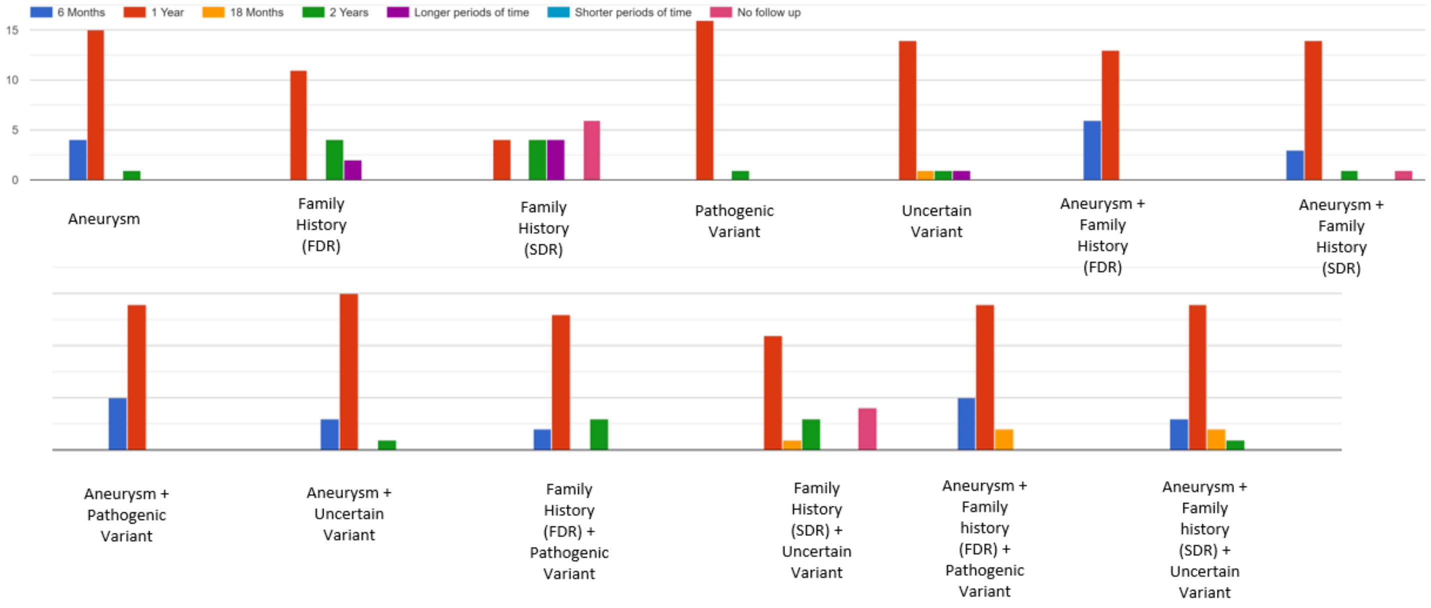

- Depends on the genotype and phenotype
- Follow up in secondary relatives depends on presence of aneurysm on screening and/or strong prevalence of aneurysm in first degree relatives
- shorter for high risk variants and phenotype
- I think all these should be tailored to the individual history - in new aneurysm diagnosis, I would re-screen within 6 months to ensure no rapid growth, and then, so long as it was not huge, I would consider a switch to annual follow-up in the absence of high-risk features, for example. Family history - it would depend on the severity and age of onset of the disease in the family.
- F/U intervals should also consider the aneurysm size.

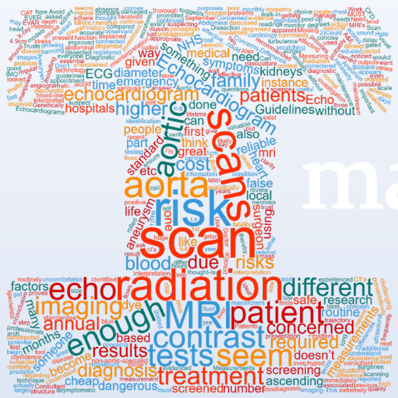

maging

Do you think relatives of patients affected by non-syndromic aortic disease should undergo an imaging test? (genetic testing cannot always identify a causative mutation)

83 responses

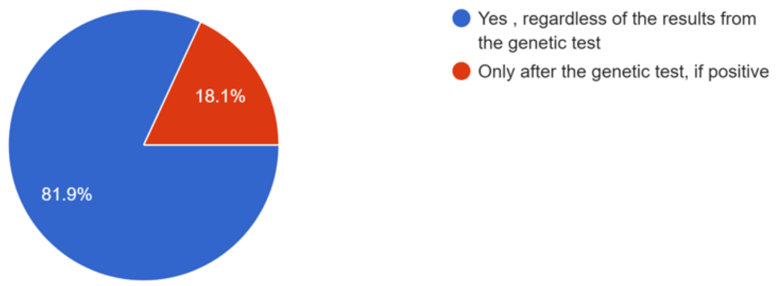

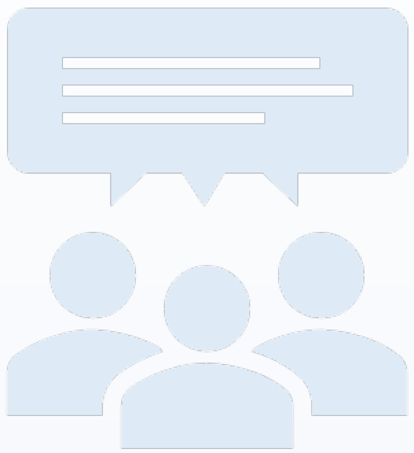

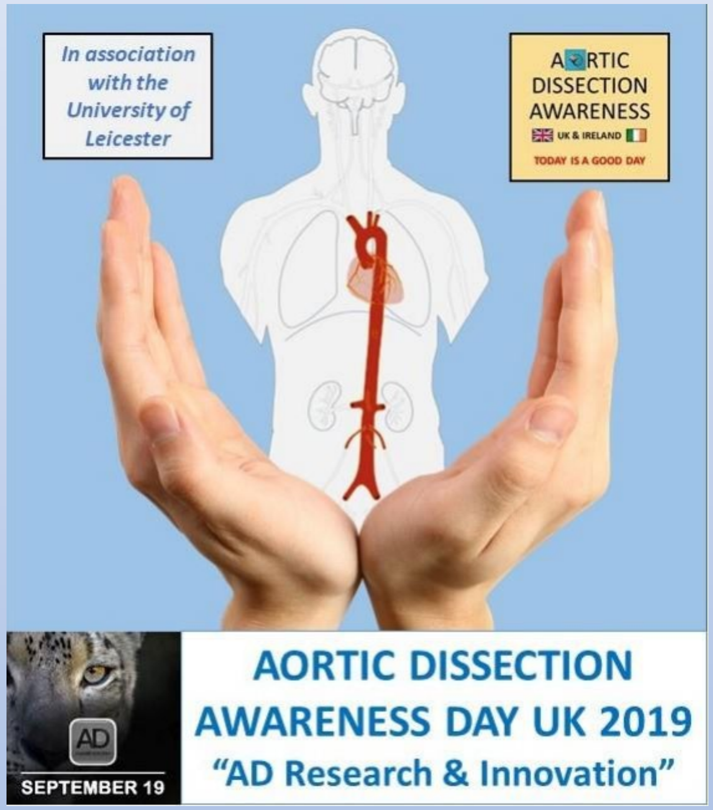

Starting from what age should relatives be screened with an imaging test?

75 responses

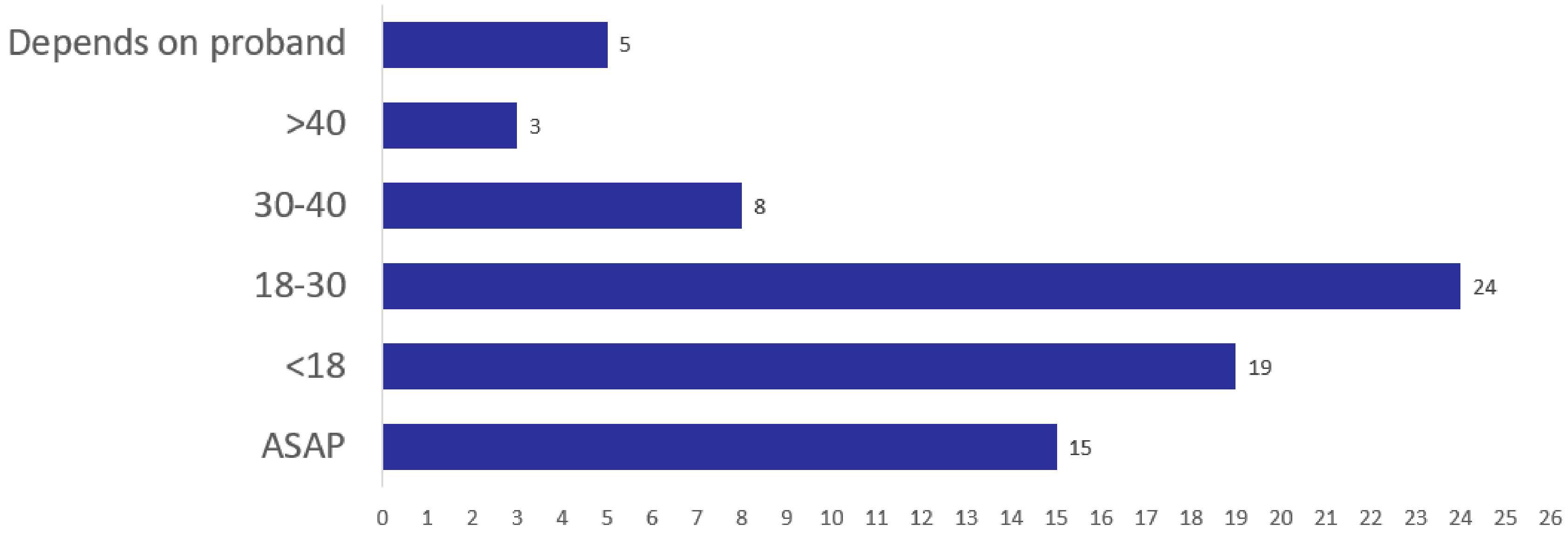

Aortic Dissection Awareness Day UK *Delphi*

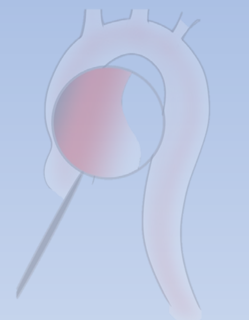

Which imaging test would you consider appropriate, in cases in which a genetic condition can be identified?

84 responses

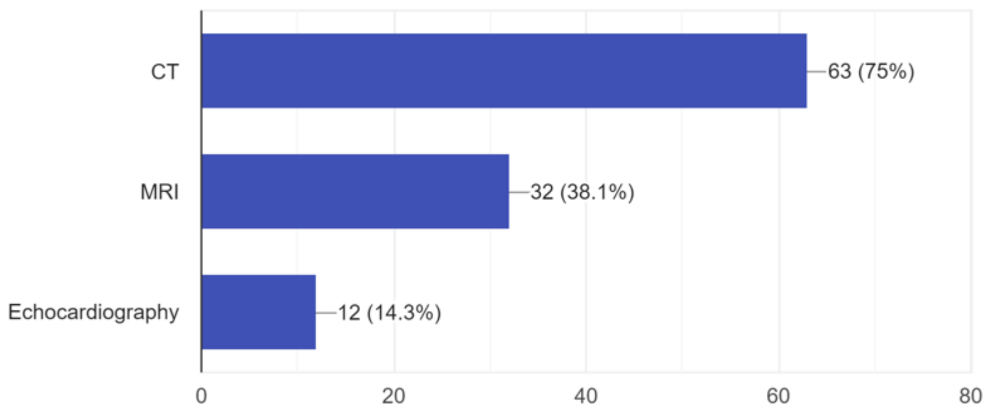

Which imaging test would you consider appropriate, in cases in which no clear genetic condition can be identified?

84 responses

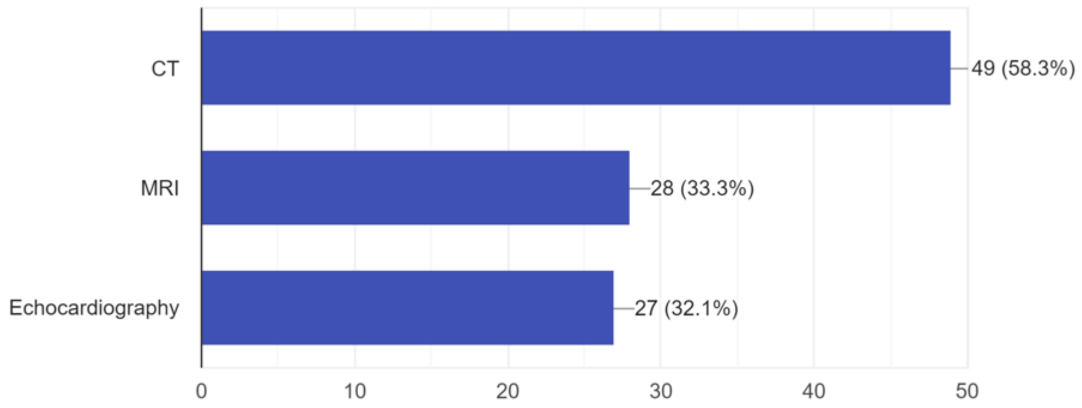

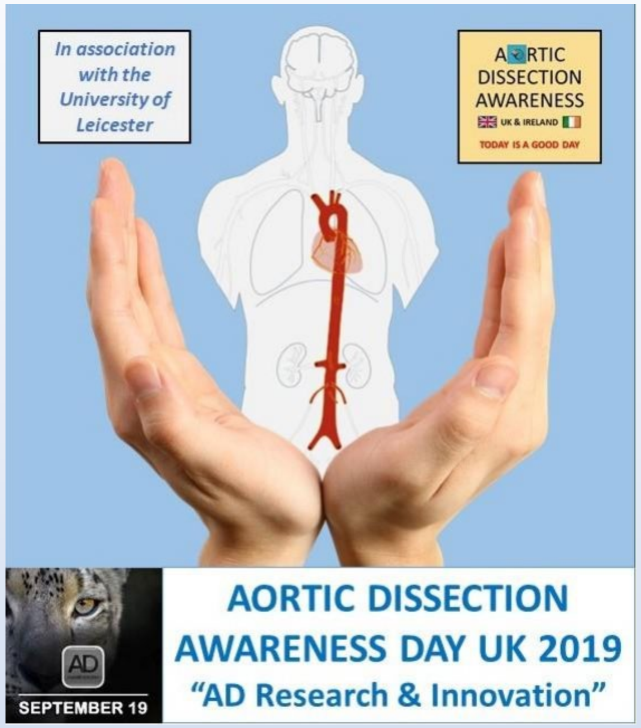

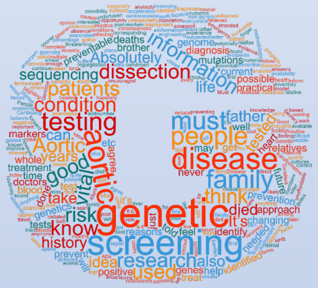

enetic Testing

Aortic Dissection Awareness Day UK *Delphi*

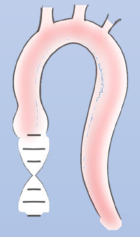

Should incidental findings be a reason to adopt a more focused test?

19 responses

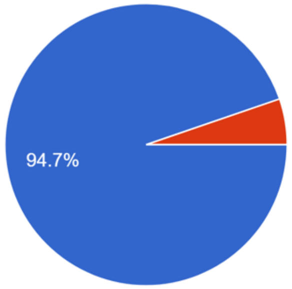

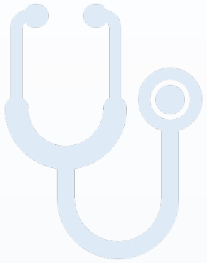

Who should be involved in Genetic Screening?

20 responses

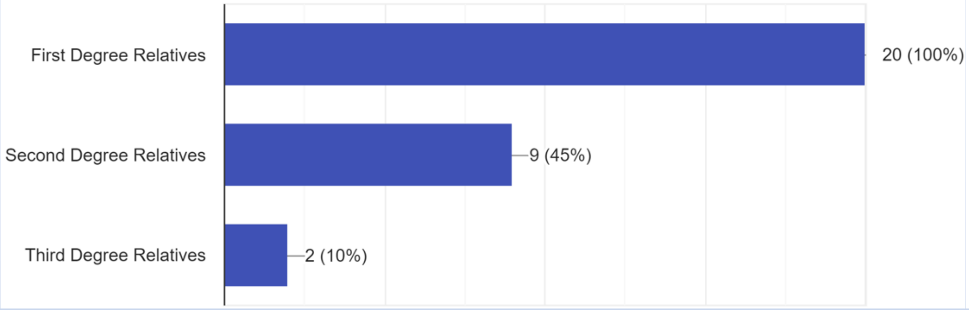

- Secondary relatives if first degree relatives are positive
- Depends on result in proband
- cascade 1- then second if at risk etc
- 3rd degree if and when they become 1st or 2nd degree to a finding
- Third degree relatives when at risk or if helpful for family studies of uncertain variants
- This would depend on severity and age of onset, as well as genetic findings in the proband.
- First

A patient (with a previous negative or inconclusive genetic test result) would require re-testing when/if...

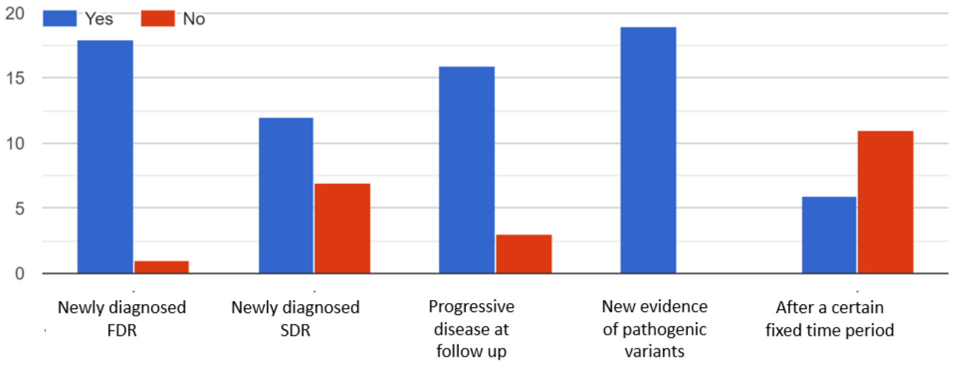

- Depends if the panel of markers has been updated since the previous test!
- This uncertainty could be addressed in the trial
- I think the definition of "new scientific evidence of pathogenic variants available" is a little tricky - in practice, new reports in ClinVar are made almost weekly by clinical services, so information about variant pathogenicity is constantly being refined. I think pragmatically, testing after a certain fixed time period would be the only way to incorporate these incremental advances in the science.

Is it appropriate to discuss genetic testing with the family after a patient dies from aortic dissection?

19 responses

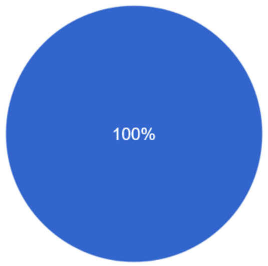

- Immediately
- No consent required in this scenario.

Is it appropriate to store a sample from a patient affected by aortic dissection in any case during an urgent operation?

19 responses

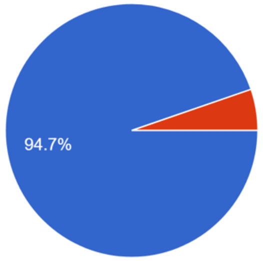

- always and in the appropriate way so check with labs

Is it appropriate to discuss genetic testing with the family after an urgent surgery for aortic dissection?

19 responses

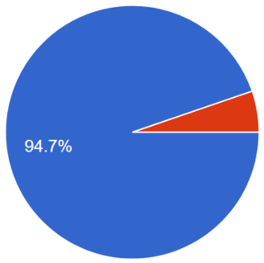

- As may have family implications and after the event may be too late
- You need patient consent to do this. ok, if you have it.

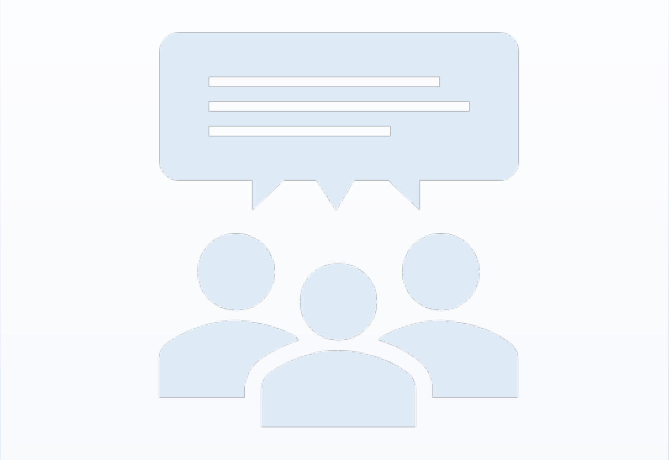

Should doctors report additional findings from genetic tests?

84 responses

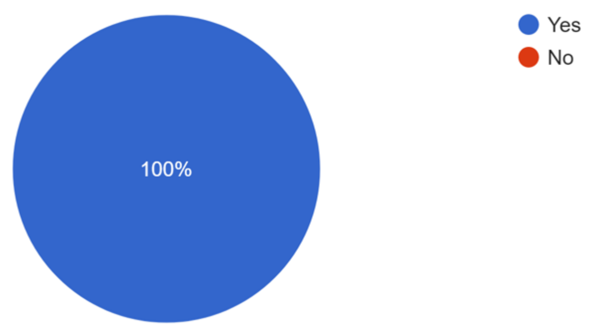

Is it appropriate to store a sample from a patient affected by aortic dissection in any case during an urgent operation?

84 responses

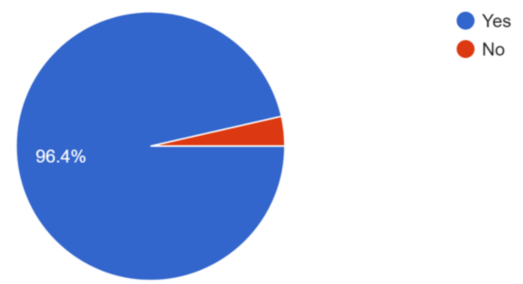

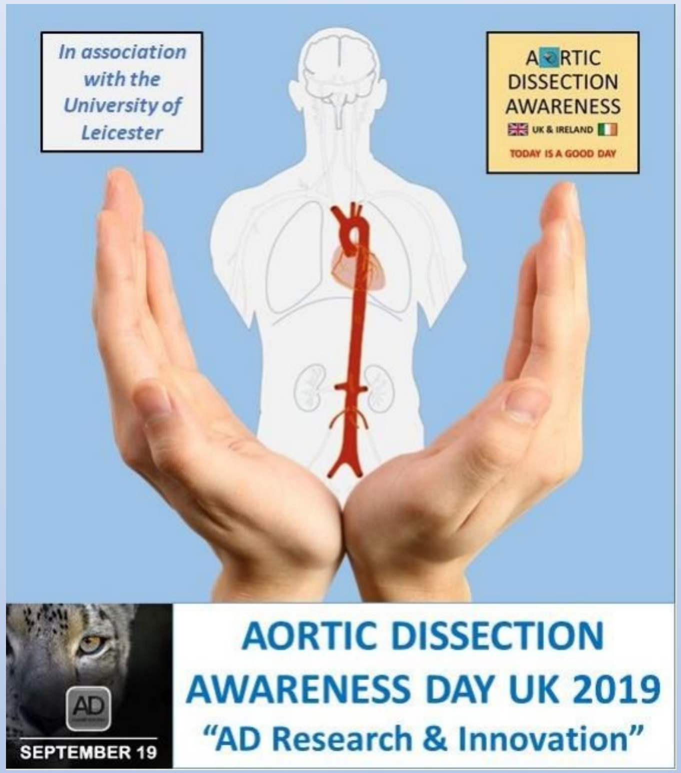

Should patients be consented about receiving a diagnosis of additional findings?

84 responses

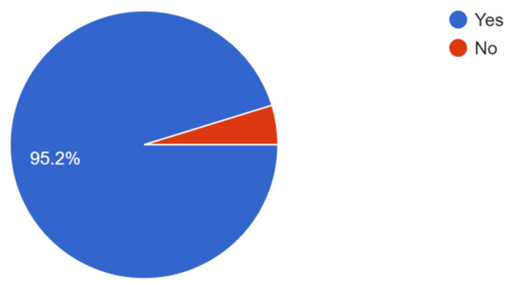

Is it appropriate to discuss genetic testing with the family after a patient dies from aortic dissection?

84 responses

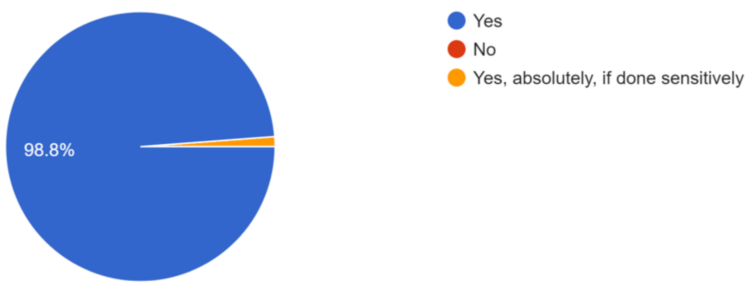

Who should be involved in Genetic Screening? (select all relevant)

84 responses

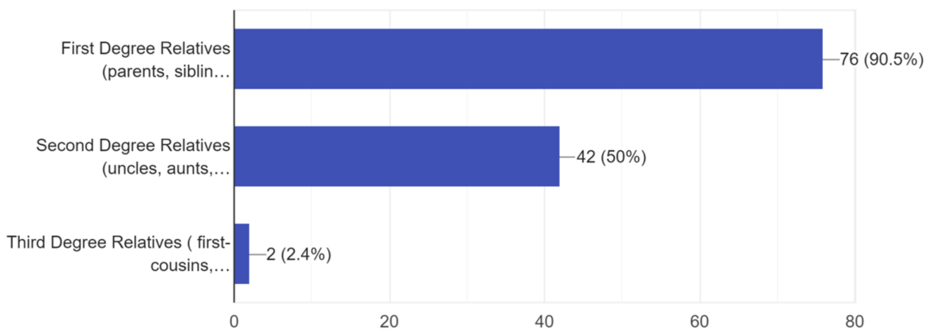

Aortic Dissection Awareness Day UK *Delphi*

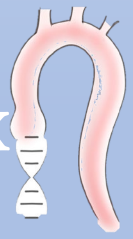

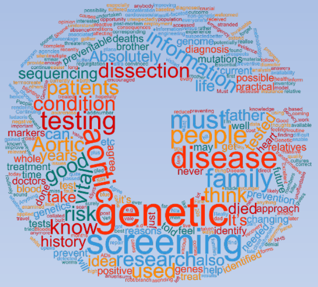

enetic Testing

A patient/relative (with a previous negative or inconclusive genetic test result) would require re-testing when/if...

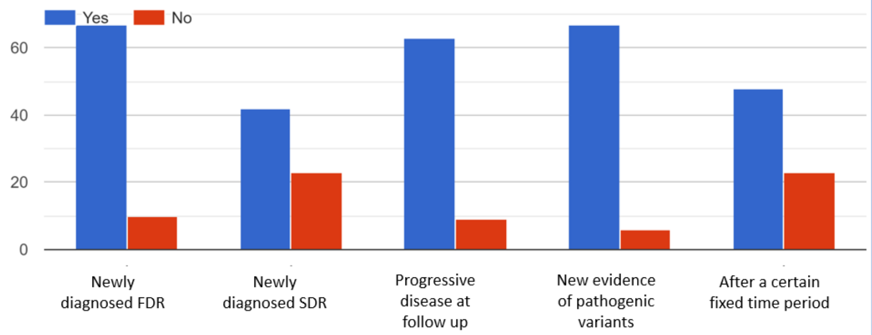

Is it appropriate to discuss genetic testing with the family after an urgent surgery for aortic dissection?

84 responses

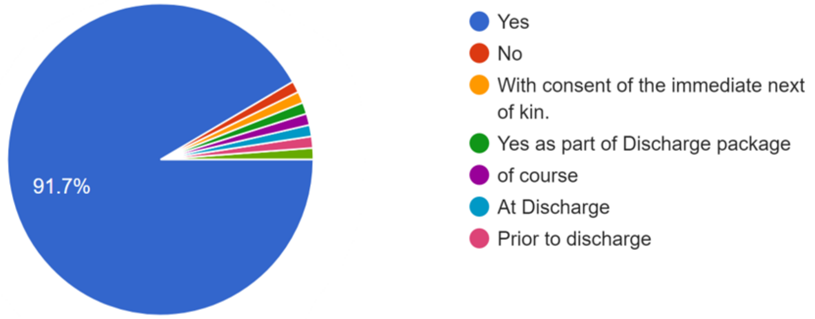

Who should be the professional figure involved in informing patients about genetic risk (and therefore referring them to a clinical geneticist)

19 responses

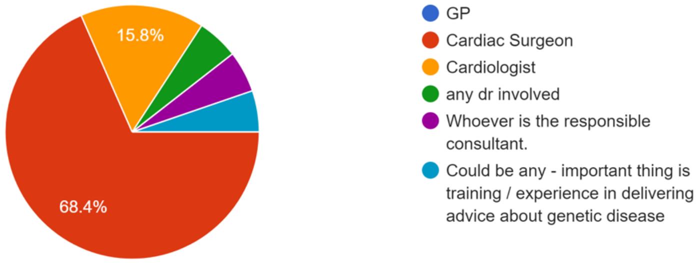

Current scientific evidence shows how each genetic mutation may be associated with a peak in the risk of dissection at a certain age. Following the previous question, is it best to consider

19 responses

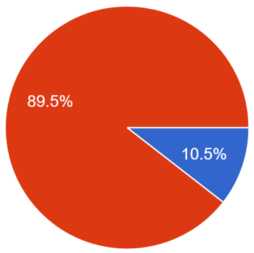

Important to identify as relative may need to know for themselves

They will have potentially affected relatives who are younger

Should there be a upper age limit for offering genetic testing to the patient with a thoracic aortic disease?

19 responses

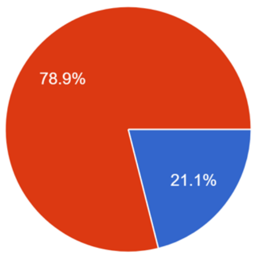

- both are important... if a one off person died very young it is not appropriate to screen all the family if we know the condition is usually later onset

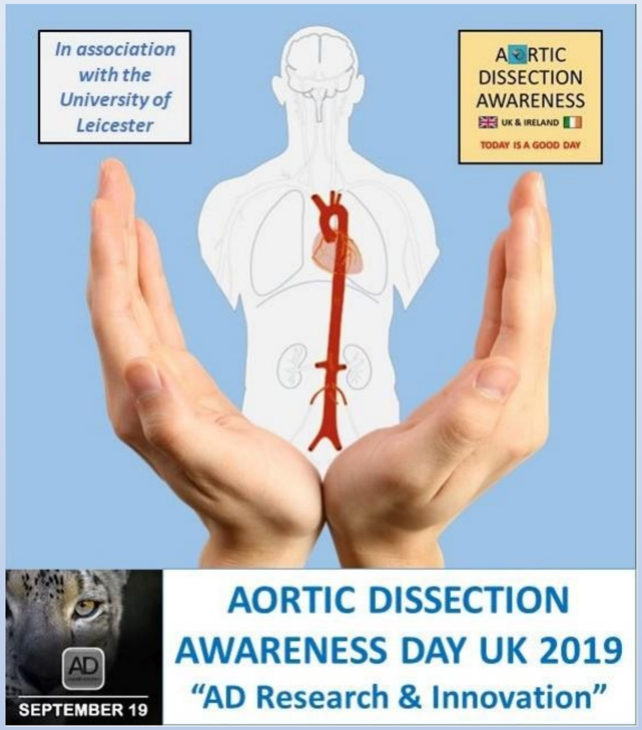

Should a Multidisciplinary Team be involved in the management of these families? What professional figures should be involved?

19 responses

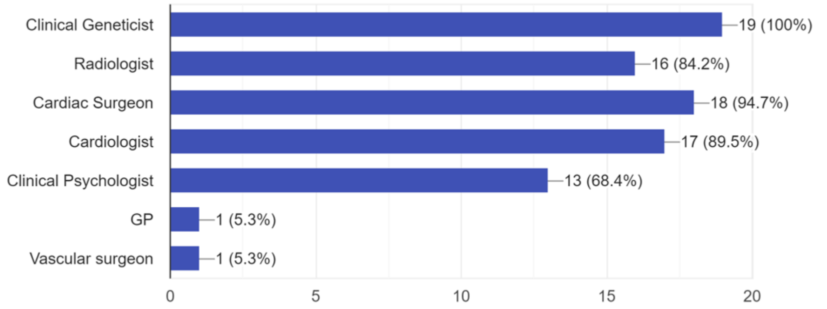

- Whoever makes referral needs to be aware of the need to do so in the first place.

Which upper age limit should be considered?

13 responses

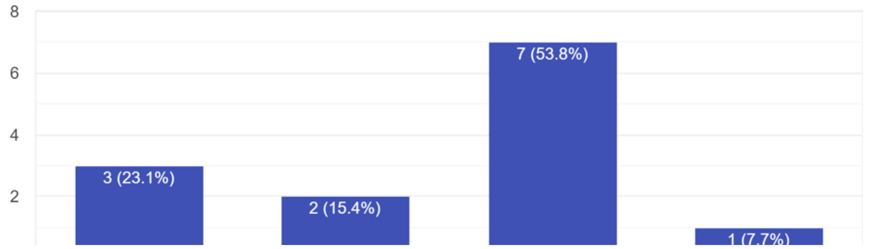

- None
- None - it could still have implications for family, particularly if higher risk features
- As age goes up, degenerative disease may confound genetic disease

Current scientific evidence shows how each genetic mutation may be associated with a peak in the risk of dissection at a certain age. How many years before the youngest person dissects for that gene should we start surveillance?

15 responses

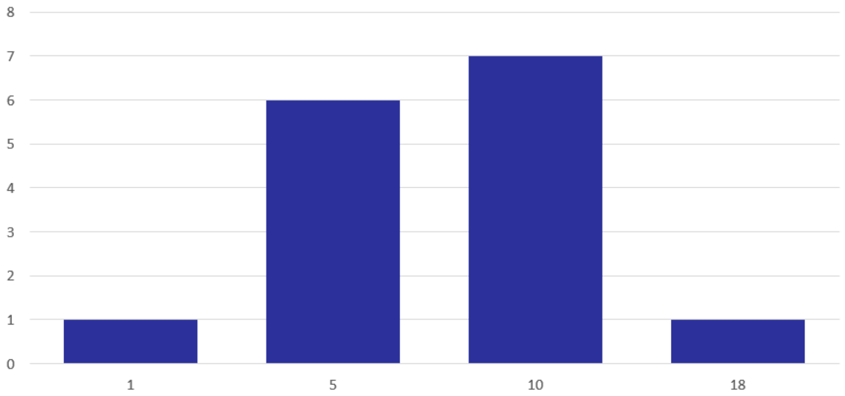

- 5 years.
- 10
- Do we wait for the aneurysm phenotype in high risk variants? We do in syndromics.
- Not sure (2-5 yrs range).

Select all relevant psychological tests that you think should be used to monitor the impact of the screening programme (depression).

9 responses

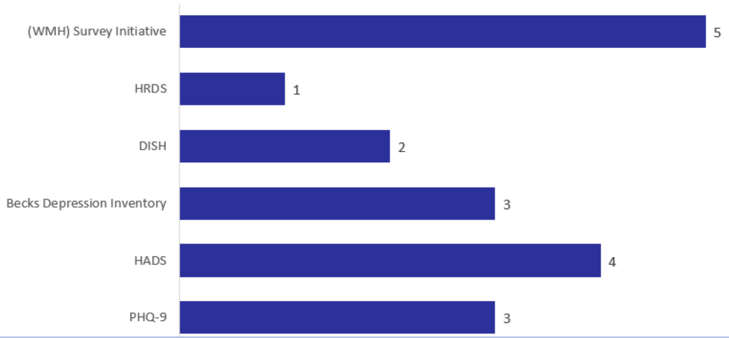

Select all relevant psychological tests that you think should be used to monitor the impact of the screening programme (anxiety).

8 responses

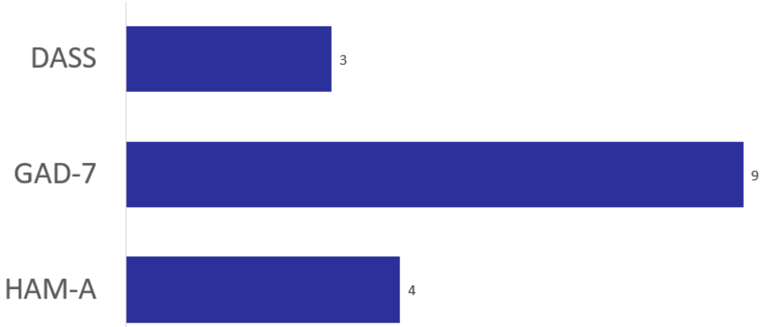

Aortic Dissection Awareness Day UK *Delphi*

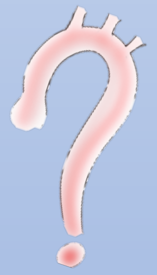

Genetic Counseling

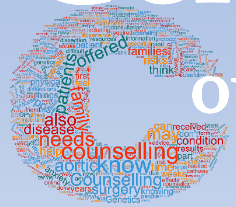

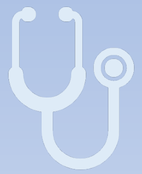

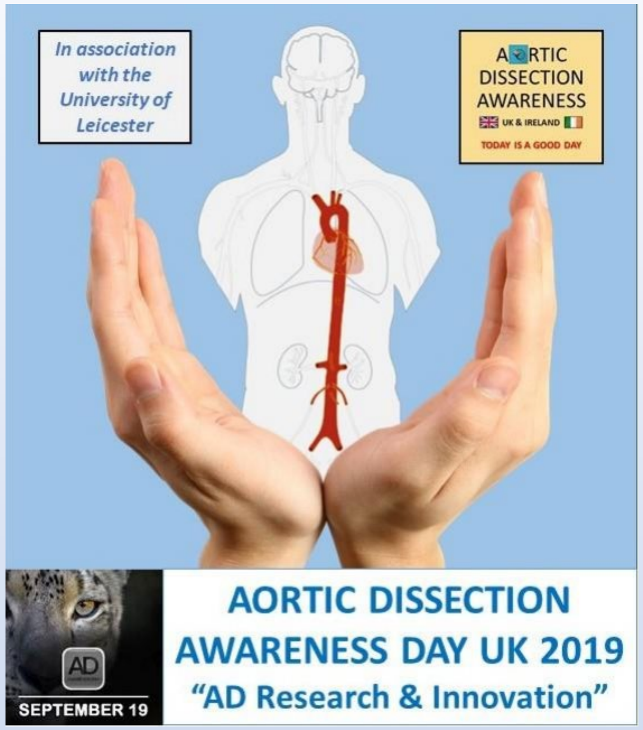

Should a Multidisciplinary Team be involved in the management of these families? What professional figures should be involved from the outset?

84 responses

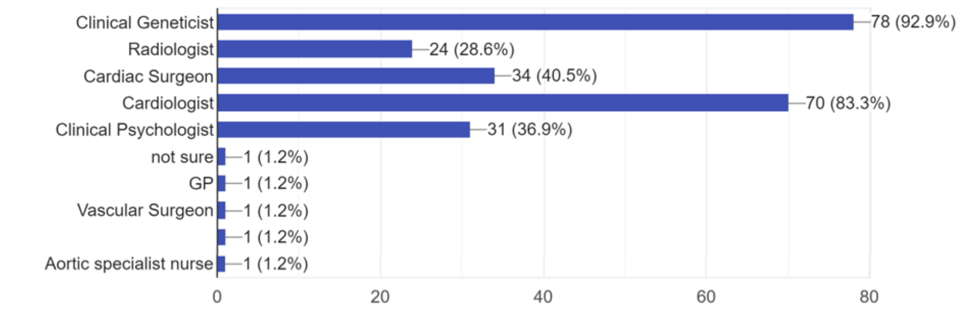

Current scientific evidence shows how each genetic mutation may be associated with a peak in the risk of dissection at a certain age. Following the previous question, is it best to consider

82 responses

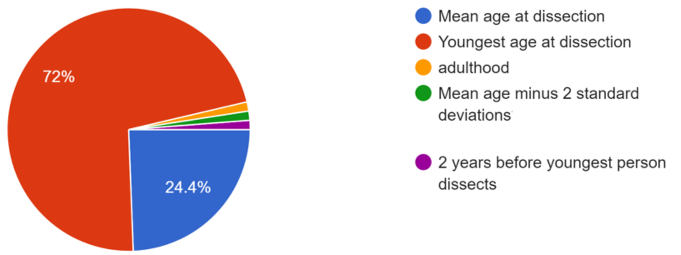

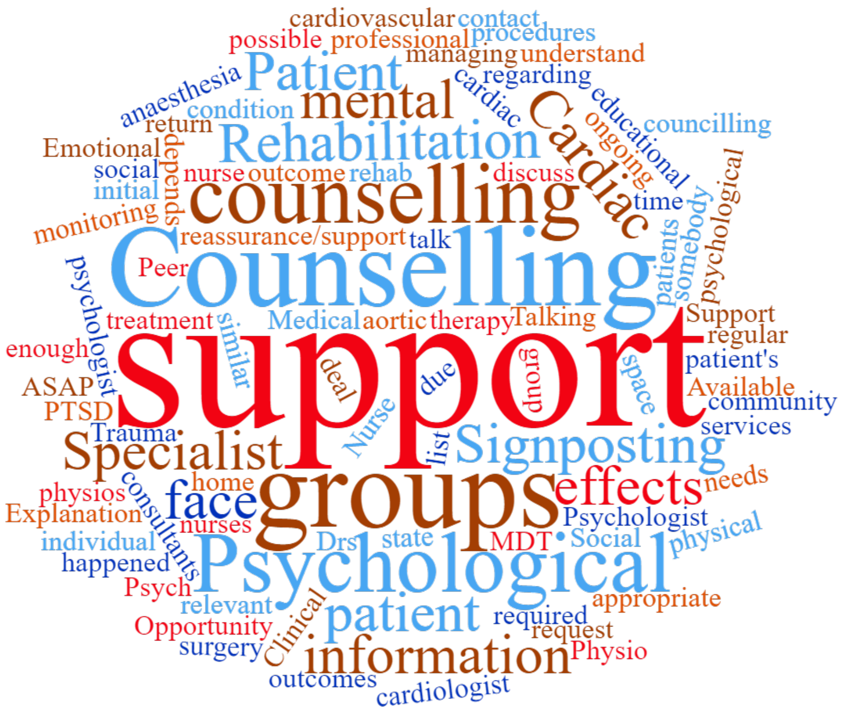

Current scientific evidence shows how each genetic mutation may be associated with a peak in the risk of dissection at a certain age. How many years before the youngest person dissects for that gene should we start surveillance?

75 responses

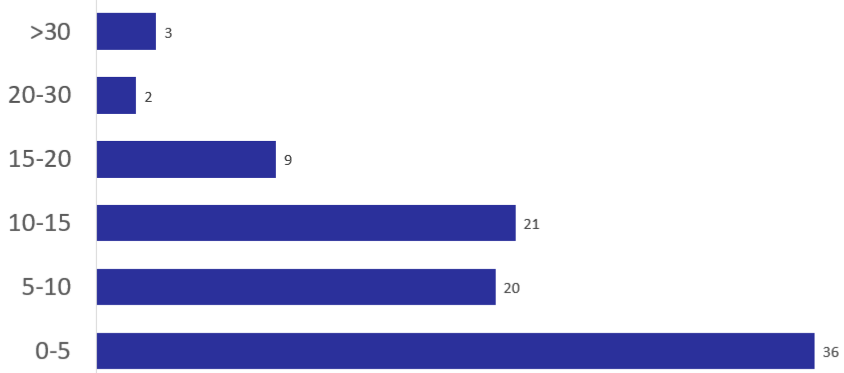

Do patients need additional psychological or social support in the post-operative period?

82 responses

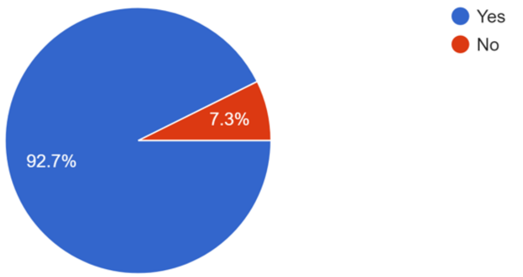

Should there be a upper age limit for offering genetic testing to the patient with a thoracic aortic disease?

82 responses

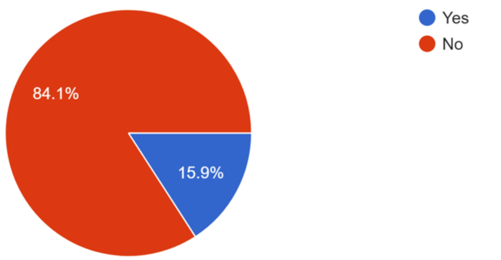

Which upper age limit should be considered?

28 responses

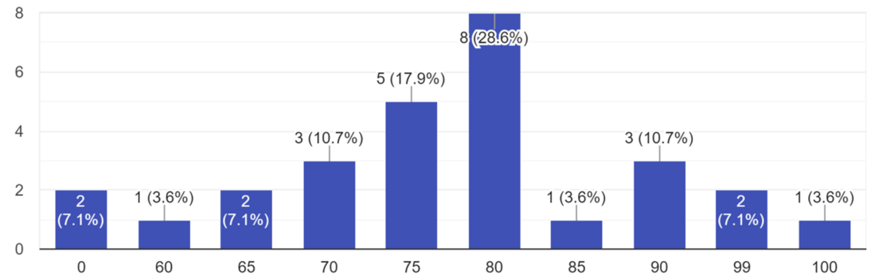

Who should be the professional figure involved in informing patients about genetic risk (and therefore referring them to a clinical geneticist)

84 responses

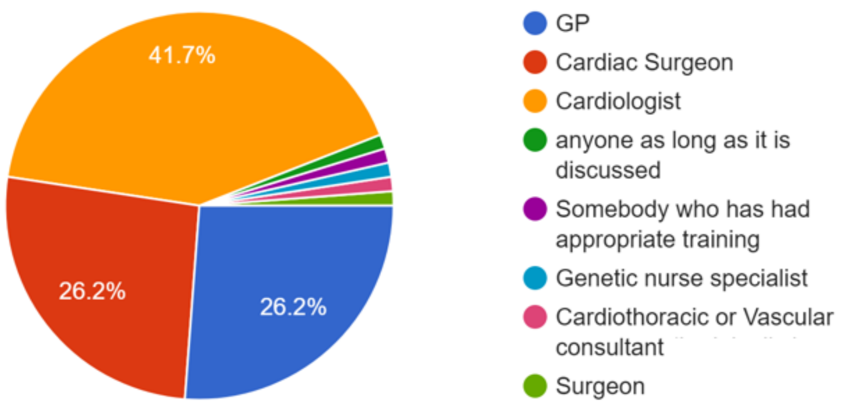

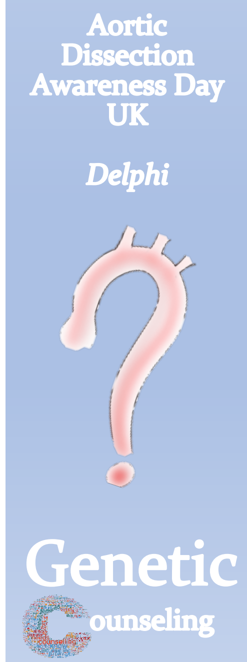

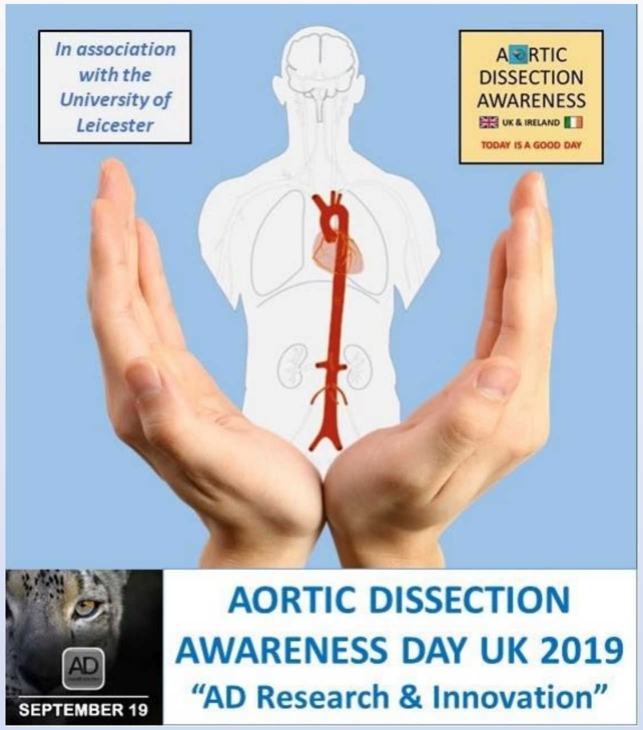

How should we look at our participants?

18 responses

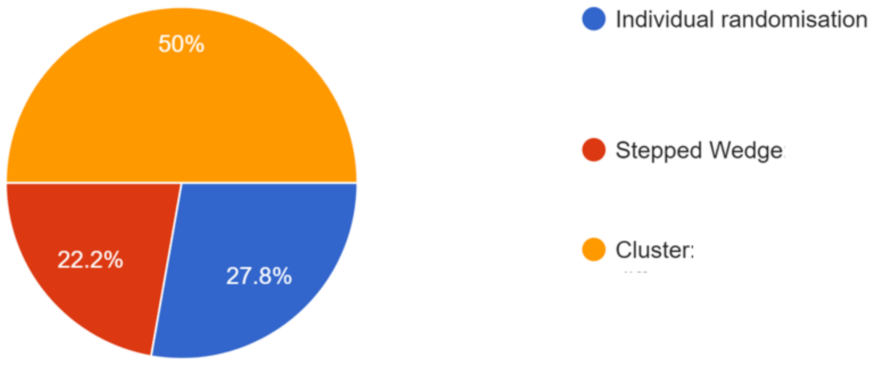

- Design effect for cluster/stepped wedge will be too penalizing.
- Very hard to implement individual randomisation where families are involved and clinical services need to be reconfigured to deliver this.

How many centres should be involved ?

15 responses

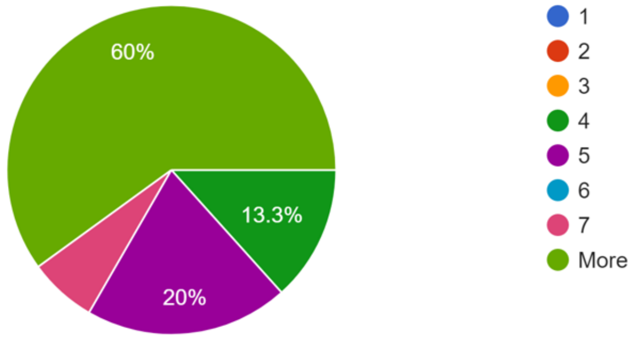

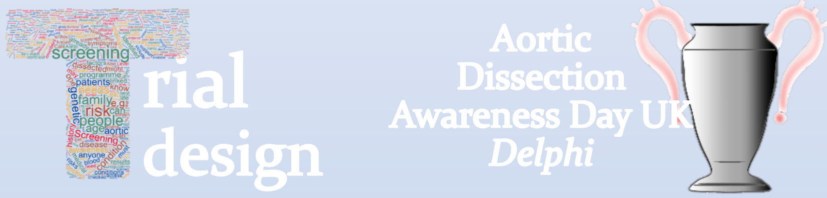

What tool should be used to measure Quality of Life?

15 responses

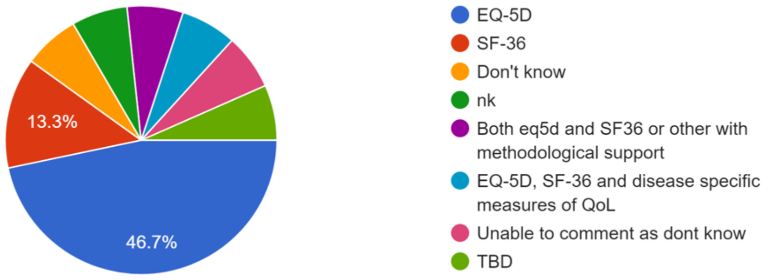

This research could expose families to a psychological stress. When should relatives involved in the test be monitored for signs of depression and anxiety?

19 responses

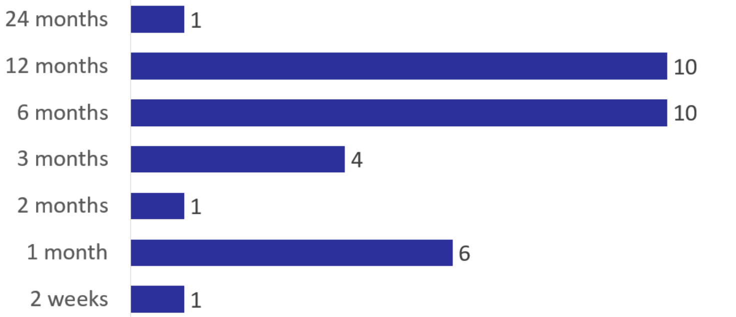

Rate the subsequent measures of effectiveness:

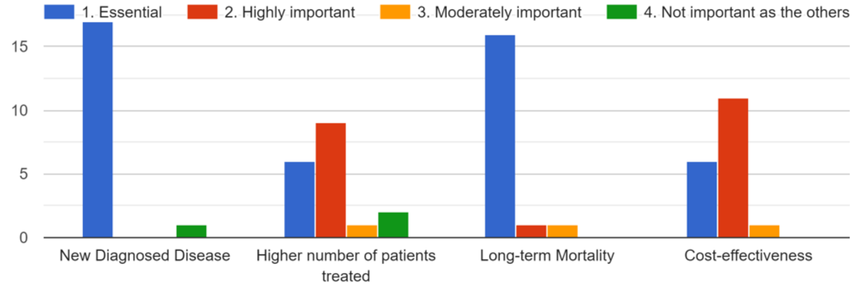

Which clinical events should be evaluated in this research? (select all relevant)

18 responses

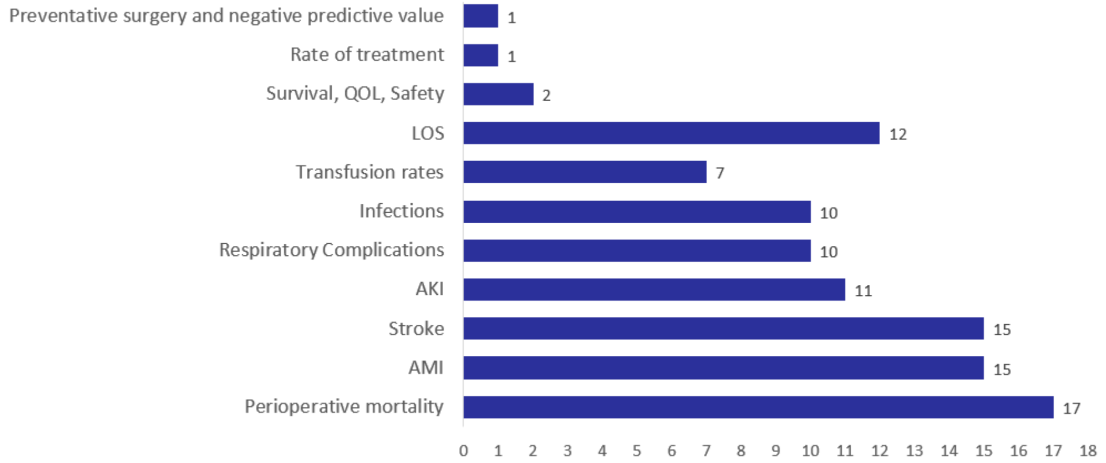

How long do you think it would take to change what is currently done for screening in your region?

14 responses

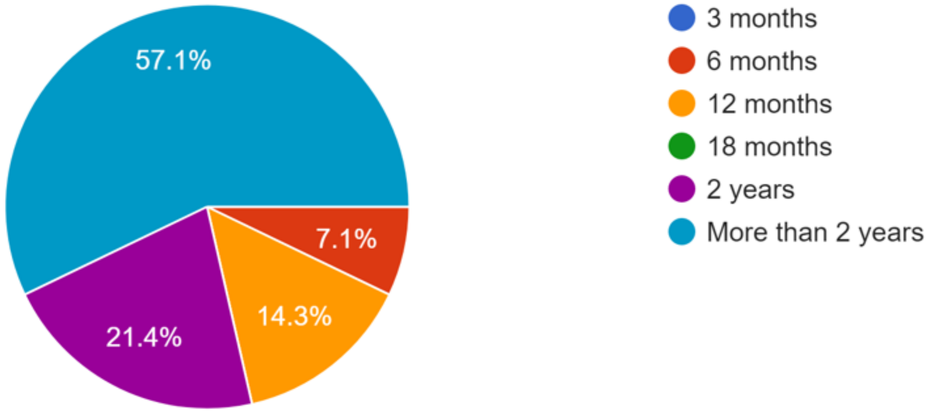

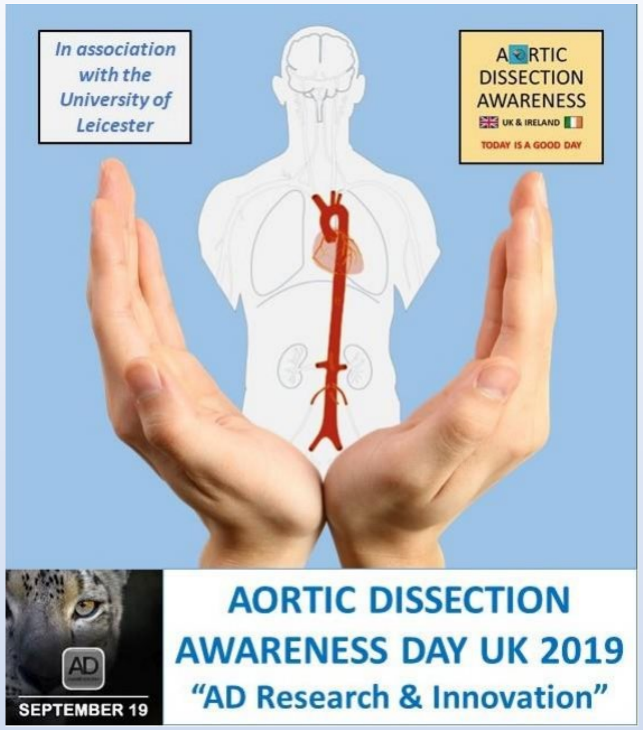
In association with the University of Leicester
AORTIC DISSECTION AWARENESS
UK & IRELAND
TODAY IS A GOOD DAY
AD
SEPTEMBER 19
AORTIC DISSECTION AWARENESS DAY UK 2019
"AD Research & Innovation"

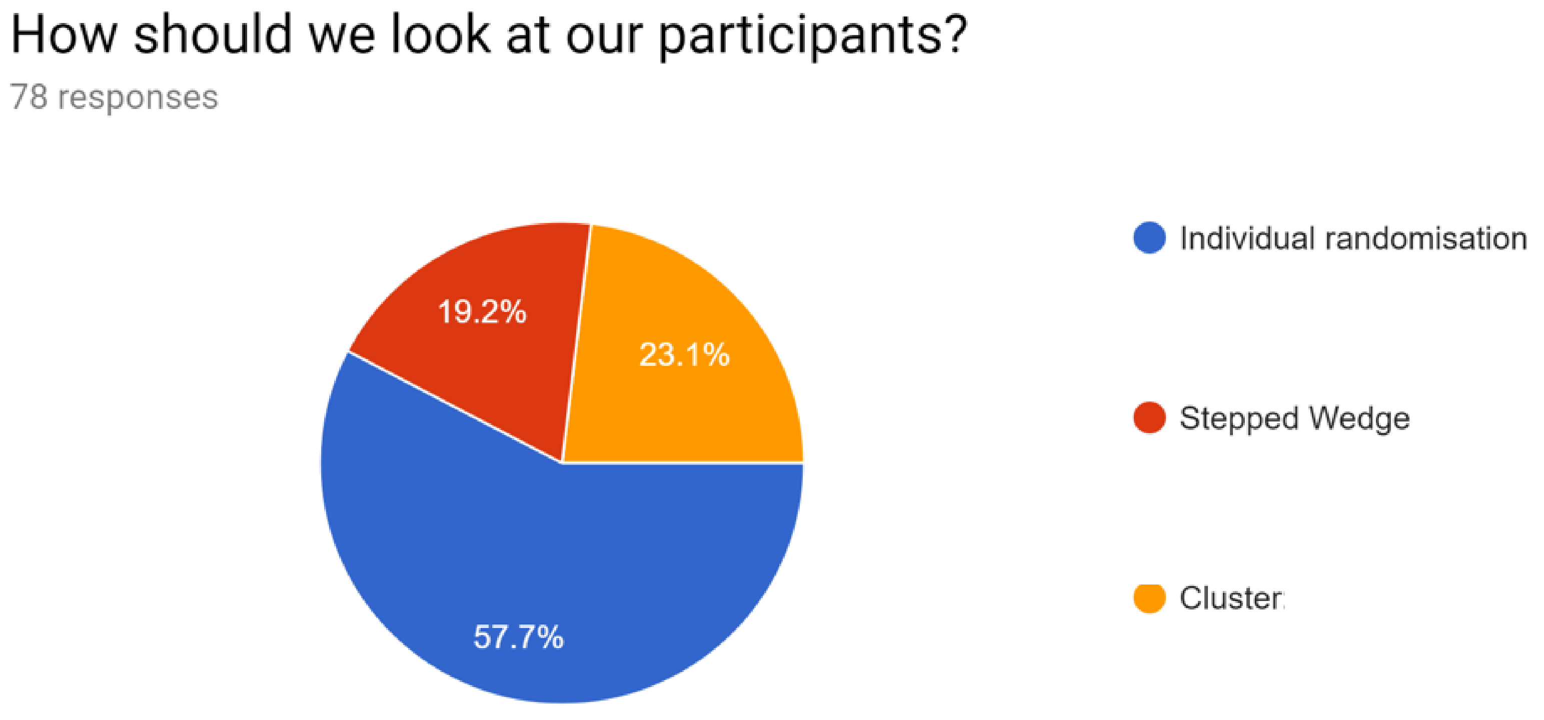
How should we look at our participants?
78 responses
Individual randomisation
Stepped Wedge
Cluster
19.2%
23.1%
57.7%

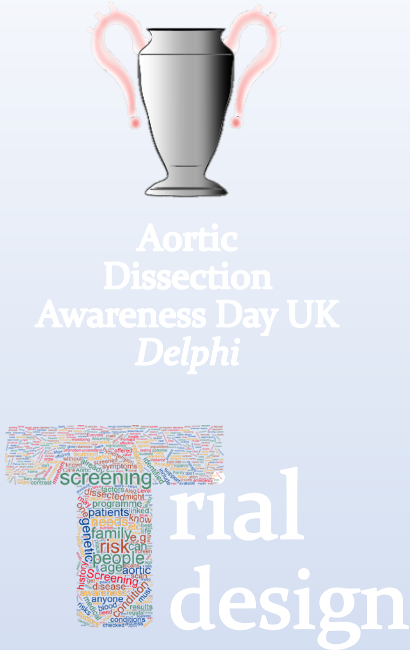
Aortic Dissection Awareness Day UK Delphi
Trial design

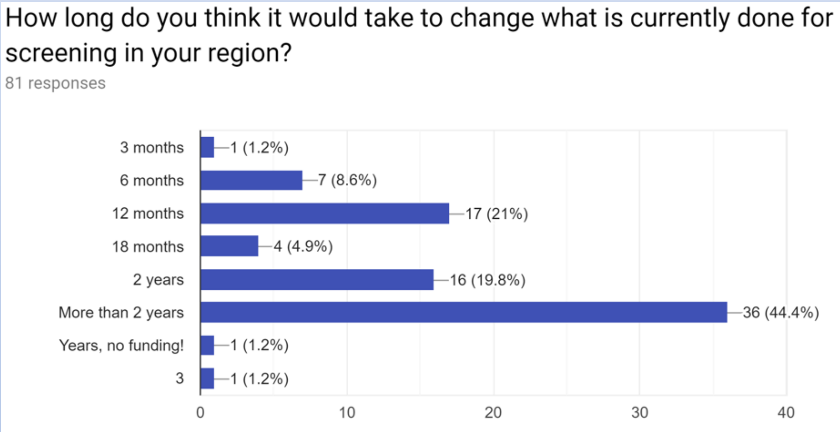
How long do you think it would take to change what is currently done for screening in your region?
81 responses
3 months
6 months
12 months
18 months
2 years
More than 2 years
Years, no funding!
3
1 (1.2%)
7 (8.6%)
17 (21%)
4 (4.9%)
16 (19.8%)
36 (44.4%)
1 (1.2%)
1 (1.2%)
0
10
20
30
40

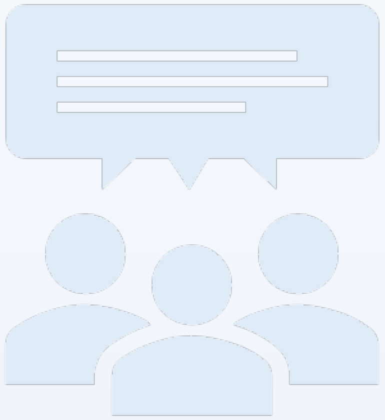

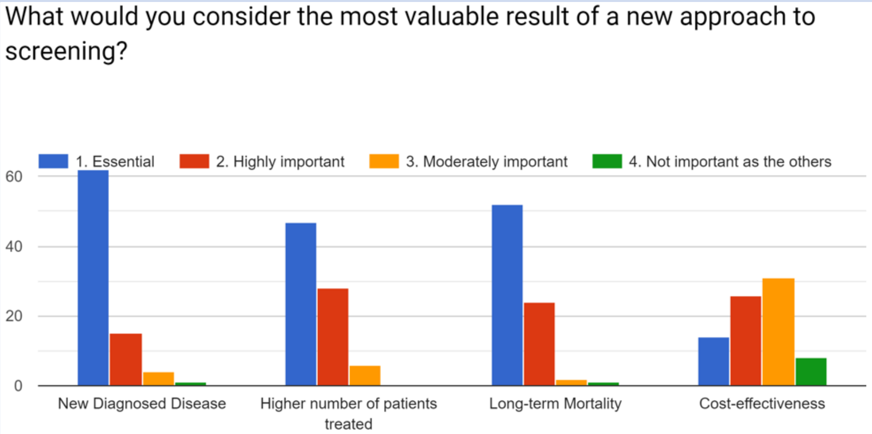
What would you consider the most valuable result of a new approach to screening?
1. Essential
2. Highly important
3. Moderately important
4. Not important as the others
60
40
20
0
New Diagnosed Disease
Higher number of patients treated
Long-term Mortality
Cost-effectiveness

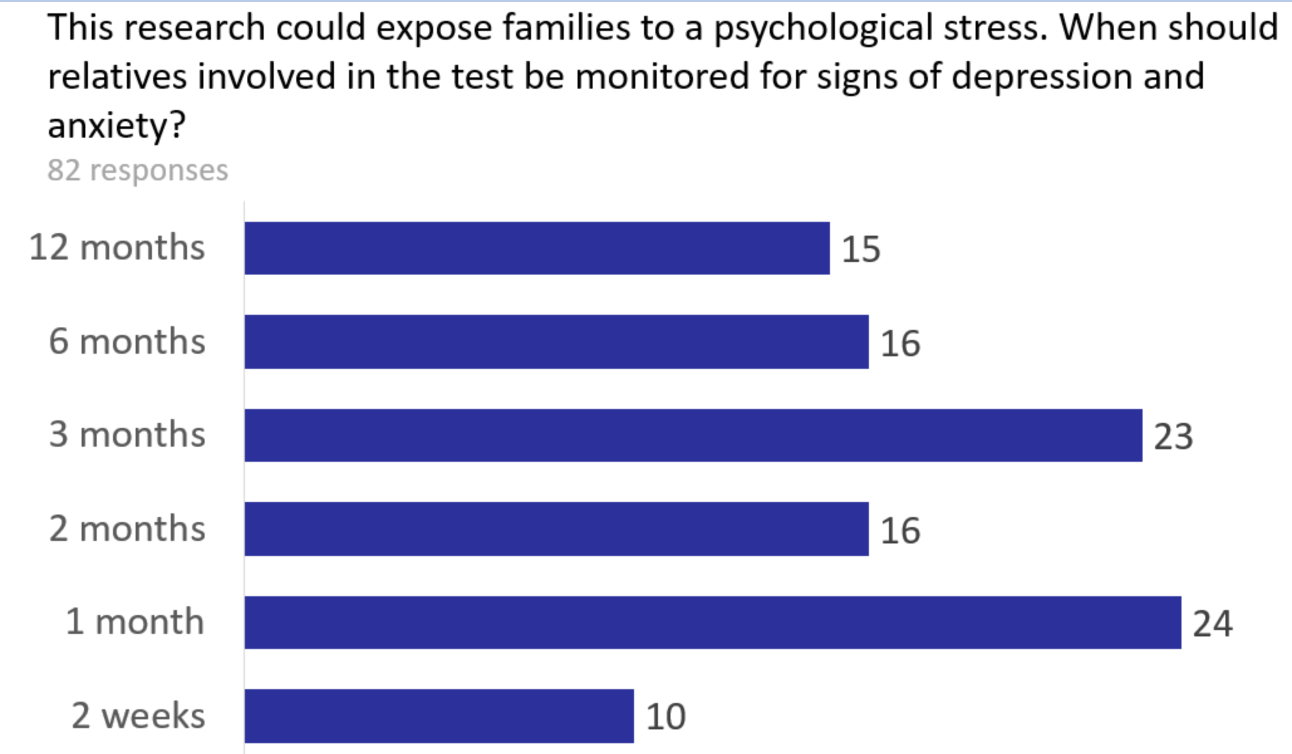
This research could expose families to a psychological stress. When should relatives involved in the test be monitored for signs of depression and anxiety?
82 responses
12 months
6 months
3 months
2 months
1 month
2 weeks
15
16
23
16
24
10

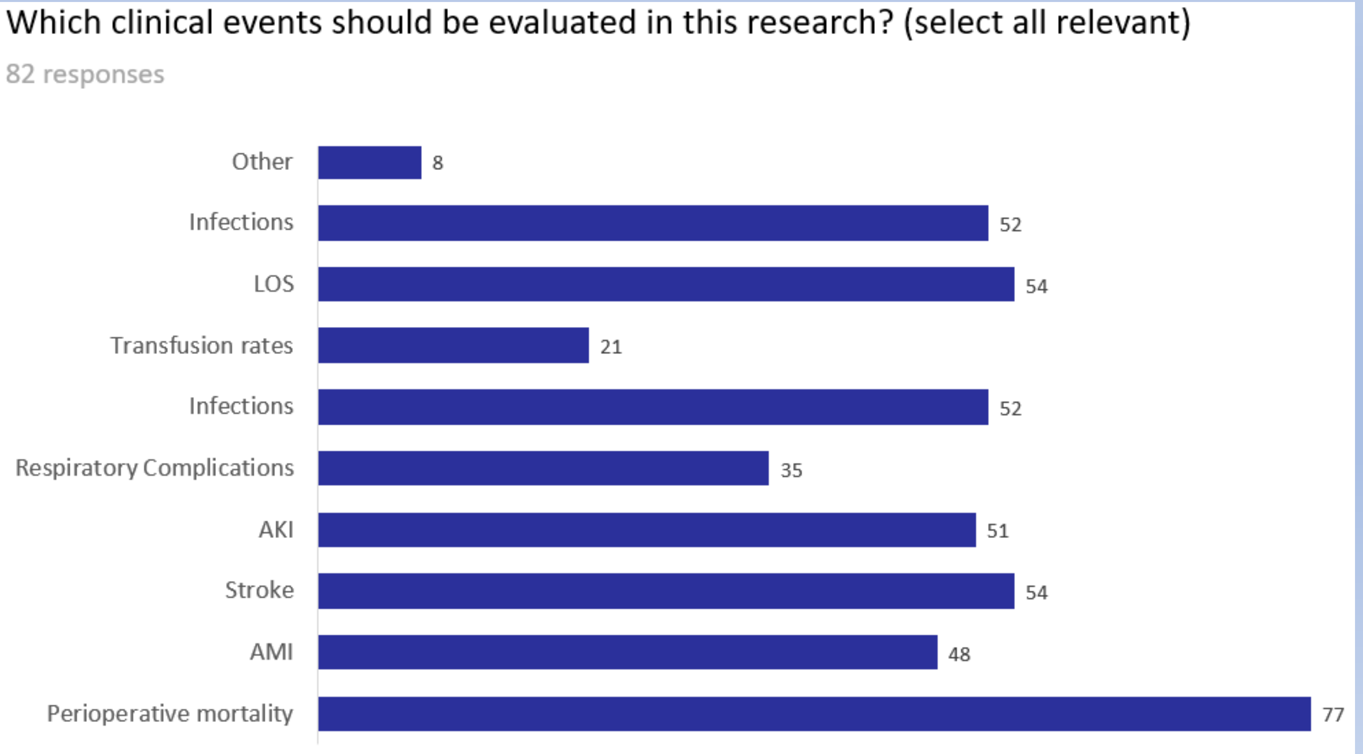
Which clinical events should be evaluated in this research? (select all relevant)
82 responses
Other
Infections
LOS
Transfusion rates
Infections
Respiratory Complications
AKI
Stroke
AMI
Perioperative mortality
8
52
54
21
52
35
51
54
48
77

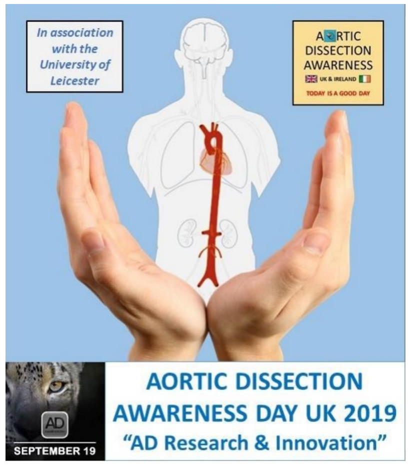

Surveillance for Thoracic Aortic Diseases

Aortic Dissection Awareness Day 2019

Dear Colleague,

Aortic Dissection Awareness UK, in partnership with a panel of clinical experts in aortic disease, is conducting a Delphi process (with full patient involvement) to explore current practice and possible future perspectives on surveillance for thoracic aortic diseases (with a focus on non-syndromic forms).

Our professional network has multidisciplinary expertise (imaging, genetics, genomic medicine, and trial design).

The Delphi process seeks to establish the requirements of a desirable screening programme, and to identify the best way to implement and to evaluate the effectiveness of their adoption. An important step in this process is determining what the standard of care is and who delivers it at the moment, hence our request for your kind collaboration with the following questions.

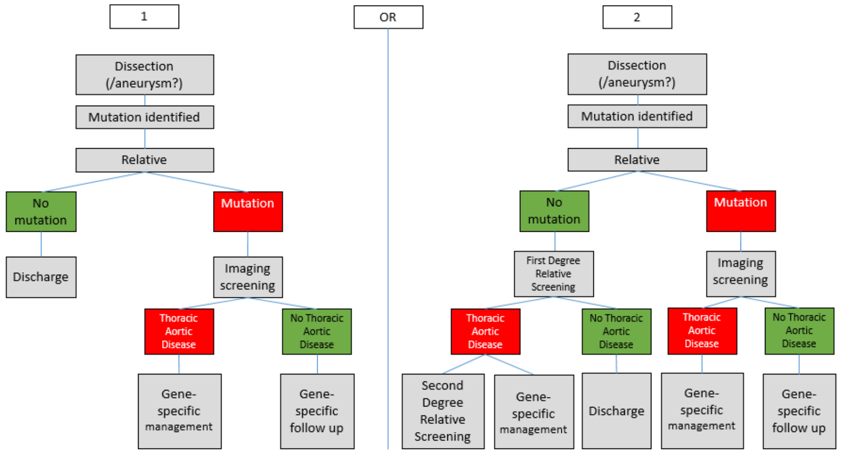

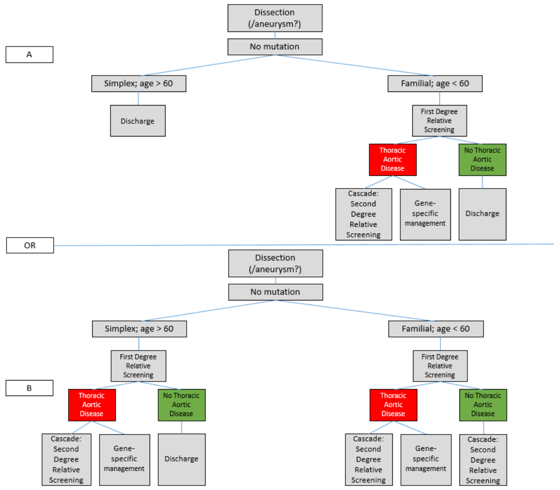

1. Which ICC Service do you work for?	2. How many patients affected by aortopathy does your Centre sees approximately each year?	3. What percentage of your workload does this number constitute?	4. What would you say is the current pathway for patients affected by thoracic aortic disease (non-syndromic) in your region?	5. In your opinion, should genetic and imaging approach be combined, or should one follow the other? How do patients' characteristics (genotype, phenotype, age, personal preferences) influence your choices in terms of timing of the different tests?	6. Based on your experience, what are the main differences in clinical genetics pathway (and in patient support needs) between this condition and the others managed in the ICCs (e.g. SADS).	7. Should a blood sample from a patient with aortic dissection be collected routinely before an urgent operation, for the purpose of genetic testing?
A	200	20%	2B	Imaging usually performed first due to no on-site genetic testing yet. Unless patient referred following finding of genetic change from screening.	Large number of patients with dilated aortas - 20% familial; 80% not familial - difficult to know how to screen and follow up. Currently most imaged and followed up annually regardless of family history or mutation or dissection. Compared to other ICC conditions where usually only followed up after a definite diagnosis.	Ideally yes, but we have not implemented this routinely yet.
B	350	40%	A	Should be combined as both equally relevant in making right diagnosis	Genetic testing is still not routine practice in many centres, more awareness in other ICCs (CMP and SADS)	Yes, blood sample and dna should be stored
C	4-500	25%	i don't think any of these capture my approach. We take age and other factors into account with the proband. We do use genetic testing, but even if we don't find something in gene panels, we appreciate that these are not 100% and when we think genetic disease is possible/likely we offer ongoing interval screening to first degree relatives (3-5 yearly). If we have a positive genetic test we use that to cascade in the usual mannner	That depends. You need to define the question better. The proband is clearly going to be a combined approach. If we have a 100% definite mutation in a family I am happy to use that data to cascade. If we have a VUS then clearly a more nuanced approach is required	The fundamental approach is very similar in 3	On the basis that they will be having other blood tests done, lines put in etc, it seems a bit short sighted not to
D	400	30%	B	Often combined, sometimes imaging leads to genetic referral and sometimes genetic referral leads to imaging and cardiology review. ...	Aortopathy (syndromic CTD (MFS, LD, EDIV) Turner etc service has cardiac specialist nurse support; given that a significant number of the patients seen with aortopathy have concomitant congenital heart disease and seen in the ACHD clinics (e.g. coarctation with bicuspid aortic valve, Fallot aorta) those patients are also supported by the ACHD CNS. The genetics pathway is better at proband identification and counselling of future risks but do not offer support for the condition and its long term management.	Yes
E	600	10%	2	Yes. In proband by definition imaging will precede genetics. In family screening both may occur simultaneously	No different in principle - identify correct phenotype, identify genotype if available, screen first degree relatives, reduce risks pertinent to phenotype / genotype / syndrome, reduce risk of acquired heart disease	Depends whose pt it is
F	100 children, much larger number of adults looked after by adult service	20%	Unable to attach it to this document.	yes should be combined, all patients ideally should see a geneticist at initial referral with echocardiogram and new patient consultation with paediatric cardiologist with expertise in aortopathy	Clinical genetics pathway can be quite separate from cardiac as often the referral will be from a paediatrician or GP straight to a geneticist whereas with other ICC, combined appointments more common. We do run a combined clinic but often patients have already seen a geneticist elsewhere. Patients have multiple other morbidities associated with Marfan and Loeys Dietz requiring many, many hospital appointments. Also heavy psychological impact due to appearance with marfanoid habitus, cannot hide diagnosis	Yes
